# Modelling hepatitis B virus infection and impact of timely birth dose vaccine: A comparison of two simulation models

**DOI:** 10.1371/journal.pone.0237525

**Published:** 2020-08-10

**Authors:** Margaret J. de Villiers, Ivane Gamkrelidze, Timothy B. Hallett, Shevanthi Nayagam, Homie Razavi, Devin Razavi-Shearer

**Affiliations:** 1 MRC Centre for Global Infectious Disease Analysis, School of Public Health, Imperial College London, London, United Kingdom; 2 Center for Disease Analysis Foundation, Lafayette, Colorado, United States of America; 3 Department of Surgery and Cancer, Imperial College London, London, United Kingdom; Centre de Recherche en Cancerologie de Lyon, FRANCE

## Abstract

Hepatitis B is a global epidemic that requires carefully orchestrated vaccination initiatives in geographical regions of medium to high endemicity to reach the World Health Organization’s elimination targets by 2030. This study compares two widely-used deterministic hepatitis B models—the Imperial HBV model and the CDA Foundation’s PRoGReSs—based on their predicted outcomes in four countries. The impact of scaling up of the timely birth dose of the hepatitis B vaccine is also investigated. The two models predicted largely similar outcomes for the impact of vaccination programmes on the projected numbers of new cases and deaths under high levels of the infant hepatitis B vaccine series. However, scenarios for the scaling up of the infant hepatitis B vaccine series had a larger impact in the PRoGReSs model than in the Imperial model due to the infant vaccine series directly leading to the reduction of perinatal transmission in the PRoGReSs model, but not in the Imperial model. Meanwhile, scaling up of the timely birth dose vaccine had a greater impact on the outcomes of the Imperial hepatitis B model than in the PRoGReSs model due to the greater protection that the birth dose vaccine confers to infants in the Imperial model compared to the PRoGReSs model. These differences underlie the differences in projections made by the models under some circumstances. Both sets of assumptions are consistent with available data and reveal a structural uncertainty that was not apparent in either model in isolation. Those relying on projections from models should consider outputs from both models and this analysis provides further evidence of the benefits of systematic model comparison for enhancing modelling analyses.

## Introduction

Hepatitis B virus (HBV), which is spread via infected bodily fluids, causes millions of chronic infections each year. The primary source of chronic HBV infections is vertical transmission from infected mothers to their infants, or horizontal transmission during early childhood. Infants and young children are especially susceptible to the infection becoming chronic. HBV causes inflammation of the liver, which can result in cirrhosis and/or liver cancer, leading to an estimated 500 000 to 700 000 deaths each year [1–3].

Although there is as yet no cure for HBV, the spread of the disease can be controlled through measures such as improved blood and injection safety and vaccination. The infant HBV vaccine series (three doses of the HBV vaccine), which provides infants with long-term protection from HBV infection [4], was included in immunization programmes in all but 12 countries by 2017 [5]. The timely birth dose of the vaccine series—the providing of the first vaccine dose within 24 hours of birth—can help reduce mother-to-child transmission. However, use of the timely birth dose vaccine is not currently widespread and does not form part of national policy in many countries in Africa [6], in part due to availability and difficulties in administering the vaccine to an infant within the recommended 24 hours after birth.

Mathematical simulation models of HBV have been used to answer many important questions. For instance, by 2017 Gavi, the Vaccine Alliance, had funded the immunization of over 404 million worldwide with the pentavalent vaccine that protects against HBV [7]. Gavi has also recently evaluated the potential impact of expanding the use of the birth dose vaccine, with a view to funding such a scale-up in future years should its replenishment be successful [8]. Furthermore, the World Health Organization (WHO) has set the goal of reducing incident HBV cases by 30% and HBV-related deaths by 10% by 2020 and incident HBV cases by 90% and HBV-related deaths by 65% by 2030, relative to 2015 levels [9]. A key part of developing and costing this strategy requires estimates of the impact of vaccine on the HBV epidemic. Some countries that have had very successful programmes may be close to achieving ‘elimination’ targets and need to evaluate the potential impact of further scale-up.

In the course of doing these analyses it has been noted that the results of the two widely used models—Imperial HBV model [10] and the CDA Foundation’s (CDAF’s) PRoGReSs model [11]—do not always exactly agree. The models have been developed separately over many years and have both been subject to multiple rounds of peer review. It is thus both interesting and important to understand when and why differences between these models arise. Following other model comparison exercises [12–14], we undertook a formal model comparison exercise with the aim of understanding the extent to which these two models produce different projections in the impact of the scale-up of infant HBV vaccination and timely birth dose in selected countries.

## Materials and methods

Two previously published models of HBV infection, the Imperial College London model (Imperial HBV model) and CDA Foundation’s PRoGReSs (PRoGReSs), were populated with the same country-specific demographic and epidemiological data for Ethiopia, India, Nigeria, and Pakistan. These are low- or lower-middle-income countries as per World Bank’s 2019 classification [15] with large population sizes as per the United Nations in their 2017 revision of World Population Prospects [16] and high (2.5%–12.2%) HBV prevalence [11]. Modifications to each model since publication are detailed in S1 Appendix.

**Fig 1:**
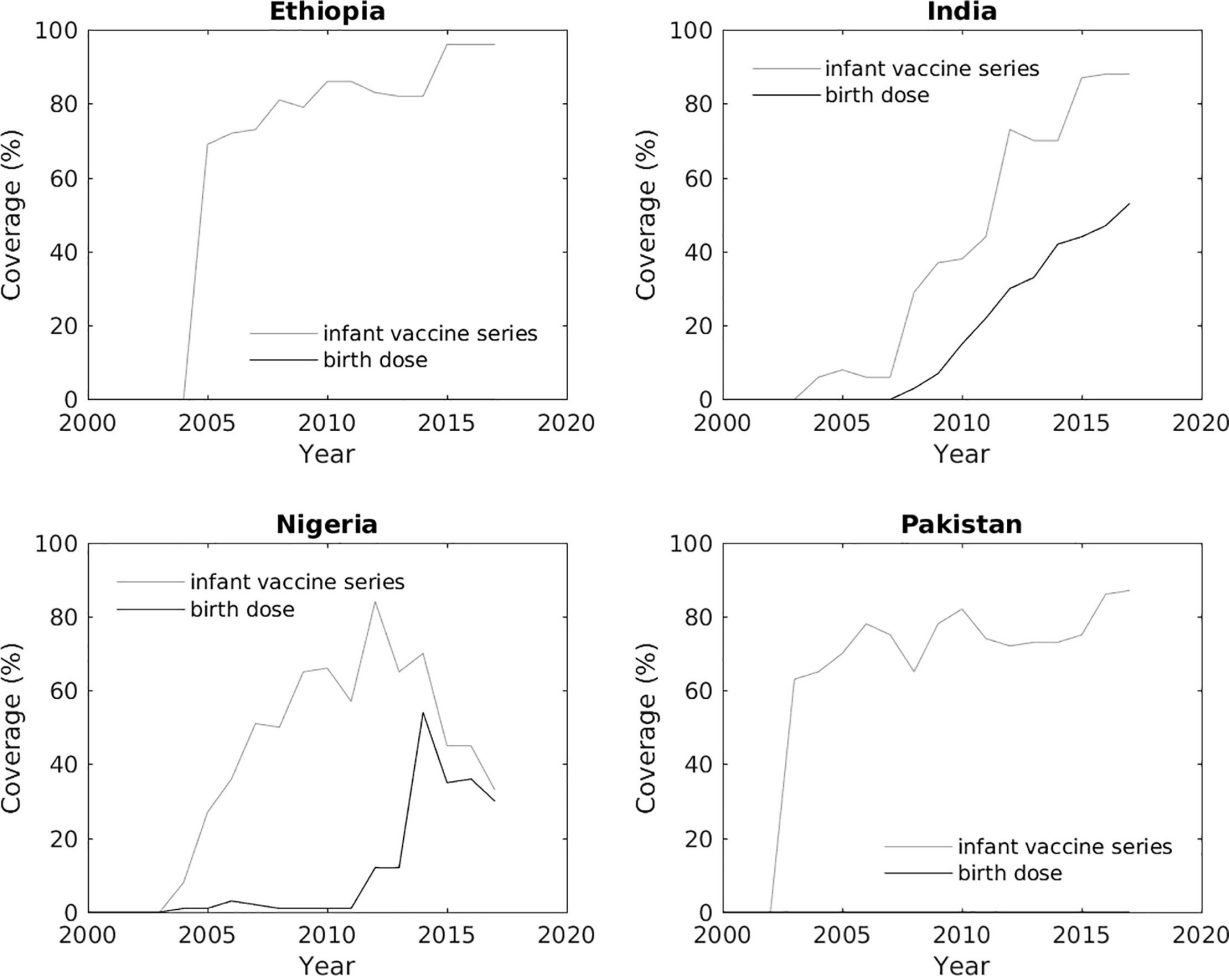
Historical coverage with timely birth dose and the infant HBV vaccine series. Coverage data were obtained from the Polaris Observatory.

For the purposes of these analyses, the demographic inputs (population, all-cause mortality rates, births or fertility rates, and male-to-female sex ratios at birth) for each country in each model were sourced from the United Nations’ 2017 World Population Prospects [16]. Historic coverage of the timely birth dose of the HBV vaccine and the infant HBV vaccine series was also set to be the same in each model for each of the four countries, as shown in Fig 1. The models incorporated the same hepatitis B surface antigen (HBsAg) and hepatitis B e antigen (HBeAg) prevalence data obtained from the Polaris Observatory [11]. This removed these factors as sources of variation between the outputs. In addition, the Imperial model incorporated data from the Global Burden of Disease (GBD) Results Tool website [17] during the fitting procedure (see S1 Appendix).

We defined three scenarios to run in each model and each country:

- Keeping the timely birth dose of HBV vaccine coverage and the infant HBV vaccine series coverage in 2017 constant at the 2017 values
- Keeping the timely birth dose of HBV vaccine coverage in 2017 constant at the 2017 value, and increasing the infant HBV vaccine series coverage to 95% of children aged 1 year by 2025 by linear interpolation over 2017–2025
- Increasing timely birth dose of HBV vaccine coverage and infant HBV vaccine series coverage to 95% by linear interpolation over 2017–2025

For each scenario, we assumed that there was no treatment for HBV infection at any time.

We examined the following sets of outputs from in each model:

- The number of incident cases of chronic HBV infection per year, over 2015–2099
- The number of HBV-related deaths per year, over 2015–2099
- The incidence rates (number of incident cases divided by total population in the respective age group) of chronic HBV infection, by age group (all ages, 0-year-olds, 1–4-year-olds, 5–14-year-olds, and 15–99-year-olds), at the mid-point of each year, over 2015–2099.

For the Imperial HBV model, outputs were generated based on 100 draws from a distribution of model parameter values for each country, determined by calibration to prevalence and death rates data (see S1 Appendix), and summarized as a mean and the 2.5 and 97.5 percentiles. In the PRoGReSs model, the base outputs were generated utilizing the base assumptions for all variables, and a Monte Carlo simulation using input uncertainty intervals and BETA-pert distribution were used to output the 2.5 and 97.5 percentiles of 100 simulations varying each of the uncertain model parameters.

In order to estimate the marginal impact of the scaling up of the infant vaccination, we examined the difference between the projections for scenarios I and II, as these differ only in the level of infant vaccination coverage that is assumed. Similarly, in order to estimate the marginal impact of the scaling up of the timely birth dose, we examined the difference between the projections for scenario II and III, as these differ only in respect of the level of timely birth dose coverage that is assumed.

The Imperial model was run in MATLAB [18], whereas the PRoGReSs model was run in Microsoft Office Excel [19] and Microsoft Visual Basic for Applications [20].

## Results

The impact of scaling up the birth dose coverage from status quo in 2017 to 95% from 2025 onwards (i.e., the difference between scenarios II and III) on the incident cases of chronic HBV and HBV-related deaths averted between 2015 and 2099 is shown in Table 1. The impact of scaling up infant vaccine series and birth dose vaccination from status quo in 2017 to 95% (i.e., the difference between scenarios I and III) from 2025 onwards on the incident cases of chronic HBV and HBV-related deaths averted between 2015 and 2099 is shown in Table 2. The results in 2020 and 2030 of scaling up both the infant and birth dose coverage to 95% (scenario III; similar to the WHO targets of scaling up infant and birth dose coverage each to 90% by 2030 [9]) on the percentage reduction in chronic HBV cases and HBV-related deaths is shown in Table 3.

**Table 1:** CHB cases and deaths averted as a result of scaling up HBV birth dose vaccination.

	Incident CHB cases averted between 2015 and 2099 as a result of scaling up the birth dose coverage from status quo in 2017 to 95% from 2025 onwards		HBV-related deaths averted between 2015 and 2099 as a result of scaling up the birth dose coverage from status quo in 2017 to 95% from 2025 onwards	
Country / Model	Imperial	PRoGReSs	Imperial	PRoGReSs
Ethiopia	1 441 000 (456 000–2 261 000)	138 000 (32 700–163 000)	146 000 (52 000–217 000)	14 000 (3 900–19 400)
India	1 346 000 (343 000–2 607 000)	137 000 (34 600–162 000)	212 000 (63 900–377 000)	18 200 (5 100–24 800)
Nigeria	11 098 000 (4 724 000–17 195 000)	509 000 (139 000–628 000)	538 000 (251 000–807 000)	34 100 (10 500–49 600)
Pakistan	725 000 (262 000–1 767 000)	80 400 (24 400–119 000)	130 000 (58 100–281 000)	10 000 (3 300–15 800)

CHB: chronic hepatitis B; HBV: hepatitis B virus.

**Table 2:** CHB cases and deaths averted as result of scaling up HBV infant vaccine series and birth dose vaccination.

	Incident CHB cases averted between 2015 and 2099 as a result of scaling up the infant & birth dose coverage from status quo in 2017 to 95% from 2025 onwards		HBV-related deaths averted between 2015 and 2099 as result of scaling up the infant & birth dose coverage from status quo in 2017 to 95% from 2025 onwards	
Country / Model	Imperial	PRoGReSs	Imperial	PRoGReSs
Ethiopia	1 423 000 (448 000–2 236 000)	106 000 (440–130 000)	145 000 (51 200–215 000)	10 200 (0–14 700)
India	1 470 000 (388 000–2 810 000)	410 000 (302 000–449 000)	231 000 (72 400–405 000)	57 900 (39 100–75 800)
Nigeria	31 470 000 (18 588 000–39 843 000)	91 805 000 (87 029 000–110 748 000)	1 200 000 (872 000–1 560 000)	3 649 000 (2 671 000–5 628 000)
Pakistan	815 000 (300 000–1 966 000)	196 000 (147 000–285 000)	145 000 (66 300–310 000)	25 700 (19 100–39 900)

CHB: chronic hepatitis B; HBV: hepatitis B virus.

**Table 3:** Reduction in CHB cases and deaths as result of scaling up HBV infant vaccine series and birth dose vaccination.

		Incident cases of CHB (reduction relative to 2015 levels)			HBV-related deaths (reduction relative to 2015 levels)		
Country	Model	2015	2020	2030	2015	2020	2030
**Ethiopia**	Imperial	65 900	41 800 (37%)	5 100 (92%)	6 500	7 700 (-18%)	10 700 (-65%)
	PRoGReSs	48 300	30 100 (38%)	15 600 (68%)	9 200	10 900 (-18%)	15 100 (-63%)
**India**	Imperial	183 000	77 600 (58%)	17 400 (91%)	75 500	84 100 (-11%)	102 000 (-35%)
	PRoGReSs	140 000	74 200 (47%)	28 500 (80%)	78 700	89 400 (-14%)	112 000 (-42%)
**Nigeria**	Imperial	403 000	425 000 (-6%)	47 300 (88%)	18 700	21 900 (-17%)	29 800 (-59%)
	PRoGReSs	614 000	606 000 (1%)	95 200 (84%)	25 300	28 800 (-14%)	33 900 (-34%)
**Pakistan**	Imperial	54 200	34 700 (36%)	4 200 (92%)	12 600	14 800 (-17%)	19 000 (-50%)
	PRoGReSs	37 400	19 700 (47%)	7 000 (81%)	11 500	12 500 (-8%)	14 200 (-23%)

CHB: chronic hepatitis B; HBV: hepatitis B virus.

**Fig 2:**
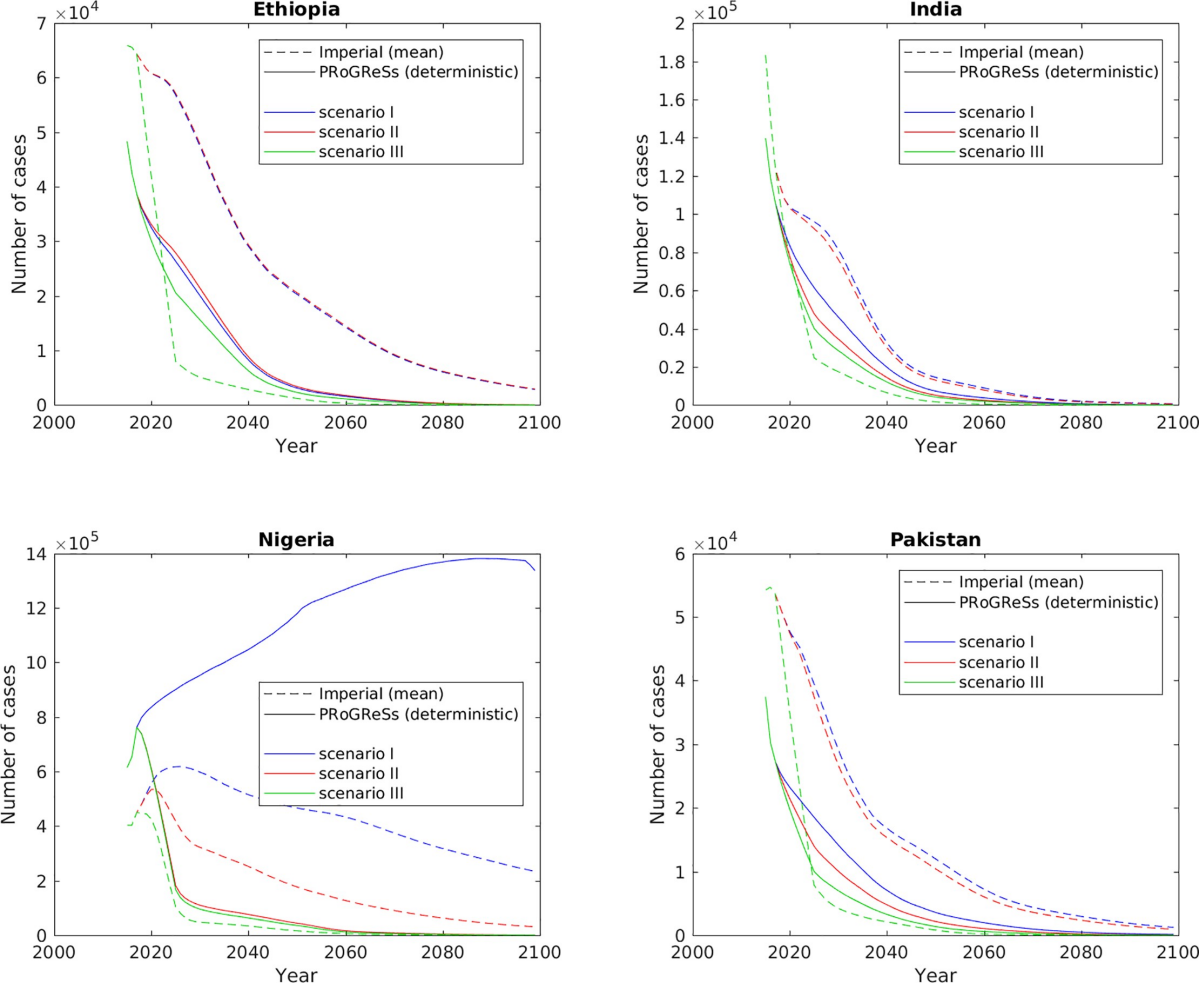
Incident cases of chronic HBV infection per year between 2015 and 2099.

**Fig 3:**
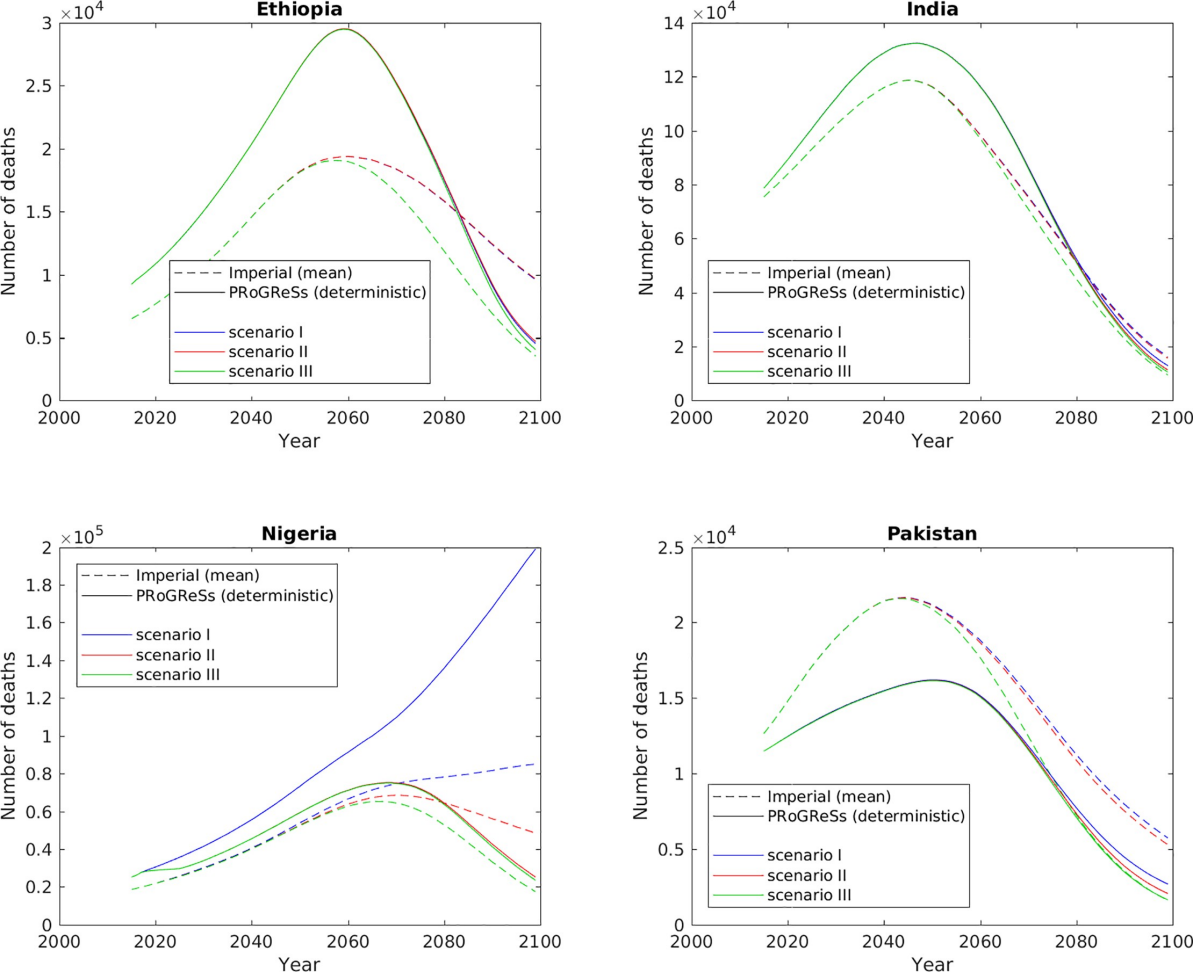
HBV-related deaths per year between 2015 and 2099.

Figs 2 and 3 show the incident chronic HBV cases and HBV-related deaths, respectively, from 2015 to 2099 for the two models for all three scenarios. S6 and S7 Figs in the supplementary information show the same outcomes, but with confidence bands showing the 2.5 and 97.5 percentiles. For each of the scenarios I–III, the two models yield largely similar results in terms of scale and trends. PRoGReSs outputs lie within the confidence bands of the Imperial model results, with the exception of Nigeria (S6 and S7 Figs): under scenario I, Imperial outputs 36.3 million (21.8–45.8 million) incident cases of chronic HBV infection over 2015–2099, while PRoGReSs outputs 99.7 million (94.4–119.9 million), a 175% difference at baseline; under scenario III, Imperial outputs 4.9 million (3.2–6.0 million) while PRoGReSs outputs 7.9 million (7.5–9.5 million), a 63% difference at baseline.

**Fig 4:**
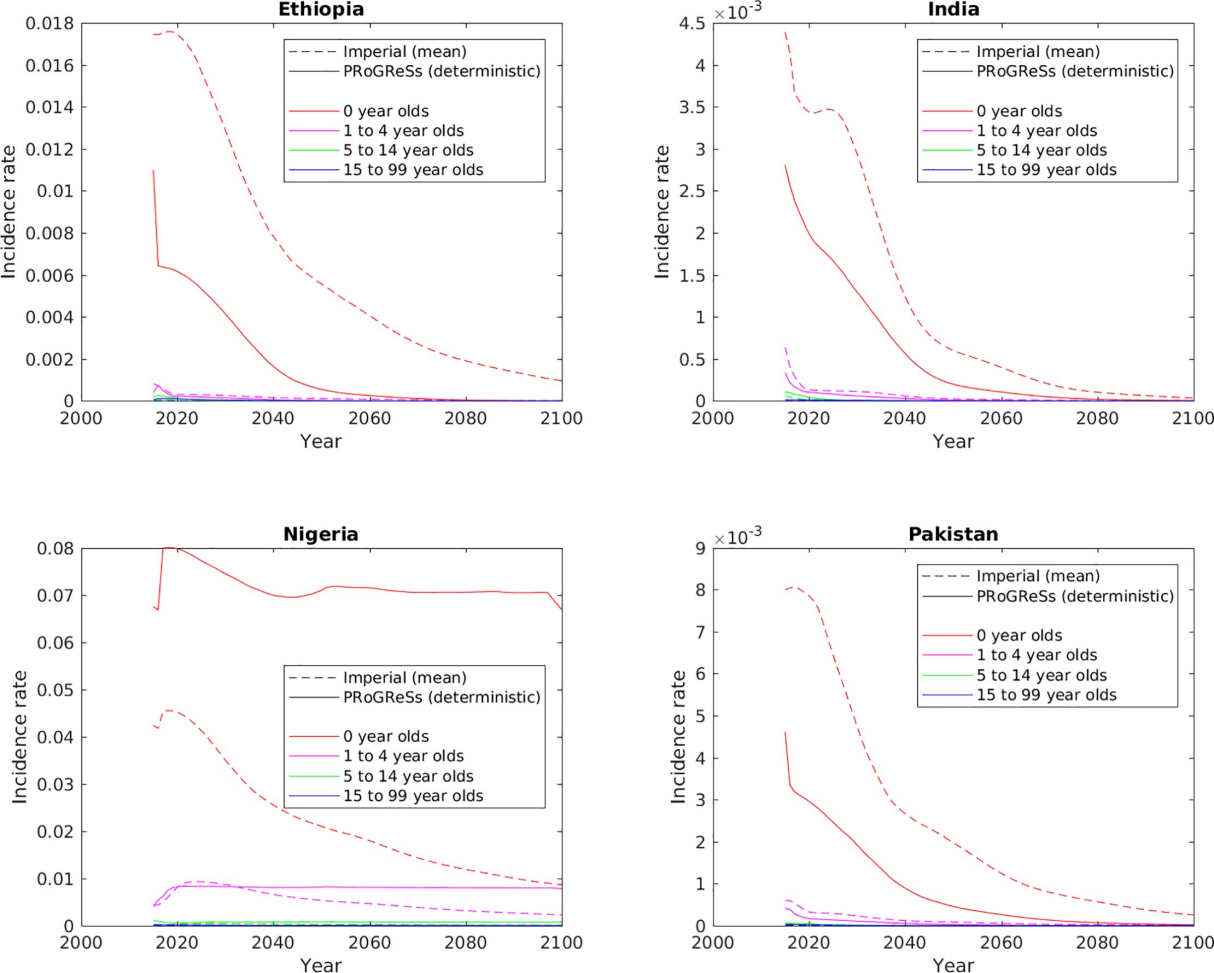
Incidence rates of chronic HBV infection in scenario I in four age groups.

In order to understand the differences between the two models better, incident cases of chronic HBV infection in different age groups were analysed. Fig 4 shows the incidence rates of chronic HBV infections in scenario I of each country partitioned amongst 0-year-olds, 1–4-year-olds, 5–14-year-olds, and 15–99-year-olds. This figure reveals that the delay in incidence of chronic HBV in response to vaccination evident in the Imperial results in Fig 2 is due to the effect of the vaccination schedule on the infant population.

**Fig 5:**
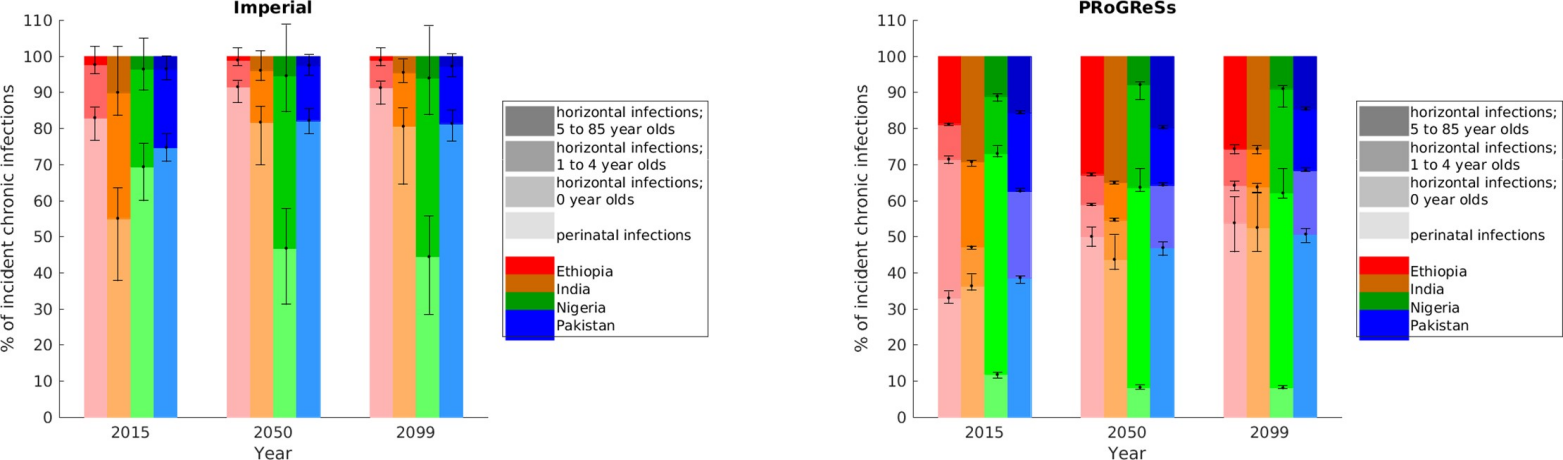
Percentage incident chronic HBV cases in scenario I due to perinatal and horizontal infections.

We looked at the pattern of transmission in each model by breaking down cases of new chronic carriage by the age and mode of acquisition (Figs 5 and S9). Perinatal infections (HBV infection acquired from the mother during pregnancy, childbirth or close contact during the first year of life, e.g. during breastfeeding) constitute a greater proportion of total incident chronic HBV infections in all four countries and scenarios in the Imperial results than in the PRoGReSs results. It is also evident in Figs 5 and S9 that, in all countries and scenarios, a greater proportion of incident chronic HBV infections (perinatal and horizontal) occur in 0–4-year-olds compared to in the older age groups in the Imperial results relative to the PRoGReSs model results. Note that the Imperial model assumes no risk of horizontal transmission during the first year of life.

## Discussion

Both models predict reductions in incident chronic HBV cases roughly in line with the WHO goals of a 30% reduction by 2020 and a 90% reduction by 2030 from the baseline in 2015 [9] in scenario III in the four countries in Table 3. However, neither model reaches the WHO targets of 10% and 65% reductions in HBV-related deaths by 2020 and 2030, respectively. Indeed, both models predict increases in HBV-related deaths over the next few decades. This sustained increase in deaths is because HBV vaccination programmes were only initiated in these four countries between 2000 and 2005 (see Fig 1), and it can take decades for a chronic HBV infection to result in a life-threatening illness such as cirrhosis and liver cancer. Moreover, in addition to target coverage values, the WHO recommendations also include other preventative measures such as blood and injection safety, as well as treatment targets [9], none of which have been included in the current modelling exercise. Inputting treatment programmes into the models might result in the number of HBV-related deaths projected dropping dramatically.

The largest difference between the two models for scenario I was in Nigeria (Figs 2, 3, S6 and S7). As shown in Fig 1, the infant series vaccination coverage dropped starting in 2013, with vaccination coverage at 33% in 2017. The birth dose vaccination also declined to 30% by 2017. At a constant vaccination coverage, after 2017 the PRoGReSs model forecasts an increasing number of chronic infections while the Imperial model projects an increase followed by drop in the number of chronic infections (Fig 2). The impact of expanded vaccination (scenario III) is higher in the PRoGReSs model as shown in Fig 2.

The comparison of the scenarios provides important insights into the differences between the models:

1. The impact of the expansion of the infant HBV vaccine series coverage (i.e., the difference between scenarios I and II) is estimated to be much larger in the PRoGReSs model than in the Imperial model (Figs 2, 3, S6 and S7). The PRoGReSs model follows the HBV vaccine clinical trial results that show a large drop in HBV transmission in the first year of life as result of infant vaccine series in the absence of birth dose vaccination [21].
2. The aforementioned trend is the case in India, Nigeria, and Pakistan over 2015–2099. The same is not observed in Ethiopia because infant vaccine coverage at status quo was very high (96%), making scenarios I and II almost identical in their definition for Ethiopia. S8 Fig shows that in both models there is an approximately linear relationship between the percentage point increase in infant vaccine coverage and the reduction in incident cases of chronic HBV infection, with the slope of the linear approximation in PRoGReSs being over one-and-a-half times that of the Imperial model (see S1 Text for statistics from the regression analyses).
3. The impact of scaling up birth dose coverage (i.e. differences between scenarios II and III) is larger in the Imperial model compared to PRoGReSs (Table 1 and Figs 2, 3, S6 and S7). This is the case in all four countries studied.
4. There is a delay of several years in the effect of the infant HBV vaccine series on incident chronic HBV cases in scenarios I and II of the Imperial results compared with those of the PRoGReSs results (Fig 2).

A greater proportion of transmission being perinatal in the Imperial model partly explains why the model shows a greater impact of birth dose and smaller impact of the infant vaccine series: in the Imperial model, only timely birth dose can avert perinatal infections, which accounts for the bulk of transmission (point (1) above). In the PRoGReSs model, the addition of timely birth dose to the infant vaccine series reduces the risk of perinatal transmission by over half (relative to infant vaccine alone; see Table E in S1 Appendix). However, the infant vaccine series alone not only reduces the risk of perinatal transmission, as defined above, by about 70% compared to no intervention, but also results in the infant no longer being susceptible. In all countries analysed, a plurality of incident cases occurs in the first year of life and the majority of chronic cases occur within the first four years of life in the PRoGReSs model. The combination of the reduction of perinatal cases and the immunity provided by the infant vaccine series has a larger impact on new cases than the additional benefit of timely birth dose in the PRoGReSs model (points (1) and (2) above). In contrast, in the Imperial model infants only gain immunity from HBV from the infant vaccine series at 6 months of age. Hence, immunity due to the infant vaccine series cannot prevent perinatal infection, which is modelled to occur at birth in the Imperial model.

This difference in the effect of the infant vaccine series in the PRoGReSs versus the Imperial models is particularly evident in the large divergence in model outcomes for Nigeria in scenario I in Figs 2, 3, S6 and S7, S8 Fig shows that reducing the infant vaccine coverage from 2025 onwards in Nigeria from the level in scenario II (95%) to that in scenario I (33%) results in a greater difference in incident chronic HBV infections in the PRoGReSs model between the two scenarios than it does in the Imperial model, resulting in the model outputs showing greater divergence in scenario I than in scenario II between the two models in Figs 2, 3, S6 and S7. This is consistent with the age-pattern of differences (Fig 4), which shows that the two models differ to the greatest extent in the youngest age group (0-year-olds; red lines in Fig 4) in scenario I in Nigeria, with incidence rates being much higher in the PRoGReSs model than in the Imperial model.

Both models take into account the secondary impact of vaccination—women of childbearing age who were vaccinated as infants will have a lower prevalence. In the PRoGReSs model, the initial impact of infant vaccination on 0-year-olds is larger, and the continuing decrease in new cases is more gradual over time. The curve changes shape once those first infants vaccinated become mothers (Fig 4). The gradual decrease is a result of the assumption that horizontal transmission occurs among those aged <1 and that children can be infected by both their peers and caretakers. Thus, as the prevalence in this cohort decreases, the probability of infection does as well, culminating in children and mothers having been vaccinated. As a greater proportion of infections are perinatal in the Imperial model and there is no horizontal transmission among 0-year-olds, the initial drop in new chronic cases among 0-year-olds is much smaller but the decrease in cases over time is more rapid.

**Fig 6:**
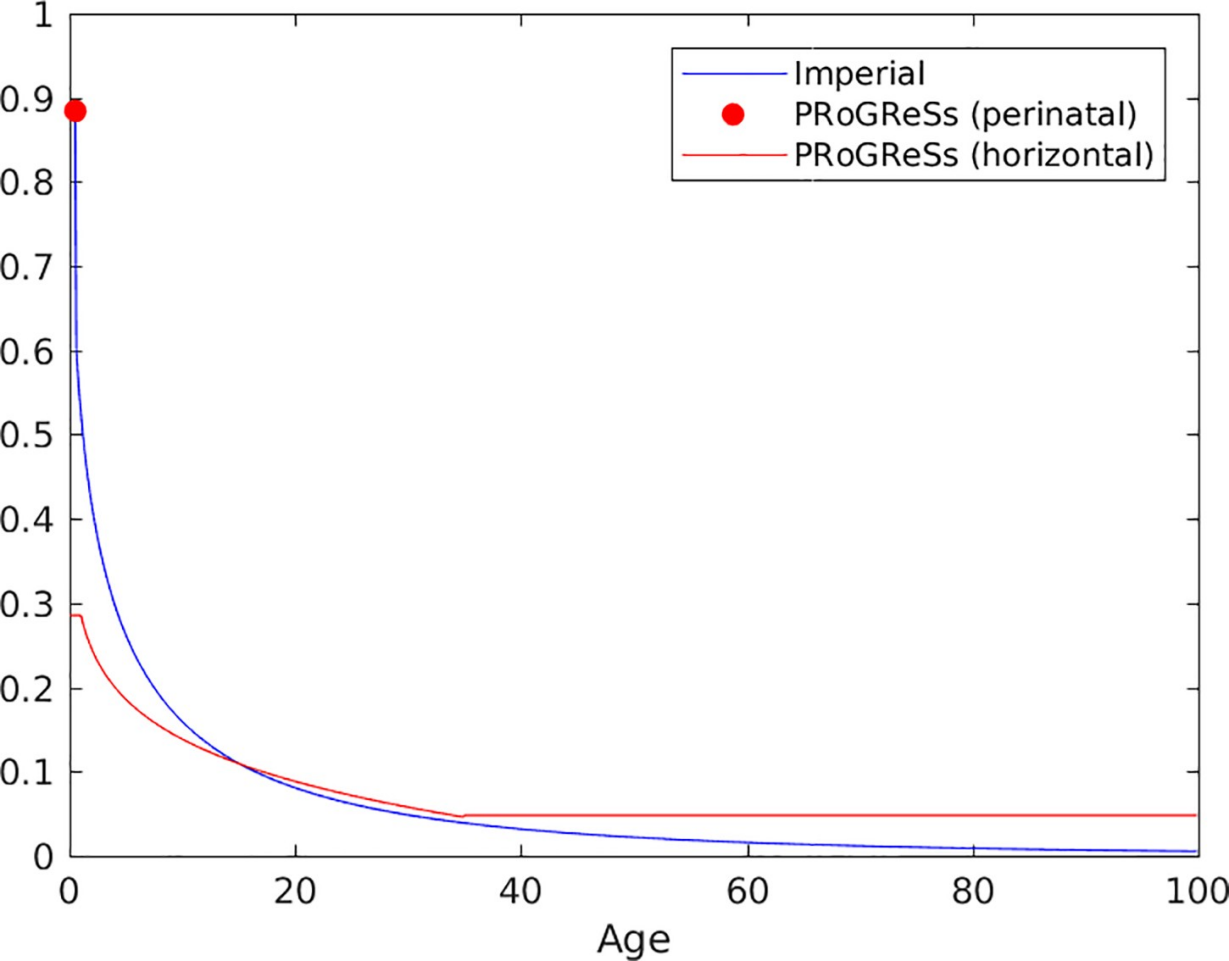
The risk of acute HBV developing into chronic HBV in the two models.

The reason for differences in the proportion of incident chronic cases occurring in infants is related to different assumptions in the models. First, that the models make different assumptions on the risk of chronic carriage given infection (Fig 6). The risk for development of chronic HBV infection in the Imperial model falls at a faster rate than in the PRoGReSs model, with the risk for chronicity in PRoGReSs surpassing that in Imperial at approximately age 15. Second, the Imperial model assumes that during the first year of life, infants are not at risk of horizontal transmission. Hence, the only means by which an infant can be infected is through perinatal transmission. Therefore, in order for the models to match the same prevalence data, a greater proportion of infections must arise through perinatal infection in the Imperial model. Third, the models calculate the number of perinatal infections differently, with the calculation dependent on the proportion of HBeAg-positive individuals in the Imperial model and on the proportion of individuals with high viral load in the PRoGReSs model. Whereas in the Imperial model both HBeAg-positive and HBeAg-negative, HBsAg-positive individuals contribute to the number of perinatal infections (see Table D in S1 Appendix), in the PRoGReSs model only individuals with a high viral load contribute towards perinatal infections (see Table E in S1 Appendix), resulting in fewer perinatal infections in populations with large proportions of individuals with low viral loads. Moreover, as mentioned previously, whereas in the Imperial model only birth dose coverage is used to calculate the number of perinatal infections, in the PRoGReSs model both birth dose and infant coverage are used to determine the number of perinatal infections. Furthermore, the birth dose vaccine leads to a greater reduction in mother-to-child transmission (MTCT) rates in the Imperial model than in the PRoGReSs model. More specifically, in the Imperial model infants receiving the birth dose vaccine have much lower rates of MTCT than do those not receiving the birth dose vaccine (15.3% versus 90% in the case of HBsAg-positive, HBeAg-positive mothers; for HBsAg-positive, HBeAg-negative mothers, the MTCT rate for infants receiving the birth dose vaccine is 5% of the MTCT rate of infants not receiving the birth dose vaccine). In contrast, in the PRoGReSs model, MTCT rates are 13.8% for infants receiving both the birth dose and infant vaccines versus 32.7% for infants receiving the infant vaccine only amongst infants with high-viral load mothers. Also notice that the rate of MTCT amongst infants of high-viral load mothers receiving both the birth dose and infant vaccines in the PRoGReSs model is similar to the MTCT rate in infants of HBsAg-positive, HBeAg-positive mothers receiving the birth dose in the Imperial model (13.8% in the PRoGReSs modes; 15.3% in the Imperial model).

Thus, we hypothesised that the differences in these assumptions between the two models explains the different patterns of infection (more perinatal transmission in the Imperial model), and the different results for impact. As a test of this hypothesis, we modified the Imperial model in the following ways, with the expectation that doing so would cause the estimates of impact between the PRoGReSs model and the (modified) Imperial model to become more similar.

- use the PRoGReSs function for the risk of chronic infection,
- allow horizontal infections in the first year of life, and
- use the PRoGReSs calculation for the number of perinatal infections.

**Fig 7:**
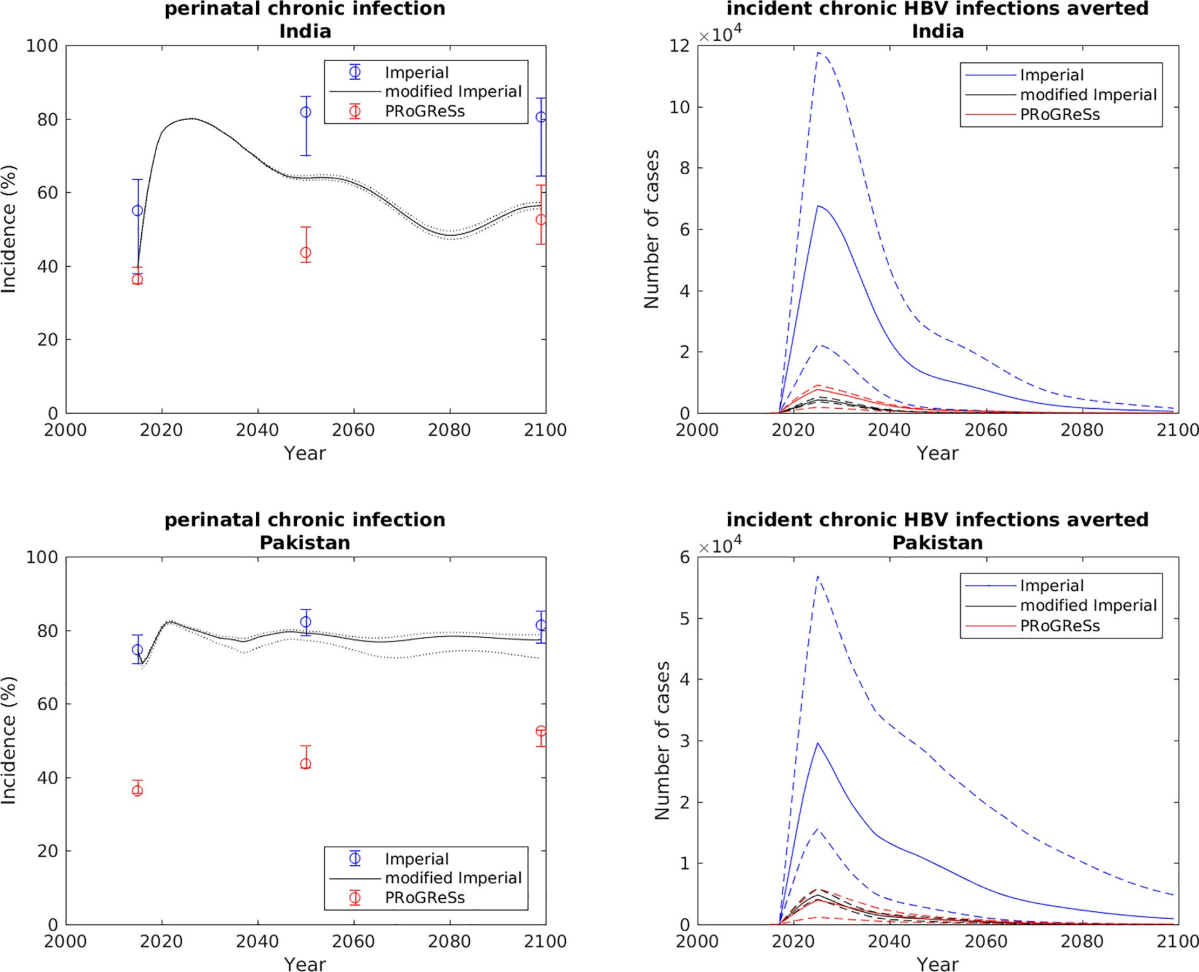
Perinatal HBV infections in scenario I and CHB infections averted between scenarios II and III in India and Pakistan in the Imperial model, the PRoGReSs model and the modified Imperial model. CHB: chronic hepatitis B; HBV: hepatitis B virus.

Doing this for India and Pakistan indeed resulted in outputs from the modified Imperial model that were more similar to those from the PRoGReSs model, as is evident in the percentage of incident HBV cases due to perinatal infection in scenario I and the number of chronic HBV infections averted due to the scaling up of the birth dose coverage between scenarios II and III in Fig 7.

Two models that have been widely used to estimate the impact of HBV vaccination tend to produce results that are broadly similar, but exhibit important discrepancies in some cases. We aimed to understand what lies behind these differences.

We find that three important assumptions differ between the models and underlie much of the differences in the model results that have been noted. These are:

- The Imperial model assumes that infants are not exposed to horizontal infection whereas PRoGReSs does.
- The Imperial model assumes that risk of chronic carriage is more concentrated at younger ages than in PRoGReSs.
- The Imperial model only uses HBeAg status and birth dose coverage to determine the number of perinatal infections; PRoGReSs uses viral load and both infant coverage and birth dose coverage to determine the number of perinatal infections, with an increase in birth dose coverage having a smaller effect on reducing perinatal infections than an increase in infant coverage.

In each case, the different assumptions are arguably defensible. The risk and source of transmission of HBV at different ages is not well understood. There may be reasons to believe that the risk of infection for infants is heavily dominated by the exposure to their mother, but it is also conceivable that infants are as exposed to other infants and children as those at older ages. Similarly, the data on chronic carriage are sparse and would be broadly supportive of a wide range of relationships between age and the risk of chronic carriage. Both models have alighted on the same data and represented them in different ways—and these small differences have propagated to have an important influence on such a crucial part of the model. Moreover, the knowledge of the effectiveness of birth dose, and on what it depends, is limited, especially in Africa, but seems to vary by the viral load of the mother, which is seemingly well approximated by the HBeAg status of the mother. Finally, the relative contributions of birth dose and infant vaccination in reducing perinatal infections depends on the main sources of infection. The birth dose vaccine is administered within hours of birth and therefore confers protection from HBV during the first few weeks of life. Two or more months have passed before an infant acquires the full protection conferred by the last dose of the infant vaccine series, and the relative contributions of the birth dose and infant vaccines in protecting infants from vertical infection is therefore partly dependent on the period during which infants are at most risk of being vertically infected. Hence, once again, in a data-sparse and complex area of the natural history of HBV, two different assumptions have been made that are both consistent with the data that are available.

As such, this illustrates a good reason for doing model comparisons. The most important, but often most easily overlooked assumptions in the model concern the structure. Whilst a great deal of effort is often expended to show how different parameter values affect model behaviour, and parameters are chosen that maximise ‘within dataset’ fit, it is much harder to show how structure affects things; not least because, the structure encodes the latent prior beliefs of the analyst. Model comparison—of two independent, but well-developed models—overcomes this by confronting two internally-consistent worldviews with one another.

Stepping back, though, it is worth remarking at how similar the models do in fact behave at high infant vaccine series levels. They are completely different in almost every way: they make different assumptions for horizontal infections and for perinatal infections, and infectiousness is categorized differently in the two models (HBeAg status in the Imperial model and viral load in the PRoGReSs model). But, because both models use the data carefully and fit to several different data types, they generate similar results at high infant vaccine series levels, which are recommended by the WHO [9].

### Limitations of this study

This study focuses on two widely used dynamic population-level HBV models: the Imperial HBV model and PRoGReSs. Another widely used HBV model—the Goldstein model [22]—is a static cohort-level model and therefore does not incorporate herd immunity. Consequently, both the inputs and the outputs of the Goldstein model are structurally different from the models considered in the present study, and including the Goldstein model in this comparison would be beyond the scope of the analysis.

Another limitation of this study is that the two models were calibrated on limited HBsAg and HBeAg/HBsAg prevalence data. Calibrating the two models at several time points would reduce the variability in the outputs of each model, allowing more precise comparisons between the two models. However, historical country-specific prevalence data are limited for most countries.

Further limitations of this study are that the models were compared in the absence of treatment (whereas a large scale-up of treatment might subdue the differences noted here) and that the models were compared in terms of only two outcomes (incident chronic HBV infections and total deaths due to HBV). However, treatment coverage is currently low in many settings and incident chronic HBV infections and HBV deaths tend to be of great interest in informing public health policy decisions. Moreover, changes in these two outcomes reflect changes in other model outcomes such as incident acute HBV infections and incident cases and deaths from decompensated cirrhosis and liver cancer caused by HBV. Hence, this study provides a comparison of two currently used HBV models under a range of vaccination coverage levels (but in the absence of prophylactic and treatment interventions) in terms of two important model outcomes that shed light on the main differences between the two models.

## Supporting information

S1 Appendix Document outlining model descriptions and model calibrations. (DOCX)

S1 Fig Imperial hepatitis B model structure. (PDF)

S2 Fig Fit of the Imperial HBV model to prevalence and HBV death rates data in Ethiopia. Confidence bands of the 2.5 and 97.5 percentiles are shown. CDA: Center for Disease Analysis; GBD: Global Burden of Disease; HBV: hepatitis B virus; HBeAg: hepatitis B e antigen; HBsAg: hepatitis B surface antigen.
(PDF)

S3 Fig Fit of the Imperial HBV model to prevalence and HBV death rates data in India. Confidence bands of the 2.5 and 97.5 percentiles are shown. CDA: Center for Disease Analysis; GBD: Global Burden of Disease; HBV: hepatitis B virus; HBeAg: hepatitis B e antigen; HBsAg: hepatitis B surface antigen.
(PDF)

S4 Fig Fit of the Imperial HBV model to prevalence and HBV death rates data in Nigeria. Confidence bands of the 2.5 and 97.5 percentiles are shown. CDA: Center for Disease Analysis; GBD: Global Burden of Disease; HBV: hepatitis B virus; HBeAg: hepatitis B e antigen; HBsAg: hepatitis B surface antigen.
(PDF)

S5 Fig Fit of the Imperial HBV model to prevalence and HBV death rates data in Pakistan. Confidence bands of the 2.5 and 97.5 percentiles are shown. CDA: Center for Disease Analysis; GBD: Global Burden of Disease; HBV: hepatitis B virus; HBeAg: hepatitis B e antigen; HBsAg: hepatitis B surface antigen.
(PDF)

S6 Fig Incident cases of chronic HBV infection, with confidence bands, between 2015 and 2099. (TIF)

S7 Fig HBV-related deaths, with confidence bands, between 2015 and 2099. (TIF)

S8 Fig Percentage reductions in incident chronic HBV infections between scenarios I and II, with fitted regression lines and 95% confidence intervals. ETH: Ethiopia, IND: India, NGA: Nigeria, PAK: Pakistan.
(TIF)

S9 Fig Percentage incident chronic HBV cases in scenarios II and III due to perinatal and horizontal infections. (TIF)

S10 Fig PRoGReSs model structure. (TIF)

S1 Dataset Data used in the analyses. (ZIP)

S1 Text Statistics from the regression analyses for S8 Fig. (TXT)

## References

[1] Roth GA, Abate D, Abate KH, Abay SM, Abbafati C, Abbasi N, et al Global, regional, and national age-sex-specific mortality for 282 causes of death in 195 countries and territories, 1980–2017: a systematic analysis for the Global Burden of Disease Study 2017. The Lancet. 2018;392(10159):1736–88. 10.1016/S0140-6736(18)32203-7 30496103 PMC6227606

[2] Lozano R, Naghavi M, Foreman K, Lim S, Shibuya K, Aboyans V, et al Global and regional mortality from 235 causes of death for 20 age groups in 1990 and 2010: a systematic analysis for the Global Burden of Disease Study 2010. The Lancet. 2012;380(9859):2095–128. Epub 2012/12/19. 10.1016/s0140-6736(12)61728-0 . 23245604 PMC10790329

[3] Stanaway JD, Flaxman AD, Naghavi M, Fitzmaurice C, Vos T, Abubakar I, et al The global burden of viral hepatitis from 1990 to 2013: findings from the Global Burden of Disease Study 2013. The Lancet. 2016;388(10049):1081–8. 10.1016/S0140-6736(16)30579-7 27394647 PMC5100695

[4] Peto TJ, Mendy ME, Lowe Y, Webb EL, Whittle HC, Hall AJ. Efficacy and effectiveness of infant vaccination against chronic hepatitis B in the Gambia Hepatitis Intervention Study (1986–90) and in the nationwide immunisation program. BMC Infectious Diseases. 2014;14 Epub 2014/01/09. 10.1186/1471-2334-14-7 24397793 PMC3898092

[5] Hepatitis B (HepB3) immunization coverage estimates by country [Internet]. World Health Organization 2016 Available from: http://apps.who.int/gho/data/node.main.A828?lang=en.

[6] WHO/UNICEF estimates of national immunisation coverage (WUENIC). Geneva: World Health Organization; 2019.

[7] Gavi progress report 2017. Geneva, Switzerland: Gavi, The Vaccine Alliance, 2017.

[8] Annex C: hepatitis B birth dose investment case. Gavi, The Vaccine Alliance, 2018.

[9] Combating hepatitis B and C to reach elimination by 2030. Geneva, Switzerland: World Health Organization, 2016 May 2016. Report No.

[10] Nayagam S, Thursz M, Sicuri E, Conteh L, Wiktor S, Low-Beer D, et al Requirements for global elimination of hepatitis B: a modelling study. The Lancet Infectious Diseases. 2016;16(12):1399–408. 10.1016/S1473-3099(16)30204-3 . 27638356

[11] Razavi-Shearer D, Gamkrelidze I, Nguyen MH, Chen DS, Van Damme P, Abbas Z, et al Global prevalence, treatment, and prevention of hepatitis B virus infection in 2016: a modelling study. The Lancet Gastroenterology & Hepatology. 2018;3(6):383–403. 10.1016/S2468-1253(18)30056-6 . 29599078

[12] Eaton JW, Johnson LF, Salomon JA, Bärnighausen T, Bendavid E, Bershteyn A, et al HIV treatment as prevention: systematic comparison of mathematical models of the potential impact of antiretroviral therapy on HIV incidence in South Africa. PLOS Medicine. 2012;9(7). 10.1371/journal.pmed.1001245 22802730 PMC3393664

[13] Houben RMGJ, Menzies NA, Sumner T, Huynh GH, Arinaminpathy N, Goldhaber-Fiebert JD, et al Feasibility of achieving the 2025 WHO global tuberculosis targets in South Africa, China, and India: a combined analysis of 11 mathematical models. The Lancet Global Health. 2016;4(11):e806–e15. 10.1016/S2214-109X(16)30199-1 27720688 PMC6375908

[14] Wallace DI, Southworth BS, Shi X, Chipman JW, Githeko AK. A comparison of five malaria transmission models: benchmark tests and implications for disease control. Malaria Journal. 2014;13(1). 10.1186/1475-2875-13-268 25011942 PMC4105118

[15] World Bank country and lending groups [Internet]. The World Bank Group. 2019. Available from: https://datahelpdesk.worldbank.org/knowledgebase/articles/906519-world-bank-country-and-lending-groups.

[16] World Population Prospects: the 2017 revision [Internet]. United Nations, Department of Economic and Social Affairs, Population Division. 2017. Available from: https://population.un.org/wpp/Download/Standard/Population/.

[17] GBD results tool [Internet]. Institute for Health Metrics and Evaluation. 2019 [cited December 2018]. Available from: http://ghdx.healthdata.org/gbd-results-tool.

[18] MATLAB. version 9.2.0.556344 (R2017a). Natick, Massachusetts, United States: The MathWorks Inc.; 2017.

[19] Microsoft Excel. 2019 (16.0) ed. Redmond, Washington, United States: Microsoft Corporation.

[20] Visual Basic for Applications. 7.1 ed. Redmond, Washington, United States: Microsoft Corporation.

[21] Lo KJ, Tsai YT, Lee SD, Yeh CL, Wang JY, Chiang BN, et al Combined passive and active immunization for interruption of perinatal transmission of hepatitis B virus in Taiwan. Hepatogastroenterology. 1985;32(2):65–8. Epub 1985/04/01. . 3159639

[22] Goldstein ST, Zhou F, Hadler SC, Bell BP, Mast EE, Margolis HS. A mathematical model to estimate global hepatitis B disease burden and vaccination impact. International Journal of Epidemiology. 2005;34(6):1329–39. Epub 2005/10/27. 10.1093/ije/dyi206 . 16249217

