# Supplementary material for: Modelling hepatitis B virus infection and impact of timely birth dose vaccine: A comparison of two simulation models
Source: PLoS One. 2020 Aug 10;15(8):e0237525. doi: 10.1371/journal.pone.0237525 (PMC7416941; doi:10.1371/journal.pone.0237525)
Supplement: S1 Appendix — (DOCX) [file pone.0237525.s001.docx]

# Supplementary information

# Imperial HBV model

### Model description

The Imperial hepatitis B (HBV) model [1] is a population-level, deterministic, dynamic transmission model structured by gender and age. It was developed to model the spread of HBV via vertical and horizontal infection within populations in the presence of interventions such as the birth dose vaccine, the infant HBV vaccine series and antiviral treatment. The model, shown in S1 Fig, contains the acute HBV states Severe Acute (S_13_) and Non-severe Acute (S_12_) and the chronic HBV states Immune Tolerant (S_2_), Immune Reactive (S_3_), Asymptomatic Carrier (S_4_), Chronic Hepatitis B (S_5_), Compensated Cirrhosis (S_6_), Decompensated Cirrhosis (S_7_) and Liver Cancer (S_8_). The Immune Tolerant and the Immune Reactive states contain HBsAg+ HBeAg+ individuals, who are assumed to be 15 times more infectious than the HBsAg+ HBeAg- individuals in the other states. The model also contains states for Susceptible (S_1_), Immune (recovered/vaccinated) individuals (S_9_) and tenofovir (TDF) based treatment (S_10_). Individuals in the Immune Reactive, Chronic Hepatitis B, Compensated Cirrhosis and Decompensated Cirrhosis states are considered eligible for treatment. HBV-related deaths (S_11_) can occur from the Severe Acute, Compensated Cirrhosis, Decompensated Cirrhosis and Liver Cancer states. State S_11_ keeps track of individuals that have died due to HBV (as opposed to other causes). Transition rates and model parameters are given in Table A.

**S1 Fig. Imperial hepatitis B model structure.**

**Table A. Model parameters, in which *k* represents sex (1 for female and 2 for male) and *w* represents country.**

| **Parameter** | **Definition** | **Value** | **Source** |
| --- | --- | --- | --- |
| Transition rates | | | |
| *r*_1,w_ | annual transition rate from Immune Tolerant (S_2_) to Immune Reactive (S_3_) | 0.1*κ(a)*** | [2-4] |
| *r*_2,k,w_ | annual transition rate from Immune Tolerant (S_2_) to Liver Cancer (S_8_) | *j(a)** | assumption |
| *r*_3,w_ | annual transition rate from Immune Reactive (S_3_) to Asymptomatic Carrier (S_4_) | 0.05*κ(a)*** | [5, 6] |
| *r*_4_ | annual transition rate from Immune Reactive (S_3_) to Chronic Hepatitis B (S_5_) | 0.005*l(a)**** | assumption |
| *r*_5,k,w_ | annual transition rate from Immune Reactive (S_3_) to Liver Cancer (S_8_) | 2*j(a)** | [7-13] |
| *r*_6_ | annual transition rate from Asymptomatic Carrier (S_4_) to Chronic Hepatitis B (S_5_) | 0.01 | [14-20] |
| *r*_7,k,w_ | annual transition rate from Asymptomatic Carrier (S_4_) to Liver Cancer (S_8_) | 0.5*j(a)** | [21] |
| *r*_8_ | annual transition rate from Asymptomatic Carrier (S_4_) to Immune (S_9_) | 0.01 | [5, 17, 18, 22] |
| *r*_9,k,w_ | annual transition rate from Chronic Hepatitis B (S_5_) to Compensated Cirrhosis (S_6_) | 0.04*m(a)***** | [9, 11, 16, 22-28] |
| *r*_10,k,w_ | annual transition rate from Chronic Hepatitis B (S_5_) to Liver Cancer (S_8_) | 2*j(a)** | [22] |
| *r*_11_ | annual transition rate from Compensated Cirrhosis (S_6_) to Decompensated Cirrhosis (S_7_) | 0.04 | [13, 29-31] |
| *r*_12,k,w_ | annual transition rate from Compensated Cirrhosis (S_6_) to Liver Cancer (S_8_) | 13*j(a)** | [2, 7, 11, 13, 30, 32-40] |
| *r*_13_ | annual transition rate from Compensated Cirrhosis (S_6_) to HBV-related deaths (S_11_) | 0.04 | [41] |
| *r*_14_ | annual transition rate from Decompensated Cirrhosis (S_7_) to Liver Cancer (S_8_) | 0.04 | [29, 32-36, 42] |
| *r*_15_ | annual transition rate from Decompensated Cirrhosis (S_7_) to HBV-related deaths (S_11_) | 0.3 | [41] |
| *r*_16_ | annual transition rate from Liver Cancer (S_8_) to HBV-related deaths (S_11_) | 0.5 | [41] |
| *r*_17_ | annual transition rate from TDF-based treatment (S_10_) to HBV-related deaths (S_11_) | 0.001 | assumption |
| *r*_18_ | annual transition rate from Immune Reactive (S_3_) to Compensated Cirrhosis (S_6_) | 0.028*l(a)**** | [43] |
| *r*_19_ | annual transition rate from Non-severe Acute (S_12_) to Immune Tolerant (S_2_) | 2*σ(a)*† | [44, 45] |
| *r*_20_ | annual transition rate from Non-severe Acute (S_12_) to Immune (S_9_) | 2[1 – *σ(a)*]† | [44, 45] |
| *r*_21_ | annual transition rate from Severe Acute (S_13_) to Immune Tolerant (S_2_) | 1.9*σ(a)*† | [44, 45] |
| *r*_22_ | annual transition rate from Severe Acute (S_13_) to Immune (S_9_) | 1.9[1 – *σ(a)*]† | [44, 45] |
| *r*_23_ | annual transition rate from Severe Acute (S_13_) to HBV-related deaths (S_11_) | 0.1 | [46] |
| *r*_24_ | annual transition rate of vaccinated infants from Susceptible (S_1_) to Immune (S_9_) | pInfant*_w_(t)·* *v_1_* |  |
| *c*_1,w_ | rate of initiation of TDF treatment in Immune Reactive (S_3_) stage | varies with country |  |
| *c*_2,w_ | rate of initiation of TDF treatment in Chronic Hepatitis B (S_5_) stage | varies with country |  |
| *c*_3,w_ | rate of initiation of TDF treatment in Compensated Cirrhosis (S_6_) stage | varies with country |  |
| *c*_4,w_ | rate of initiation of TDF treatment in Decompensated Cirrhosis (S_7_) stage | varies with country |  |
| Demography and coverage data | | | |
| *N_k,w_(a, t)* | population size |  | [47] |
| *f_w_(a, t)* | fertility rates |  | [47] |
| *R_w_(t)* | male-to-female birth ratios |  | [47] |
| *M_k,w_(a, t)* | migration rates |  | [47] |
| *μ_k,w_(a, t)* | death rates |  | [47] |
| pBD*_w_(t)* | birth dose vaccination coverage |  | WUENIC [48] and Gavi, the Vaccine Alliance |
| pInfant*_w_(t)* | infant vaccination coverage |  | WUENIC [48] and Gavi, the Vaccine Alliance |
| Transmission parameters | | | |
| *ε*_2_ | infectiousness multiplier for S_2_ | 15 | assumption |
| *ε*_3_ | infectiousness multiplier for S_3_ | 15 | assumption |
| *ε*_4_ | infectiousness multiplier for S_4_ | 1 | assumption |
| *ε*_5_ | infectiousness multiplier for S_5_ | 1 | assumption |
| *ε*_6_ | infectiousness multiplier for S_6_ | 1 | assumption |
| *ε*_7_ | infectiousness multiplier for S_7_ | 1 | assumption |
| *ε*_8_ | infectiousness multiplier for S_8_ | 1 | assumption |
| *ε*_10_ | infectiousness multiplier for S_10_ | 0 | assumption |
| *ε*_14_ | infectiousness multiplier for S_12_ | 1 | assumption |
| *ε*_15_ | infectiousness multiplier for S_13_ | 1 | assumption |
| β_child_ | transmission among those under 5 years old | fitted |  |
| β_young_ | transmission among those under 15 years old | 0.001 | assumption |
| β_all_ | transmission among those over 5 years old | 0.001 | assumption |
| δ_1_ | risk of mother-to-child transmission (MTCT) if mother is HBsAg positive, HBeAg negative in the absence of intervention | fitted |  |
| δ_2_ | risk of MTCT if mother is HBeAg positive in the absence of intervention | 90% |  |
| δ_3_ | risk of MTCT if mother is virally suppressed due to treatment in the absence of additional intervention | 1% | assumption |
| Intervention efficacy | | | |
| v_1_ | efficacy of infant vaccination against HBV | 95% | [49] |
| v_2_ | efficacy of birth dose vaccination if mother is HBsAg positive, HBeAg negative | 95% | [50] |
| v_3_ | efficacy of birth dose vaccination if mother is HBeAg positive | 83% | [51-54] |
| v_4_ | efficacy of birth dose vaccination if mother is virally suppressed due to treatment | 92.3%–99.4% | assumption |
| v_5_ | efficacy of birth dose vaccination and peripartum antiviral therapy (PPT) if mother is HBeAg positive | 99.4% | assumption |
| v_6_ | efficacy of birth dose vaccination and PPT if mother is virally suppressed due to treatment | 92.3%–99.4% | assumption |

†$\sigma\left( a \right)=\left\{ \begin{aligned} \exp\left( -0.645a^{0.455} \right), 6 \text{months}<a<100 \text{years} \\ 0.885, a\leq6 \text{months} \end{aligned} \right.$

*Definition: $j(a)=\left\{ \begin{aligned} 0,a<10 \text{years} \\ \alpha_{\text{cancer}}\tau_{\text{cancer}}{(a-10)}^{2}, 10 \text{years}\leq a<100 \text{years} \end{aligned} \right.$; α_cancer_ is the cancer factor; τ_cancer_ is the male co-factor

**Definition: $\kappa(a)=uexp(-\vartheta a)$; ϑ is the rate of HBeAg loss; *u* is the HBeAg loss factor

***Definition: $l(a)=\left\{ \begin{aligned} 0, a<20 \text{years} \\ 1, 20 \text{years}\leq a<100 \text{years} \end{aligned} \right.$

****Definition: $m(a)=\left\{ \begin{aligned} 0,a<25 \text{years} \\ \alpha_{\text{cirrhosis}}\tau_{\text{cirrhosis}}{(a-25)}^{2}, 25 \text{years}\leq a<100 \text{years} \end{aligned} \right.$; α_cirrhosis_ is the cirrhosis factor; τ_cirrhosis_ is the male co-factor

Movement between the states in a country *w* is specified by partial differential equations for number of people *X* of sex *k* in terms of age (*a*) and time (*t*):

$$\frac{\partial X_{1,k,w}\left( a,t \right)}{\partial a}+\frac{\partial X_{1,k,w}\left( a,t \right)}{\partial t}=B_{1,k,w}\left( t \right)\cdot I(a=0 \text{months})-X_{1,k,w}(a,t)\left[ \lambda(a,t)+r_{24}\cdot I(a=6 \text{months})+\mu_{k,w}(a,t)+M_{k,w}(a,t) \right]$$

$$\frac{\partial X_{2,k,w}\left( a,t \right)}{\partial a}+\frac{\partial X_{2,k,w}\left( a,t \right)}{\partial t}= {B_{2,k,w}\left( t \right)\cdot I(a=0 \text{months})+r}_{19}X_{12,k,w}(a,t)+{r_{21}X}_{13,k,w}(a,t)- X_{2,k,w}(a,t)\left[ r_{1,w}+r_{2,k,w}+\mu_{k,w}(a,t)+M_{k,w}(a,t) \right]$$

$$\frac{\partial X_{3,k,w}\left( a,t \right)}{\partial a}+\frac{\partial X_{3,k,w}\left( a,t \right)}{\partial t}=r_{1,w}X_{2,k,w}(a,t) - X_{3,k,w}(a,t)\left[ r_{3,w}+r_{4}+r_{5,k,w}+r_{18}+c_{1,w}+\mu_{k,w}(a,t)+M_{k,w}(a,t) \right]$$

$$\frac{\partial X_{4,k,w}\left( a,t \right)}{\partial a}+\frac{\partial X_{4,k,w}\left( a,t \right)}{\partial t}=r_{3,w}X_{3,k,w}(a,t) - X_{4,k,w}(a,t)\left[ r_{6}+r_{7,k,w}+r_{8}+\mu_{k,w}(a,t)+M_{k,w}(a,t) \right]$$

$$\frac{\partial X_{5,k,w}\left( a,t \right)}{\partial a}+\frac{\partial X_{5,k,w}\left( a,t \right)}{\partial t}=r_{4}X_{3,k,w}(a,t)+r_{6}X_{4,k,w}(a,t) - X_{5,k,w}(a,t)\left[ r_{9,k,w}+r_{10,k,w}+c_{2,w}+\mu_{k,w}(a,t)+M_{k,w}(a,t) \right]$$

$$\frac{\partial X_{6,k,w}\left( a,t \right)}{\partial a}+\frac{\partial X_{6,k,w}\left( a,t \right)}{\partial t}={{r_{18}X}_{3,k,w}(a,t)+r}_{9,k,w}X_{5,k,w}(a,t) - X_{6,k,w}(a,t)\left[ r_{11}+r_{12,k,w}+r_{13}+c_{3,w}+\mu_{k,w}(a,t)+M_{k,w}(a,t) \right]$$

$$\frac{\partial X_{7,k,w}\left( a,t \right)}{\partial a}+\frac{\partial X_{7,k,w}\left( a,t \right)}{\partial t}=r_{11}X_{6,k,w}(a,t) - X_{7,k,w}(a,t)\left[ r_{14}+r_{15}+c_{4,w}+\mu_{k,w}(a,t)+M_{k,w}(a,t) \right]$$

$$\frac{\partial X_{8,k,w}\left( a,t \right)}{\partial a}+\frac{\partial X_{8,k,w}\left( a,t \right)}{\partial t}=r_{2,k,w}X_{2,k,w}(a,t)+r_{5,k,w}X_{3,k,w}(a,t)+r_{7,k,w}X_{4,k,w}(a,t)+r_{10,k,w}X_{5,k,w}(a,t)+r_{12,k,w}X_{6,k,w}(a,t)+r_{14}X_{7,k,w}(a,t)- X_{8,k,w}(a,t)\left[ r_{16}+\mu_{k,w}(a,t)+M_{k,w}(a,t) \right]$$

$$\frac{\partial X_{9,k,w}\left( a,t \right)}{\partial a}+\frac{\partial X_{9,k,w}\left( a,t \right)}{\partial t}={r_{24}I(a=6 \text{months})\cdot X}_{1,k,w}(a,t){+{{r_{8}X}_{4,k,w}(a,t)+r}_{20}X}_{12,k,w}(a,t)+ {r_{22}X}_{13,k,w}(a,t)- X_{9,k,w}(a,t)\left[ \mu_{k,w}(a,t) +M_{k,w}(a,t) \right]$$

$$\frac{\partial X_{10,k,w}\left( a,t \right)}{\partial a}+\frac{\partial X_{10,k,w}\left( a,t \right)}{\partial t}=c_{1,w}X_{3,k,w}(a,t)+c_{2,w}X_{5,k,w}(a,t)+c_{3,w}X_{6,k,w}(a,t)+c_{4,w}X_{7,k,w}(a,t)- X_{10,k,w}(a,t)\left[ r_{17}+\mu_{k,w}(a,t)+M_{k,w}(a,t) \right]$$

$$\frac{\partial X_{11,k,w}\left( a,t \right)}{\partial a}+\frac{\partial X_{11,k,w}\left( a,t \right)}{\partial t}=r_{13}X_{6,k,w}(a,t)+r_{15}X_{7,k,w}(a,t)+r_{16}X_{8,k,w}(a,t)+r_{17}X_{10,k,w}(a,t)+r_{23}X_{13,k,w}(a,t)- X_{11,k,w}(a,t) \mu_{k,w}(a,t)$$

$$\frac{\partial X_{12,k,w}\left( a,t \right)}{\partial a}+\frac{\partial X_{12,k,w}\left( a,t \right)}{\partial t}= 0.99\cdot\lambda(a,t)\cdot X_{1,k,w}(a,t)- X_{12,k,w}(a,t) \left[ r_{19}+r_{20}+\mu_{k,w}(a,t)+M_{k,w}(a,t) \right]$$

$$\frac{\partial X_{13,k,w}\left( a,t \right)}{\partial a}+\frac{\partial X_{13,k,w}\left( a,t \right)}{\partial t}=0.01\cdot\lambda(a,t)\cdot X_{1,k,w}(a,t)- X_{13,k,w}(a,t) \left[ r_{21}+r_{22}+r_{23}+\mu_{k,w}(a,t)+M_{k,w}(a,t) \right]$$

In the above partial differential equations, *I(·)* is the indicator function returning 1 if the condition in brackets is true and 0 otherwise. The solutions to the partial differential equations are approximated by means of Euler integration with 0.1 year time steps.

Horizontal transmission is denoted by λ(*a*,*t*) where

$$\lambda(a,t)=\beta_{\text{child}}\cdot I(1\leq a<5 \text{years})\frac{\int_{1}^{5} \sum_{k} \sum_{i\mathcal{\in I}} \varepsilon_{i}X_{i,k,w}(a,t)da}{\int_{1}^{5} \sum_{k} \sum_{i\in\mathcal{A}} X_{i,k,w}(a,t)da}+\beta_{\text{young}}\cdot I(1\leq a<15 \text{years})\frac{\int_{1}^{15} \sum_{k} \sum_{i\mathcal{\in I}} \varepsilon_{i}X_{i,k,w}(a,t)da}{\int_{1}^{15} \sum_{k} \sum_{i\in\mathcal{A}} X_{i,k,w}(a,t)da}+\beta_{\text{all}}\cdot I(a\geq5 \text{years})\frac{\int_{5}^{100} \sum_{k} \sum_{i\in\mathcal{I}} \varepsilon_{i}X_{i,k,w}(a,t)da}{\int_{5}^{100} \sum_{k} \sum_{i\in\mathcal{A}} X_{i,k,w}(a,t)da}$$

where $\mathcal{I}$ consists of the indices of the infectious states

S_2_, S_3_, S_4_, S_5_, S_6_, S_7_, S_8_, S_10_, S_12_ and S_13_

and $\mathcal{A}$ consists of the indices of the population states

S_1_, S_2_, S_3_, S_4_, S_5_, S_6_, S_7_, S_8_, S_9_, S_10_, S_12_ and S_13_.

Mother-to-child transmission (MTCT) is modelled as follows:

Let

$$q_{i}\in\left\{ \delta_{1},\delta_{2},\delta_{3} \right\}$$

be the probability of MTCT in state *S_i_* in the absence of intervention based on the mother’s HBV status (δ_1_: mother is HBsAg positive, HBeAg negative, δ_2_: mother is HBeAg positive and δ_3_: mother is virally suppressed due to treatment),

$$\text{pBD}_{w}(t)$$

be the birth dose coverage in the country *w*,

$$\text{pBDPPT}_{i,w}(t)$$

be the coverage of peripartum antiviral treatment (PPT) in state *S_i_*,

$$\text{eBD}_{i}$$

be the efficacy of birth dose vaccination in reducing the probability of MTCT in state *S_i_* based on the mother’s HBV status (v_2_: mother is HBsAg positive, HBeAg negative, v_3_: mother is HBeAg positive and v_4_: mother is virally suppressed due to treatment), and

$$\text{eBDPPT}_{i}$$

be the efficacy of birth dose vaccination and PPT in reducing the probability of MTCT in state *S_i_* (containing *X_i_* people) based on the mother’s HBV status (v_5_: mother is HBeAg positive and v_6_: mother is virally suppressed due to treatment). Then number of chronic births is calculated as

$$B_{2,k,w}(t)=\sigma(0)\cdot\left[ R_{w}(t)\cdot I(k=2)+\left[ 1-R_{w}(t) \right]\cdot I(k=1) \right]\cdot\sum_{i} \left[ \left( \left[ 1-\text{pBD}_{w}(t) \right]+\text{pBD}_{w}(t)\cdot\left[ 1-\text{pBDPPT}_{i,w}(t) \right]\cdot\left[ 1-\text{eBD}_{i} \right]+\text{pBD}_{w}(t)\cdot\text{pBDPPT}_{i,w}(t)\cdot\left[ 1-\text{eBDPPT}_{i} \right] \right)\cdot\int f_{w}(a,t)\cdot X_{i,1,w}(a,t) da \right]\cdot q_{i}$$

and number of uninfected births as

$$B_{1,k,w}\left( t \right)=\int\sum_{i} f_{w}\left( a,t \right)\cdot X_{i,1,w}\left( a,t \right)da-B_{2,k,w}(t).$$

Subsequent to the last publication [1], the states

- S_12_: Non-severe Acute, and
- S_13_: Severe Acute

were added to model acute HBV infection (S1 Fig). The percentage of newly infected individuals sent to Severe Acute was set to 1% [55], while the remaining 99% of infected individuals are sent to Non-severe Acute. The transition rate from Severe Acute HBV directly to HBV-related death was set to 0.05 / (6 months) [46]. Individuals spend an average of 6 months in the acute part of the model [4]. The transition rates directly from Immune Reactive (S_3_) to Chronic Hep B (S_5_) and from Immune Reactive to Compensated Cirrhosis (S_6_) were made 0/year for 0–20 year olds to improve the fit of the model to the reference death rates data (see next section). The transition rate from Chronic Hep B to Compensated Cirrhosis was changed to gender-dependent rates that are 0/year for 0–25 year olds and a parabolic function that increases with age for 25–99 year olds to improve the fit of the model to the reference cirrhosis death rates data. To improve the fit to the reference cancer death rates data, the functional form for the risk of developing cancer was changed to a gender-dependent compound function that is 0/year for 0–10 year olds and a parabolic function that increases with age for 10–99 year olds.

### Model calibration

The Imperial HBV model is calibrated to country-level, age-specific HBsAg positive prevalence data from one or two time points and country-level (HBeAg positive)/(HBsAg positive) prevalence data in pregnant women from a single time point, which were obtained from the Polaris Observatory. In addition, country-level, age and gender-specific liver cancer and cirrhosis death rates (deaths/100,000 individuals) due to HBV were obtained from the Global Burden of Disease (GBD) Results Tool website [5] for the years 1990 and 2005. For HBsAg prevalence, the ages are partitioned into 0–9 year olds and 10–99 year olds, since prevalence in younger age groups tends to be of more interest than in older age groups. For cirrhosis and cancer death rates, the ages are partitioned into 5–39 year olds and 40–99 year olds, since death rates are much lower below the age of 40 than above the age of 40. The death rates data are also compared separately for males and females. A weighted mean absolute percentage error objective function is used, with the fit of the model to younger age groups in the reference data considered more important than the fit to older age groups, which tends to be much more uncertain and at risk of bias. The most important terms are given a weighting of 4 (the younger age groups for HBsAg and cirrhosis and cancer death rates comparisons between the model and reference value), the less important terms a weighting of 2 (the older age groups in the HBsAg and death rates comparisons), and the least important term a weighting of 1 (HBeAg/HBsAg prevalence in pregnant women comparison). The seven model parameters, which are calibrated independently for each country, are listed in Table B.

**Table B. Model parameters calibrated to country-level prevalence and death rates data.**

| Parameter | Description | Range |
| --- | --- | --- |
| β_child_ | risk of horizontal transmission to susceptible 1 to 4 year olds | [0, 9 x 10^-1^] |
| δ_1_ | risk of vertical transmission from HBsAg positive, HBeAg negative mothers to their infants at birth without intervention | [5 x 10^-2^, 9 x 10^-1^] |
| ϑ | rate at which seroconversion from HBsAg+ HBeAg+ to HBsAg+ HBeAg- declines with age | [0, 2.0001] |
| α_cancer_ | rate at which liver cancer risk increases with age | [1 x 10^-5^, 1 x 10^-2^] |
| α_cirrhosis_ | rate at which cirrhosis risk increases with age | [1 x 10^-3^, 1] |
| τ_cancer_ | ratio of males to females developing liver cancer | [1 x 10^-2^, 50] |
| τ_cirrhosis_ | ratio of males to females developing cirrhosis | [1 x 10^-2^, 50] |

HBeAg: hepatitis B e antigen; HBsAg: hepatitis B surface antigen.

Calibrations are performed using the Approximate Bayesian Computation (ABC) Sequential Monte Carlo (SMC) algorithm [56]. Uniform prior distributions are used for the seven parameters, with the ranges given in Table B. The objective function is used as the distance function for calculating the error between the perturbed model outputs of proposed particles and the reference data. Successive populations each contain 100 particles, and tolerances of successive populations are chosen as the median of the errors for the 100 particles in the previous population of particles. Noise is added to particles sampled from the previous population of particles using a multivariate normal distribution as the perturbation kernel, with zero mean and a covariance matrix calculated from the previous population of particles. Noise is added to the model outcomes of proposed particles as follows:

- HBsAg prevalent cases are modelled with a binomial(*n*,*p*) distribution, with *n* the size of the population of interest and *p* the HBsAg prevalence in the same population,
- HBeAg prevalent cases in HBsAg positive pregnant women are modelled with a binomial(*n*,*p*) distribution, with *n* the number of HBsAg positive prevalent cases in the population of interest and *p* the HBeAg prevalence amongst HBsAg positive individuals in the same population,
- number of deaths from cirrhosis and liver cancer are each modelled with a Poisson(*λ*) distribution, with *λ* the number of deaths in the population of interest.

Perturbed particles that result in model outcomes that, when perturbed, result in errors below the current tolerance level are accepted. The calibration analyses are run until a population of particles is formed that has a tolerance level below 0.25. This value was chosen, since the tolerance drops this far relatively quickly but decreases further at a much slower pace. Fits for Ethiopia, India, Nigeria and Pakistan are shown in S2–S5 Figs.

**S2 Fig. Fit of the Imperial HBV model to prevalence and HBV death rates data in Ethiopia.** Confidence bands of the 2.5 and 97.5 percentiles are shown. CDA: Center for Disease Analysis; GBD: Global Burden of Disease; HBV: hepatitis B virus; HBeAg: hepatitis B e antigen; HBsAg: hepatitis B surface antigen.

**S3 Fig. Fit of the Imperial HBV model to prevalence and HBV death rates data in India.** Confidence bands of the 2.5 and 97.5 percentiles are shown. CDA: Center for Disease Analysis; GBD: Global Burden of Disease; HBV: hepatitis B virus; HBeAg: hepatitis B e antigen; HBsAg: hepatitis B surface antigen.

**S4 Fig. Fit of the Imperial HBV model to prevalence and HBV death rates data in Nigeria.** Confidence bands of the 2.5 and 97.5 percentiles are shown. CDA: Center for Disease Analysis; GBD: Global Burden of Disease; HBV: hepatitis B virus; HBeAg: hepatitis B e antigen; HBsAg: hepatitis B surface antigen.

**S5 Fig. Fit of the Imperial HBV model to prevalence and HBV death rates data in Pakistan.** Confidence bands of the 2.5 and 97.5 percentiles are shown. CDA: Center for Disease Analysis; GBD: Global Burden of Disease; HBV: hepatitis B virus; HBeAg: hepatitis B e antigen; HBsAg: hepatitis B surface antigen.

## PRoGReSs

The model structure of PRoGReSs is provided in S10 Fig. Since the last publication of the PRoGReSs model [57], the progression rates listed in Table C have been updated.

**S10 Fig. PRoGReSs model structure.**

**Table C. Annual disease progression rates of HBV infection in the PRoGReSs model (%).**

Annual progression rates, male

| Age group | 0–  4 | 5–  9 | 10–14 | 15–19 | 20–24 | 25–29 | 30–34 | 35–39 | 40–44 | 45–49 | 50–54 | 55–59 | 60–64 | 65–69 | 70–74 | 75–79 | 80–84 | 85+ |
| --- | --- | --- | --- | --- | --- | --- | --- | --- | --- | --- | --- | --- | --- | --- | --- | --- | --- | --- |
| CHB to Cirr, LVL | 0.0 | 0.0 | 0.0 | 0.0 | 0.0 | 0.0 | 0.0 | 0.1 | 0.3 | 0.6 | 0.8 | 0.8 | 0.9 | 1.0 | 1.6 | 2.0 | 2.4 | 2.8 |
| Low | 0.0 | 0.0 | 0.0 | 0.0 | 0.0 | 0.0 | 0.0 | 0.0 | 0.2 | 0.5 | 0.5 | 0.6 | 0.6 | 0.6 | 0.9 | 1.7 | 1.8 | 1.9 |
| High | 0.0 | 0.0 | 0.0 | 0.0 | 0.0 | 0.0 | 0.1 | 0.2 | 0.5 | 0.8 | 0.9 | 0.9 | 0.9 | 1.4 | 2.1 | 2.4 | 2.7 | 3.0 |
| CHB to Cirr, HVL | 0.0 | 0.1 | 0.1 | 0.2 | 0.3 | 0.4 | 0.7 | 1.4 | 1.7 | 2.4 | 4.1 | 4.5 | 4.9 | 6.1 | 7.8 | 8.5 | 9.1 | 9.8 |
| Low | 0.0 | 0.0 | 0.0 | 0.1 | 0.1 | 0.1 | 0.2 | 0.8 | 1.2 | 1.4 | 2.2 | 2.4 | 2.7 | 3.3 | 4.5 | 4.9 | 5.3 | 5.7 |
| High | 0.1 | 0.1 | 0.1 | 0.2 | 0.4 | 0.7 | 1.1 | 1.5 | 2.0 | 3.2 | 4.4 | 5.7 | 6.8 | 10.3 | 14.5 | 17.5 | 20.4 | 23.4 |
| CHB to HCC, LVL | 0.0 | 0.0 | 0.0 | 0.0 | 0.0 | 0.0 | 0.0 | 0.0 | 0.1 | 0.1 | 0.2 | 0.3 | 0.3 | 0.3 | 0.3 | 0.3 | 0.4 | 0.4 |
| Low | 0.0 | 0.0 | 0.0 | 0.0 | 0.0 | 0.0 | 0.0 | 0.0 | 0.0 | 0.0 | 0.0 | 0.1 | 0.3 | 0.3 | 0.3 | 0.3 | 0.3 | 0.3 |
| High | 0.1 | 0.1 | 0.1 | 0.1 | 0.1 | 0.1 | 0.1 | 0.1 | 0.1 | 0.2 | 0.3 | 0.5 | 0.5 | 0.6 | 0.6 | 0.7 | 0.7 | 0.8 |
| CHB to HCC, HVL | 0.0 | 0.0 | 0.0 | 0.1 | 0.1 | 0.1 | 0.1 | 0.1 | 0.2 | 0.4 | 0.6 | 0.7 | 0.8 | 0.8 | 0.8 | 0.9 | 0.9 | 1.0 |
| Low | 0.0 | 0.0 | 0.0 | 0.0 | 0.1 | 0.1 | 0.1 | 0.1 | 0.1 | 0.1 | 0.3 | 0.2 | 0.7 | 0.5 | 0.5 | 0.5 | 0.5 | 0.5 |
| High | 0.2 | 0.2 | 0.2 | 0.2 | 0.2 | 0.2 | 0.2 | 0.3 | 0.5 | 0.8 | 1.0 | 1.3 | 1.6 | 1.7 | 1.9 | 2.1 | 2.3 | 2.5 |
| Cirr to HCC, LVL | 0.0 | 0.0 | 0.0 | 0.0 | 0.1 | 0.2 | 0.4 | 0.8 | 1.3 | 1.8 | 2.1 | 2.2 | 2.3 | 2.4 | 2.5 | 2.6 | 2.7 | 2.8 |
| Low | 0.0 | 0.0 | 0.0 | 0.0 | 0.1 | 0.1 | 0.1 | 0.1 | 1.2 | 1.2 | 1.1 | 2.1 | 1.7 | 1.7 | 1.7 | 1.7 | 1.7 | 1.7 |
| High | 1.0 | 1.0 | 1.0 | 1.0 | 1.0 | 1.0 | 1.9 | 2.5 | 3.1 | 3.7 | 4.3 | 4.7 | 5.2 | 5.7 | 6.3 | 6.9 | 7.6 | 8.4 |
| Cirr to HCC, HVL | 0.1 | 0.1 | 0.2 | 0.3 | 0.4 | 0.6 | 0.8 | 1.5 | 2.2 | 3.8 | 6.9 | 9.6 | 10.7 | 11.1 | 11.4 | 11.8 | 12.1 | 12.5 |
| Low | 0.0 | 0.0 | 0.0 | 0.0 | 0.4 | 0.4 | 0.4 | 1.0 | 1.5 | 1.1 | 1.0 | 9.5 | 8.9 | 8.9 | 8.9 | 1.0 | 1.0 | 1.0 |
| High | 1.4 | 1.4 | 1.4 | 1.4 | 1.4 | 1.5 | 2.0 | 3.0 | 4.0 | 4.8 | 6.9 | 12.7 | 13.2 | 14.5 | 15.9 | 17.5 | 19.3 | 21.2 |
| DCC to LRD | 24.0 | 24.0 | 24.0 | 24.0 | 24.0 | 24.0 | 24.0 | 24.0 | 24.0 | 24.0 | 24.0 | 24.0 | 24.0 | 24.0 | 24.0 | 24.0 | 24.0 | 24.0 |
| Low |  |  |  |  |  |  |  |  |  |  |  |  |  |  |  |  |  |  |
| High |  |  |  |  |  |  |  |  |  |  |  |  |  |  |  |  |  |  |
| HCC to LRD, sub ys | 21.0 | 21.0 | 21.0 | 21.0 | 21.0 | 21.0 | 21.0 | 21.0 | 21.0 | 21.0 | 21.0 | 21.0 | 21.0 | 21.0 | 21.0 | 21.0 | 21.0 | 21.0 |
| Low |  |  |  |  |  |  |  |  |  |  |  |  |  |  |  |  |  |  |
| High |  |  |  |  |  |  |  |  |  |  |  |  |  |  |  |  |  |  |

Annual progression rates, female

| Age group | 0–  4 | 5–  9 | 10–14 | 15–19 | 20–24 | 25–29 | 30–34 | 35–39 | 40–44 | 45–49 | 50–54 | 55–59 | 60–64 | 65–69 | 70–74 | 75–79 | 80–84 | 85+ |
| --- | --- | --- | --- | --- | --- | --- | --- | --- | --- | --- | --- | --- | --- | --- | --- | --- | --- | --- |
| CHB to Cirr, LVL | 0.0 | 0.0 | 0.0 | 0.0 | 0.0 | 0.0 | 0.0 | 0.1 | 0.1 | 0.3 | 0.3 | 0.3 | 0.3 | 0.4 | 0.6 | 0.8 | 1.0 | 1.1 |
| Low | 0.0 | 0.0 | 0.0 | 0.0 | 0.0 | 0.0 | 0.0 | 0.0 | 0.1 | 0.2 | 0.2 | 0.2 | 0.2 | 0.2 | 0.4 | 0.7 | 0.7 | 0.8 |
| High | 0.0 | 0.0 | 0.0 | 0.0 | 0.0 | 0.0 | 0.0 | 0.1 | 0.2 | 0.3 | 0.3 | 0.4 | 0.4 | 0.5 | 0.8 | 1.0 | 1.1 | 1.2 |
| CHB to Cirr, HVL | 0.0 | 0.0 | 0.0 | 0.1 | 0.1 | 0.2 | 0.3 | 0.5 | 0.7 | 1.0 | 1.6 | 1.8 | 2.0 | 2.4 | 3.1 | 3.4 | 3.7 | 3.9 |
| Low | 0.0 | 0.0 | 0.0 | 0.0 | 0.0 | 0.1 | 0.1 | 0.3 | 0.5 | 0.5 | 0.9 | 1.0 | 1.1 | 1.3 | 1.8 | 2.0 | 2.1 | 2.3 |
| High | 0.0 | 0.0 | 0.1 | 0.1 | 0.2 | 0.3 | 0.5 | 0.6 | 0.8 | 1.3 | 1.8 | 2.3 | 2.7 | 4.1 | 5.8 | 7.0 | 8.2 | 9.4 |
| CHB to HCC, LVL | 0.0 | 0.0 | 0.0 | 0.0 | 0.0 | 0.0 | 0.0 | 0.0 | 0.0 | 0.0 | 0.1 | 0.1 | 0.1 | 0.1 | 0.1 | 0.1 | 0.1 | 0.1 |
| Low | 0.0 | 0.0 | 0.0 | 0.0 | 0.0 | 0.0 | 0.0 | 0.0 | 0.0 | 0.0 | 0.0 | 0.1 | 0.1 | 0.1 | 0.1 | 0.1 | 0.1 | 0.1 |
| High | 0.0 | 0.0 | 0.0 | 0.0 | 0.0 | 0.0 | 0.0 | 0.1 | 0.1 | 0.1 | 0.1 | 0.1 | 0.1 | 0.1 | 0.2 | 0.2 | 0.2 | 0.2 |
| CHB to HCC, HVL | 0.0 | 0.0 | 0.0 | 0.0 | 0.0 | 0.0 | 0.0 | 0.0 | 0.2 | 0.4 | 0.5 | 0.6 | 0.6 | 0.6 | 0.7 | 0.7 | 0.7 | 0.7 |
| Low | 0.0 | 0.0 | 0.0 | 0.0 | 0.0 | 0.0 | 0.0 | 0.0 | 0.1 | 0.1 | 0.2 | 0.2 | 0.6 | 0.5 | 0.5 | 0.5 | 0.5 | 0.5 |
| High | 0.0 | 0.0 | 0.0 | 0.0 | 0.0 | 0.0 | 0.1 | 0.2 | 0.5 | 0.8 | 1.0 | 1.3 | 1.5 | 1.6 | 1.8 | 2.0 | 2.2 | 2.4 |
| Cirr to HCC, LVL | 0.0 | 0.0 | 0.0 | 0.0 | 0.0 | 0.0 | 0.0 | 0.0 | 0.1 | 0.2 | 0.6 | 1.2 | 1.5 | 1.6 | 1.7 | 1.7 | 1.8 | 1.8 |
| Low | 0.0 | 0.0 | 0.0 | 0.0 | 0.0 | 0.0 | 0.0 | 0.0 | 0.0 | 0.0 | 0.5 | 1.0 | 1.5 | 1.5 | 1.6 | 1.6 | 1.6 | 1.6 |
| High | 0.0 | 0.0 | 0.0 | 0.0 | 0.0 | 0.1 | 0.1 | 0.2 | 0.4 | 0.8 | 1.7 | 3.4 | 3.7 | 4.1 | 4.5 | 4.9 | 5.4 | 5.9 |
| Cirr to HCC, HVL | 0.0 | 0.0 | 0.0 | 0.0 | 0.0 | 0.1 | 0.1 | 0.2 | 0.5 | 0.9 | 2.5 | 5.0 | 6.4 | 6.8 | 7.0 | 7.2 | 7.4 | 7.7 |
| Low | 0.0 | 0.0 | 0.0 | 0.0 | 0.0 | 0.0 | 0.0 | 0.0 | 0.0 | 0.0 | 1.2 | 4.5 | 4.5 | 4.5 | 4.5 | 1.0 | 1.0 | 1.0 |
| High | 0.8 | 0.8 | 0.8 | 0.8 | 0.8 | 0.9 | 1.2 | 1.7 | 2.3 | 2.8 | 4.0 | 10.0 | 11.0 | 12.1 | 13.3 | 14.6 | 16.1 | 17.7 |
| DCC to LRD | 24.0 | 24.0 | 24.0 | 24.0 | 24.0 | 24.0 | 24.0 | 24.0 | 24.0 | 24.0 | 24.0 | 24.0 | 24.0 | 24.0 | 24.0 | 24.0 | 24.0 | 24.0 |
| Low |  |  |  |  |  |  |  |  |  |  |  |  |  |  |  |  |  |  |
| High |  |  |  |  |  |  |  |  |  |  |  |  |  |  |  |  |  |  |
| HCC to LRD, sub ys | 21.0 | 21.0 | 21.0 | 21.0 | 21.0 | 21.0 | 21.0 | 21.0 | 21.0 | 21.0 | 21.0 | 21.0 | 21.0 | 21.0 | 21.0 | 21.0 | 21.0 | 21.0 |
| Low |  |  |  |  |  |  |  |  |  |  |  |  |  |  |  |  |  |  |
| High |  |  |  |  |  |  |  |  |  |  |  |  |  |  |  |  |  |  |

HBV — hepatitis B virus; CHB — chronic hepatitis B; Cirr — compensated cirrhosis; LVL — low-viral load; HVL — high-viral load; HCC — hepatocellular carcinoma; DCC — decompensated cirrhosis; LRD — liver-related death; sub ys — after first year.

## Additional analyses from the main article

**Table D. Mother-to-child transmission rates in the Imperial model in the absence of interventions such as treatment and HBIG [1].**

| Vaccination status of infant | Serological status of mother | |
| --- | --- | --- |
|  | HBsAg positive, HBeAg positive | HBsAg positive, HBeAg negative |
| no vaccination | δ_2_ = 90% | δ_1_ (fitted for each country) |
| birth dose vaccination | δ_2_·(100% – v_3_) = 15.3% | δ_1_·(100% - v_2_) = δ_1_·5% |

HBeAg: hepatitis B e antigen; HBsAg: hepatitis B surface antigen.

**Table E. Mother-to-child transmission rates in the PRoGReSs model in the absence of interventions such as treatment and HBIG [57].**

| Vaccination status of infant | Serological status of mother | |
| --- | --- | --- |
|  | HBsAg positive with high viral load | HBsAg positive with low viral load |
| no vaccination | 100.0% | 0% |
| birth dose vaccination only | 90.0% | 0% |
| infant HBV vaccine series with birth dose | 13.8% | 0% |
| infant HBV vaccine series without birth dose | 32.7% | 0% |

HBsAg: hepatitis B surface antigen.

## References

1. Nayagam S, Thursz M, Sicuri E, Conteh L, Wiktor S, Low-Beer D, et al. Requirements for global elimination of hepatitis B: a modelling study. The Lancet Infectious Diseases. 2016;16(12):1399-408. doi: 10.1016/S1473-3099(16)30204-3. PubMed PMID: 27638356.

2. Fattovich G, Bortolotti F, Donato F. Natural history of chronic hepatitis B: special emphasis on disease progression and prognostic factors. Journal of Hepatology. 2008;48(2):335-52. doi: 10.1016/j.jhep.2007.11.011.

3. Chang M-H. Natural history of hepatitis B virus infection in children. Journal of Gastroenterology and Hepatology. 2000;15(s2):E16-E9. doi: 10.1046/j.1440-1746.2000.02096.x.

4. Fattovich G, Brollo L, Giustina G, Noventa F, Pontisso P, Alberti A, et al. Natural history and prognostic factors for chronic hepatitis type B. Gut. 1991;32(3):294. doi: 10.1136/gut.32.3.294.

5. Chen C-J, Yang H-I. Natural history of chronic hepatitis B REVEALed. Journal of Gastroenterology and Hepatology. 2011;26(4):628-38. doi: 10.1111/j.1440-1746.2011.06695.x.

6. Tseng T-C, Liu C-J, Chen C-L, Wang C-C, Su T-H, Kuo SF-T, et al. Serum Hepatitis B Virus-DNA Levels Correlate With Long-term Adverse Outcomes in Spontaneous Hepatitis B e Antigen Seroconverters. The Journal of Infectious Diseases. 2012;205(1):54-63. doi: 10.1093/infdis/jir687.

7. Beasley RP, Lin C-C, Hwang L-Y, Chien C-S. Hepatocellular Carcinoma and Hepatitis B Virus: A Prospective Study of 22 707 Men in Taiwan. The Lancet. 1981;318(8256):1129-33. doi: <https://doi.org/10.1016/S0140-6736(81)90585-7>.

8. Beasley RP. Hepatitis B virus. The major etiology of hepatocellular carcinoma. Cancer. 1988;61(10):1942-56. doi: 10.1002/1097-0142(19880515)61:10<1942::AID-CNCR2820611003>3.0.CO;2-J.

9. Ikeda K, Saitoh S, Suzuki Y, Kobayashi M, Tsubota A, Koida I, et al. Disease progression and hepatocellular carcinogenesis in patients with chronic viral hepatitis: a prospective observation of 2215 patients. Journal of Hepatology. 1998;28(6):930-8. doi: 10.1016/S0168-8278(98)80339-5.

10. Wu G, Zhou W, Zhao Y, Guo S, Wang Z, Zou S, et al. [Study on the natural history of chronic hepatitis B]. Chinese Journal of Hepatology. 2002;10(1):46-8. Epub 2002/02/22. PubMed PMID: 11856503.

11. Lo KJ, Tong MJ, Chien MC, Tsai YT, Liaw YF, Yang KC, et al. The Natural Course of Hepatitis B Surface Antigen—Positive Chronic Active Hepatitis in Taiwan. The Journal of Infectious Diseases. 1982;146(2):205-10. doi: 10.1093/infdis/146.2.205.

12. Liaw Y-F, Tai D-I, Chu C-M, Lin D-Y, Sheen IS, Chen T-J, et al. Early detection of hepatocellular carcinoma in patients with chronic type B hepatitis: A prospective study. Gastroenterology. 1986;90(2):263-7. doi: <https://doi.org/10.1016/0016-5085(86)90919-4>.

13. Xu B, Hu D-C, Rosenberg DM, Jiang Q-W, Lin X-M, Lu J-L, et al. Chronic hepatitis B: A long-term retrospective cohort study of disease progression in Shanghai, China. Journal of Gastroenterology and Hepatology. 2003;18(12):1345-52. doi: 10.1046/j.1440-1746.2003.03187.x.

14. Tseng T-C, Liu C-J, Chen C-L, Wang C-C, Su T-H, Kuo SF-T, et al. Serum hepatitis B virus-DNA levels correlate with long-term adverse outcomes in spontaneous hepatitis B e-antigen seroconverters. The Journal of Infectious Diseases. 2012;205(1):54-63. doi: 10.1093/infdis/jir687.

15. Chu C-M, Liaw Y-F. Incidence and Risk Factors of Progression to Cirrhosis in Inactive Carriers of Hepatitis B Virus. The American Journal of Gastroenterology. 2009;104:1693-9. doi: 10.1038/ajg.2009.187.

16. Chu C-M, Hung S-J, Lin J, Tai D-I, Liaw Y-F. Natural history of hepatitis B e antigen to antibody seroconversion in patients with normal serum aminotransferase levels. The American Journal of Medicine. 2004;116(12):829-34. doi: <https://doi.org/10.1016/j.amjmed.2003.12.040>.

17. Chu C-M, Liaw Y-F. HBsAg seroclearance in asymptomatic carriers of high endemic areas: Appreciably high rates during a long-term follow-up. Hepatology. 2007;45(5):1187-92. doi: 10.1002/hep.21612.

18. Gigi E, Lalla T, Orphanou E, Sinakos E, Vrettou E, Raptopoulou-Gigi M. Long term follow-up of a large cohort of inactive HBsAg (+) / HBeAg (-) / anti-HBe (+) carriers in Greece. Journal of Gastrointestinal and Liver Diseases. 2007;16 1:19-22.

19. Chu C-M, Liaw Y-F. Genotype C hepatitis B virus infection is associated with a higher risk of reactivation of hepatitis B and progression to cirrhosis than genotype B: A longitudinal study of hepatitis B e antigen-positive patients with normal aminotransferase levels at baseline. Journal of Hepatology. 2005;43(3):411-7. doi: 10.1016/j.jhep.2005.03.018.

20. Papatheodoridis GV, Chrysanthos N, Hadziyannis E, Cholongitas E, Manesis EK. Longitudinal changes in serum HBV DNA levels and predictors of progression during the natural course of HBeAg-negative chronic hepatitis B virus infection. Journal of Viral Hepatitis. 2008;15(6):434-41. doi: 10.1111/j.1365-2893.2007.00957.x.

21. Chen JD, Yang HI, Iloeje UH, You SL, Lu SN, Wang LY, et al. Carriers of Inactive Hepatitis B Virus Are Still at Risk for Hepatocellular Carcinoma and Liver-Related Death. Gastroenterology. 2010;138(5):1747-54.e1. doi: 10.1053/j.gastro.2010.01.042.

22. Zacharakis GH, Koskinas J, Kotsiou S, Papoutselis M, Tzara F, Vafeiadis N, et al. Natural history of chronic HBV infection: A cohort study with up to 12 years follow-up in North Greece (part of the Interreg I-II/EC-project). Journal of Medical Virology. 2005;77(2):173-9. doi: 10.1002/jmv.20434.

23. Wu G, Zhou W, Zhao Y, Guo S, Wang Z, Zou S, et al. [Study on the natural history of chronic hepatitis B]. Zhonghua Gan Zang Bing Za Zhi. 2002;10(1):46-8. Epub 2002/02/22. PubMed PMID: 11856503.

24. Hsu Y-S, Chien R-N, Yeh C-T, Sheen IS, Chiou H-Y, Chu C-M, et al. Long-term outcome after spontaneous HBeAg seroconversion in patients with chronic hepatitis B. Hepatology. 2002;35(6):1522-7. doi: 10.1053/jhep.2002.33638.

25. Liaw Y-F, Tai D-I, Chu C-M, Chen T-J. The development of cirrhosis in patients with chronic type B hepatitis: A prospective study. Hepatology. 1988;8(3):493-6. doi: 10.1002/hep.1840080310.

26. Chen T-J, Liaw Y-F. The prognostic significance of bridging hepatic necrosis in chronic type B hepatitis: a histopathologic study. Liver. 1988;8(1):10-6. doi: 10.1111/j.1600-0676.1988.tb00960.x.

27. Lin H-H, Liaw Y-F, Chen T-J, Chu C-M, Huang M-J. Natural course of patients with chronic type B hepatitis following acute hepatitis delta virus superinfection. Liver. 1989;9(3):129-34. doi: 10.1111/j.1600-0676.1989.tb00389.x.

28. Huo T, Wu JC, Hwang SJ, Lai CR, Lee PC, Tsay SH, et al. Factors predictive of liver cirrhosis in patients with chronic hepatitis B: a multivariate analysis in a longitudinal study. European Journal of Gastroenterology and Hepatology. 2000;12(6):687-93. Epub 2000/07/27. doi: 10.1097/00042737-200012060-00019. PubMed PMID: 10912490.

29. Fattovich G, Pantalena M, Zagni I, Realdi G, Schalm SW, Christensen E. Effect of hepatitis B and C virus infections on the natural history of compensated cirrhosis: a cohort study of 297 patients. American Journal of Gastroenterology. 2002;97(11):2886-95. Epub 2002/11/12. doi: 10.1111/j.1572-0241.2002.07057.x. PubMed PMID: 12425564.

30. Liaw YF, Lin DY, Chen TJ, Chu CM. Natural course after the development of cirrhosis in patients with chronic type B hepatitis: a prospective study. Liver. 1989;9(4):235-41. Epub 1989/08/01. PubMed PMID: 2770436.

31. Liaw YF, Sung JJ, Chow WC, Farrell G, Lee CZ, Yuen H, et al. Lamivudine for patients with chronic hepatitis B and advanced liver disease. New England Journal of Medicine. 2004;351(15):1521-31. Epub 2004/10/08. doi: 10.1056/NEJMoa033364. PubMed PMID: 15470215.

32. Tong MJ, Hsien C, Song JJ, Kao JH, Sun HE, Hsu L, et al. Factors associated with progression to hepatocellular carcinoma and to death from liver complications in patients with HBsAg-positive cirrhosis. Dig Dis Sci. 2009;54(6):1337-46. Epub 2009/02/27. doi: 10.1007/s10620-009-0747-y. PubMed PMID: 19242792.

33. Tong MJ, Hsien C, Hsu L, Sun HE, Blatt LM. Treatment recommendations for chronic hepatitis B: an evaluation of current guidelines based on a natural history study in the United States. Hepatology. 2008;48(4):1070-8. Epub 2008/08/09. doi: 10.1002/hep.22476. PubMed PMID: 18688879.

34. Chen YC, Chu CM, Yeh CT, Liaw YF. Natural course following the onset of cirrhosis in patients with chronic hepatitis B: a long-term follow-up study. Hepatology International. 2007;1(1):267-73. Epub 2007/03/01. doi: 10.1007/s12072-007-5001-0. PubMed PMID: 19669348; PubMed Central PMCID: PMCPMC2720722.

35. Kobayashi M, Ikeda K, Hosaka T, Sezaki H, Someya T, Akuta N, et al. Natural history of compensated cirrhosis in the Child-Pugh class A compared between 490 patients with hepatitis C and 167 with B virus infections. Journal of Medical Virology. 2006;78(4):459-65. Epub 2006/02/17. doi: 10.1002/jmv.20562. PubMed PMID: 16482557.

36. Mahmood S, Niiyama G, Kamei A, Izumi A, Nakata K, Ikeda H, et al. Influence of viral load and genotype in the progression of Hepatitis B-associated liver cirrhosis to hepatocellular carcinoma. Liver Int. 2005;25(2):220-5. Epub 2005/03/23. doi: 10.1111/j.1478-3231.2005.01077.x. PubMed PMID: 15780042.

37. Tsai JF, Jeng JE, Ho MS, Chang WY, Hsieh MY, Lin ZY, et al. Effect of hepatitis C and B virus infection on risk of hepatocellular carcinoma: a prospective study. Br J Cancer. 1997;76(7):968-74. Epub 1997/01/01. doi: 10.1038/bjc.1997.493. PubMed PMID: 9328161; PubMed Central PMCID: PMCPMC2228256.

38. Chen D-S. Hepatitis B and C Virus Infections in Hepatocellular Carcinoma and Their Prevention. In: Nishioka K, Suzuki H, Mishiro S, Oda T, editors. Viral Hepatitis and Liver Disease: Springer; 1994. p. 685-9.

39. Ikeda K, Saitoh S, Koida I, Arase Y, Tsubota A, Chayama K, et al. A multivariate analysis of risk factors for hepatocellular carcinogenesis: a prospective observation of 795 patients with viral and alcoholic cirrhosis. Hepatology. 1993;18(1):47-53. Epub 1993/07/01. PubMed PMID: 7686879.

40. Obata H, Hayashi N, Motoike Y, Hisamitsu T, Okuda H, Kobayashi S, et al. A prospective study on the development of hepatocellular carcinoma from liver cirrhosis with persistent hepatitis B virus infection. Int J Cancer. 1980;25(6):741-7. Epub 1980/06/15. doi: 10.1002/ijc.2910250609. PubMed PMID: 14768703.

41. Lin X, Robinson NJ, Thursz M, Rosenberg DM, Weild A, Pimenta JM, et al. Chronic hepatitis B virus infection in the Asia-Pacific region and Africa: review of disease progression. Journal of Gastroenterology and Hepatology. 2005;20(6):833-43. Epub 2005/06/11. doi: 10.1111/j.1440-1746.2005.03813.x. PubMed PMID: 15946129.

42. Hui AY, Chan HL, Leung NW, Hung LC, Chan FK, Sung JJ. Survival and prognostic indicators in patients with hepatitis B virus-related cirrhosis after onset of hepatic decompensation. Journal of Clinical Gastroenterology. 2002;34(5):569-72. Epub 2002/04/18. doi: 10.1097/00004836-200205000-00018. PubMed PMID: 11960072.

43. Lin S-M, Yu M-L, Lee C-M, Chien R-N, Sheen IS, Chu C-M, et al. Interferon therapy in HBeAg positive chronic hepatitis reduces progression to cirrhosis and hepatocellular carcinoma. Journal of Hepatology. 2007;46(1):45-52. doi: 10.1016/j.jhep.2006.08.021.

44. Edmunds WJ, Medley GF, Nokes DJ, Hall AJ, Whittle HC. The influence of age on the development of the hepatitis B carrier state. Proceedings of the Royal Society of London Series B: Biological Sciences. 1993;253(1337):197-201. doi: 10.1098/rspb.1993.0102.

45. Hepatitis B. World Health Organization, 2002.

46. Ichai P, Samuel D. Etiology and prognosis of fulminant hepatitis in adults. Liver Transplantation. 2008;14 Suppl 2:S67-79. Epub 2008/10/01. doi: 10.1002/lt.21612. PubMed PMID: 18825677.

47. World Population Prospects: The 2017 Revision [Internet]. 2017. Available from: <https://esa.un.org/unpd/wpp>.

48. WHO/UNICEF estimates of national immunisation coverage (WUENIC). In: Organization WH, editor. Geneva2019.

49. Peto T, Mendy M, Lowe Y, Webb E, Whittle H, Hall A. Efficacy and effectiveness of infant vaccination against chronic hepatitis B in the Gambia Hepatitis Intervention Study (1986-90) and in the nationwide immunisation program. BMC Infectious Diseases. 2014;14(1):7.

50. Chen HL, Lin LH, Hu FC, Lee JT, Lin WT, Yang YJ, et al. Effects of maternal screening and universal immunization to prevent mother-to-infant transmission of HBV. Gastroenterology. 2012;142(4):773-81.e2. Epub 2011/12/27. doi: 10.1053/j.gastro.2011.12.035. PubMed PMID: 22198276.

51. Xu ZY, Liu CB, Francis DP, Purcell RH, Gun ZL, Duan SC, et al. Prevention of perinatal acquisition of hepatitis B virus carriage using vaccine: preliminary report of a randomized, double-blind placebo-controlled and comparative trial. Pediatrics. 1985;76(5):713-8. Epub 1985/11/01. PubMed PMID: 3903646.

52. Beasley RP, Trepo C, Stevens CE, Szmuness W. The e antigen and vertical transmission of hepatitis B surface antigen. American Journal of Epidemiology. 1977;105(2):94-8. Epub 1977/02/01. doi: 10.1093/oxfordjournals.aje.a112370. PubMed PMID: 835566.

53. Wong VC, Ip HM, Reesink HW, Lelie PN, Reerink-Brongers EE, Yeung CY, et al. Prevention of the HBsAg carrier state in newborn infants of mothers who are chronic carriers of HBsAg and HBeAg by administration of hepatitis-B vaccine and hepatitis-B immunoglobulin. Double-blind randomised placebo-controlled study. Lancet. 1984;1(8383):921-6. Epub 1984/04/28. doi: 10.1016/s0140-6736(84)92388-2. PubMed PMID: 6143868.

54. Lee C, Gong Y, Brok J, Boxall EH, Gluud C. Effect of hepatitis B immunisation in newborn infants of mothers positive for hepatitis B surface antigen: systematic review and meta-analysis. The BMJ. 2006;332(7537):328-36. Epub 2006/01/31. doi: 10.1136/bmj.38719.435833.7C. PubMed PMID: 16443611; PubMed Central PMCID: PMCPMC1363909.

55. Robinson WS. Hepatitis B virus and hepatitis D virus. In: Mandel G, Bennett J, Dolin R, editors. Principles and Practice of Infectious diseases. New York, USA: Churchill Livingstone; 1995. p. 1406-39.

56. Toni T, Welch D, Strelkowa N, Ipsen A, Stumpf MP. Approximate Bayesian computation scheme for parameter inference and model selection in dynamical systems. J R Soc Interface. 2009;6(31):187-202. Epub 2009/02/11. doi: 10.1098/rsif.2008.0172. PubMed PMID: 19205079; PubMed Central PMCID: PMCPMC2658655.

57. Razavi-Shearer D, Gamkrelidze I, Nguyen MH, Chen DS, Van Damme P, Abbas Z, et al. Global prevalence, treatment, and prevention of hepatitis B virus infection in 2016: a modelling study. The Lancet Gastroenterology & Hepatology. 2018;3(6):383-403. doi: 10.1016/S2468-1253(18)30056-6. PubMed PMID: 29599078.
