# Supplementary figures and images for: Modelling hepatitis B virus infection and impact of timely birth dose vaccine: A comparison of two simulation models
Source: PLoS One. 2020 Aug 10;15(8):e0237525. doi: 10.1371/journal.pone.0237525 (PMC7416941; doi:10.1371/journal.pone.0237525)

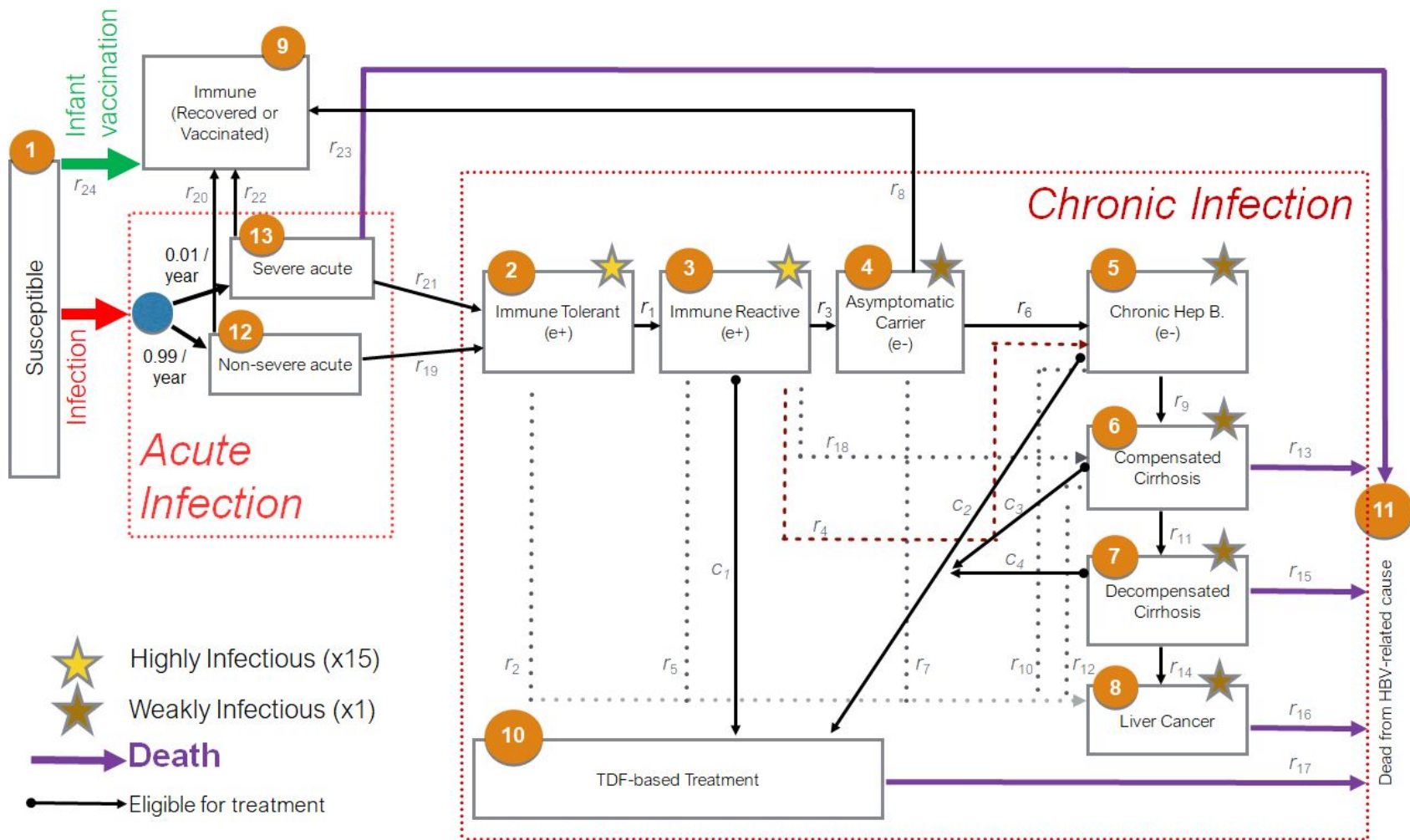

Supplement: S1 Fig — (PDF) [file pone.0237525.s002.pdf]

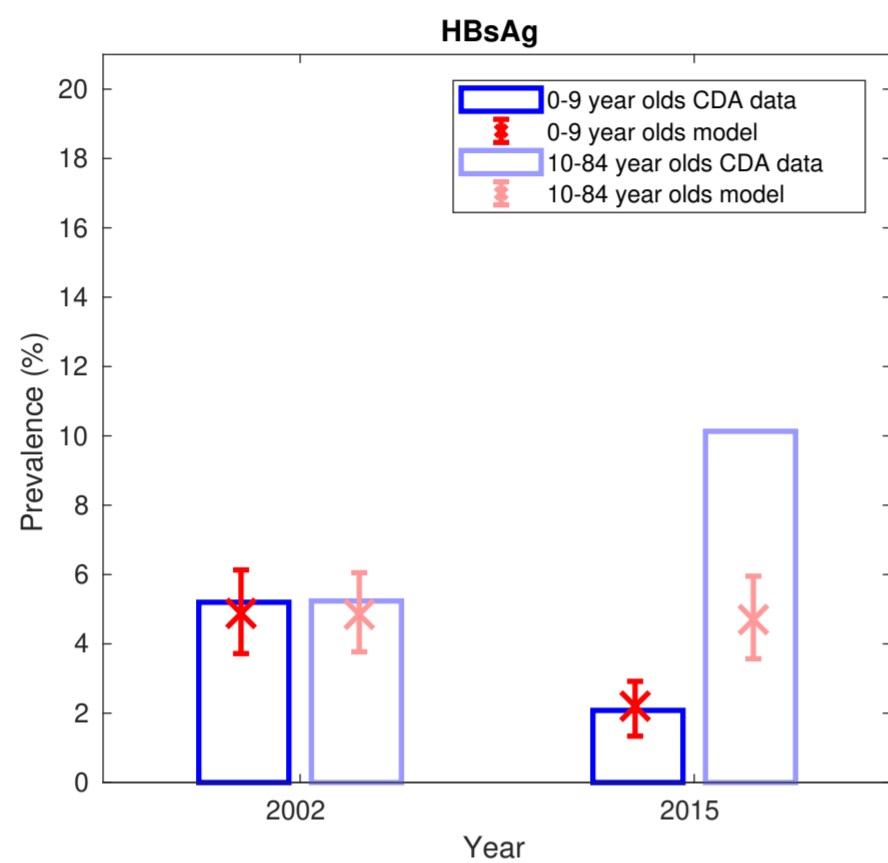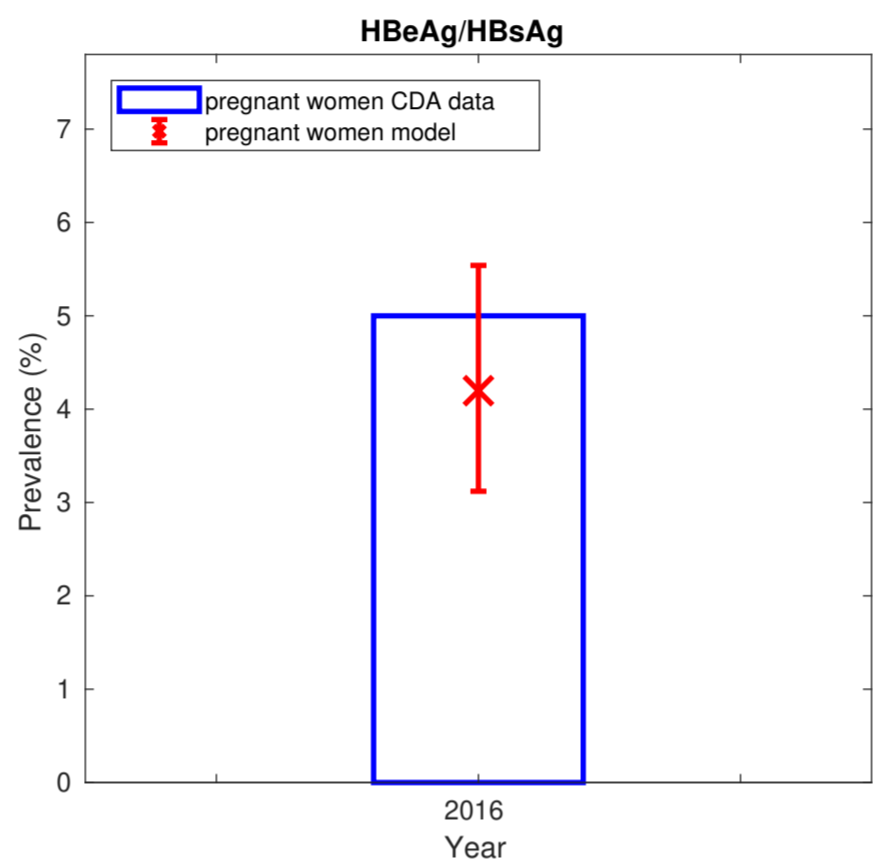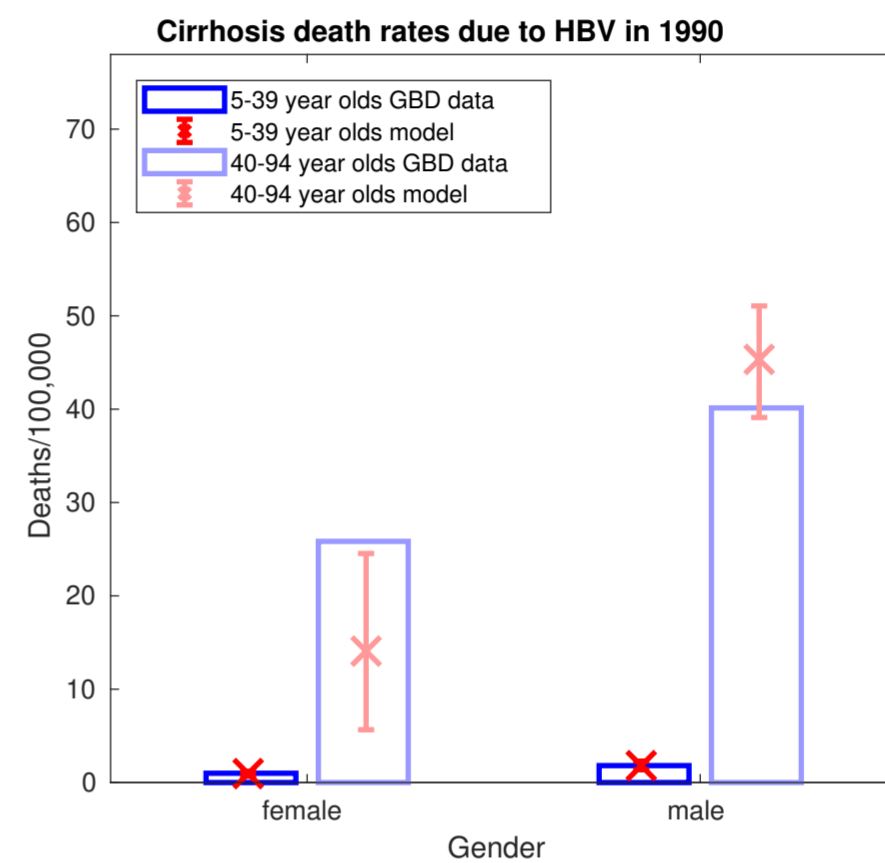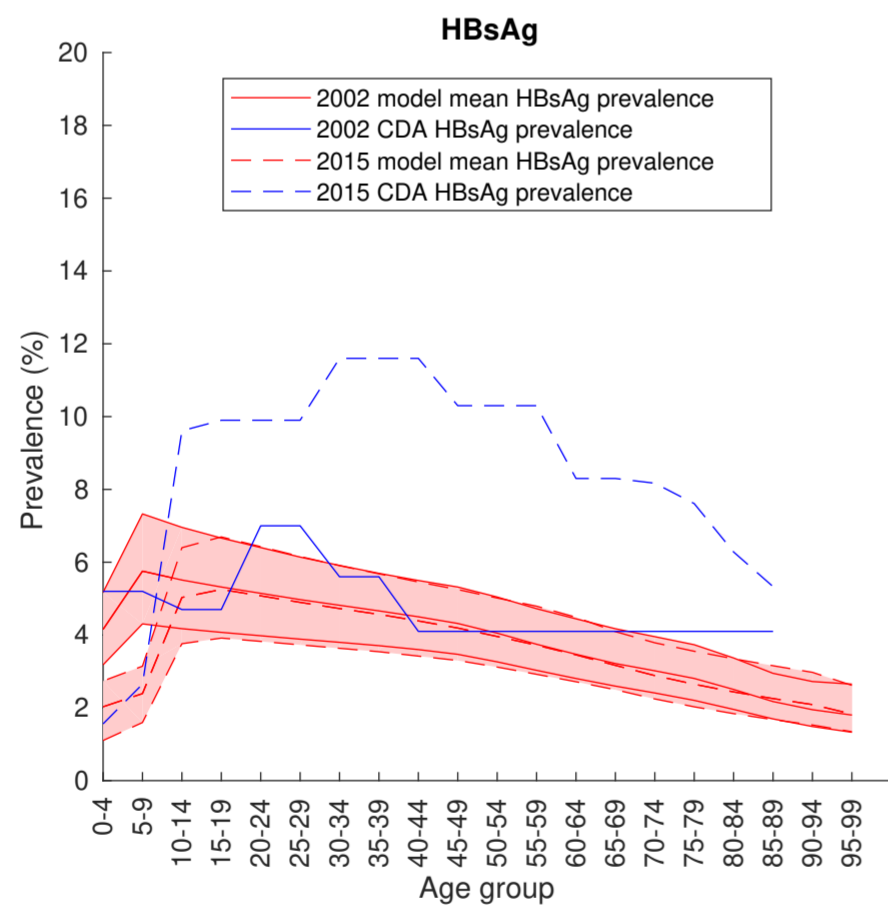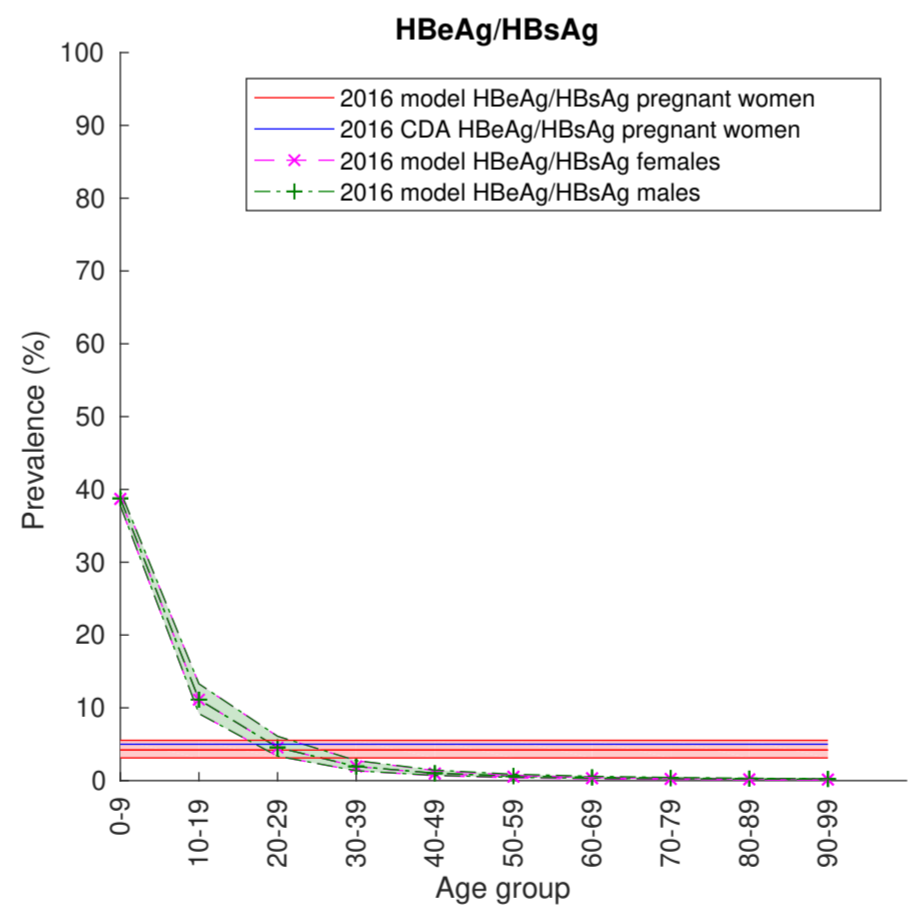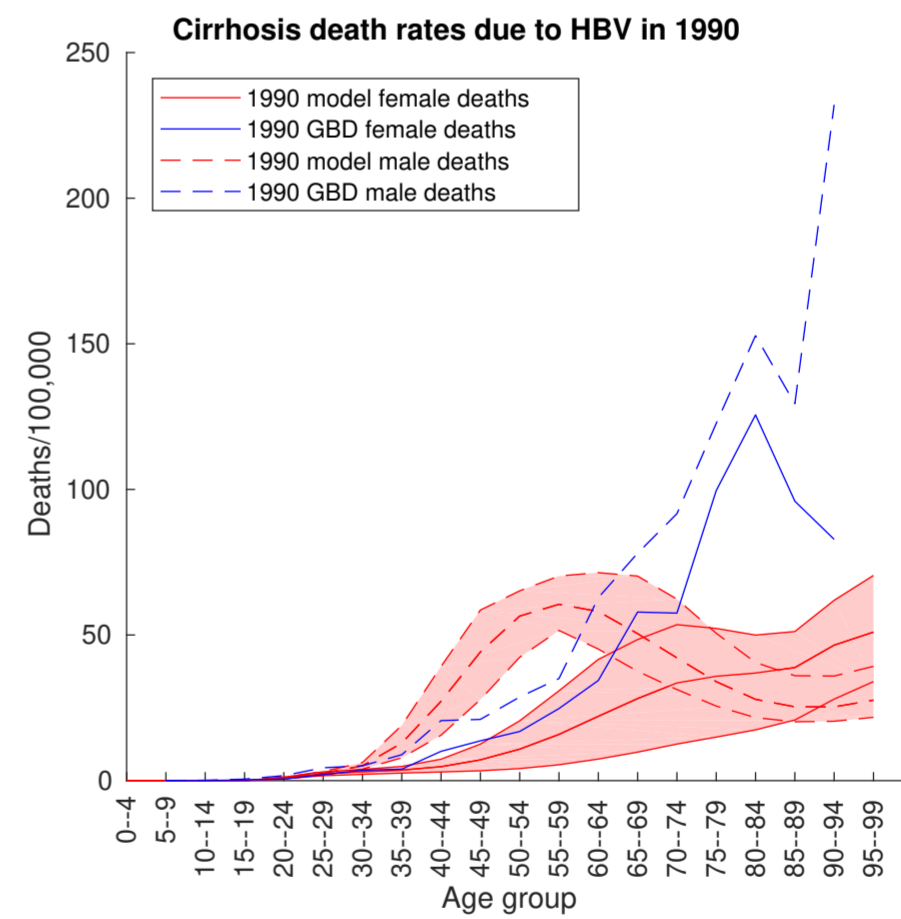

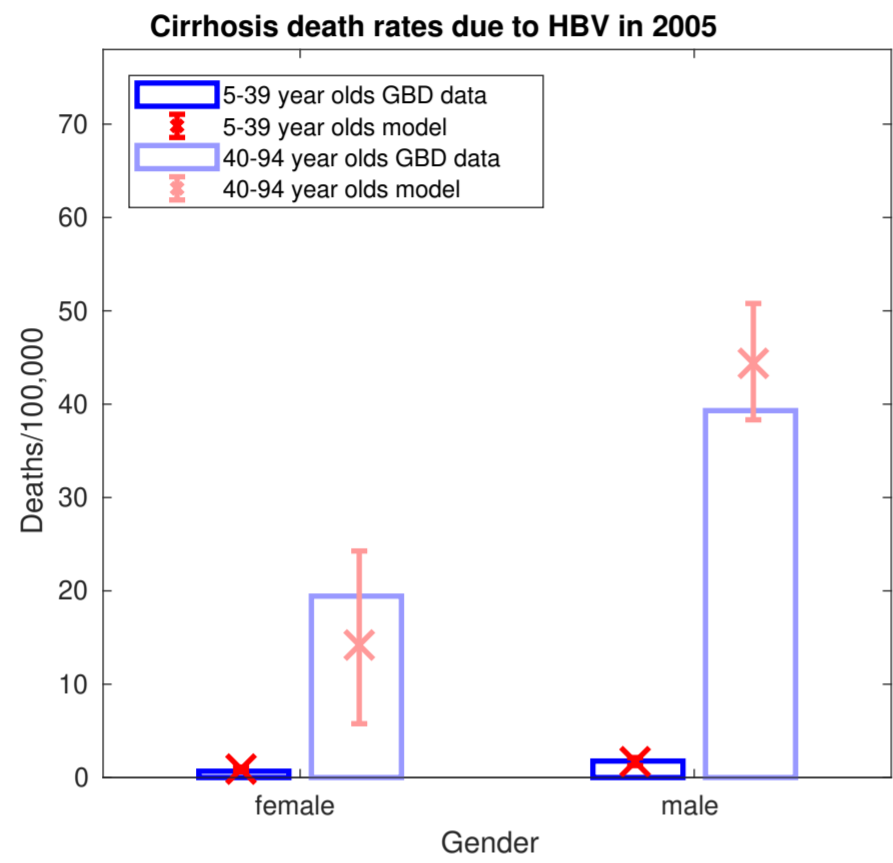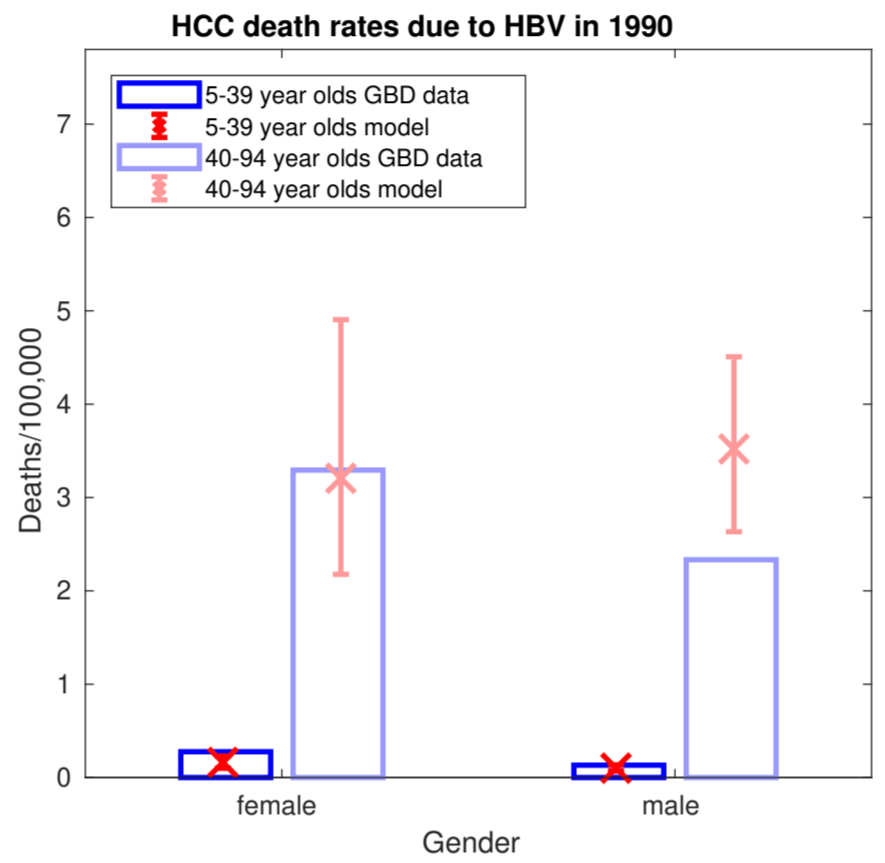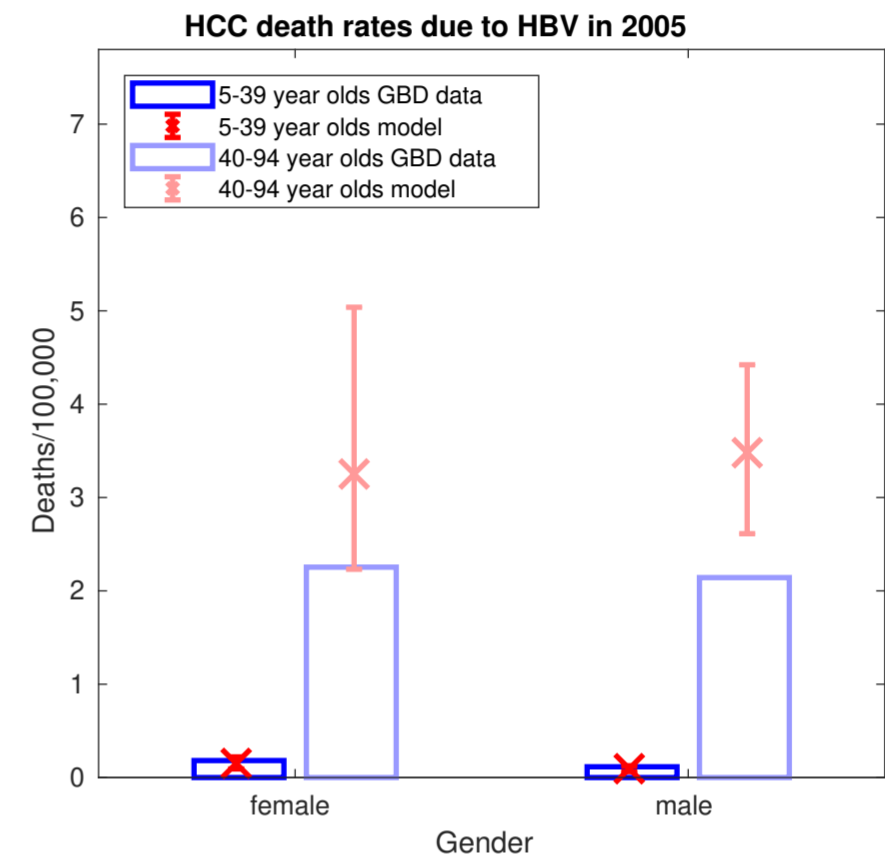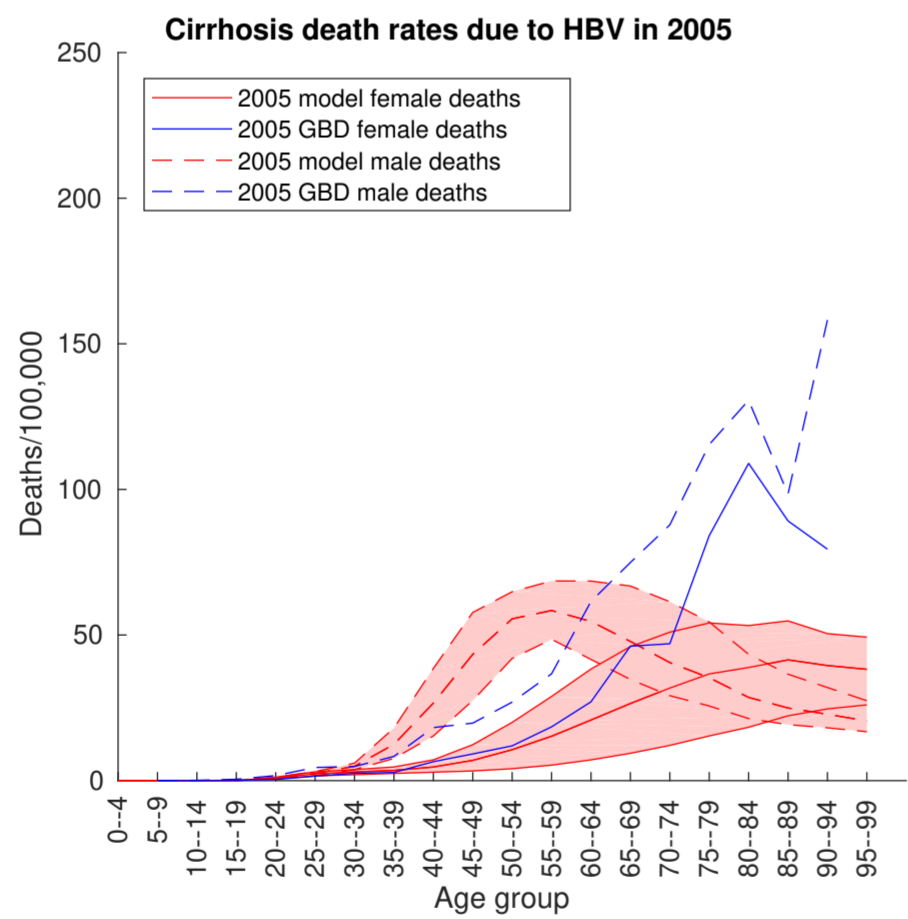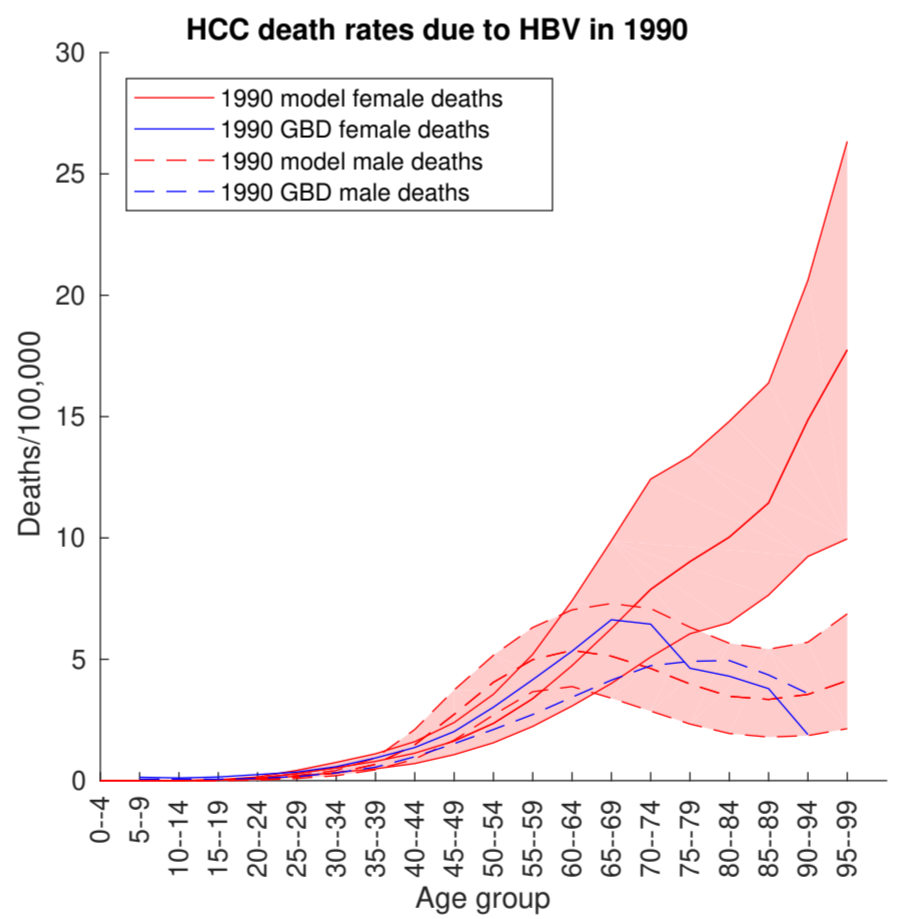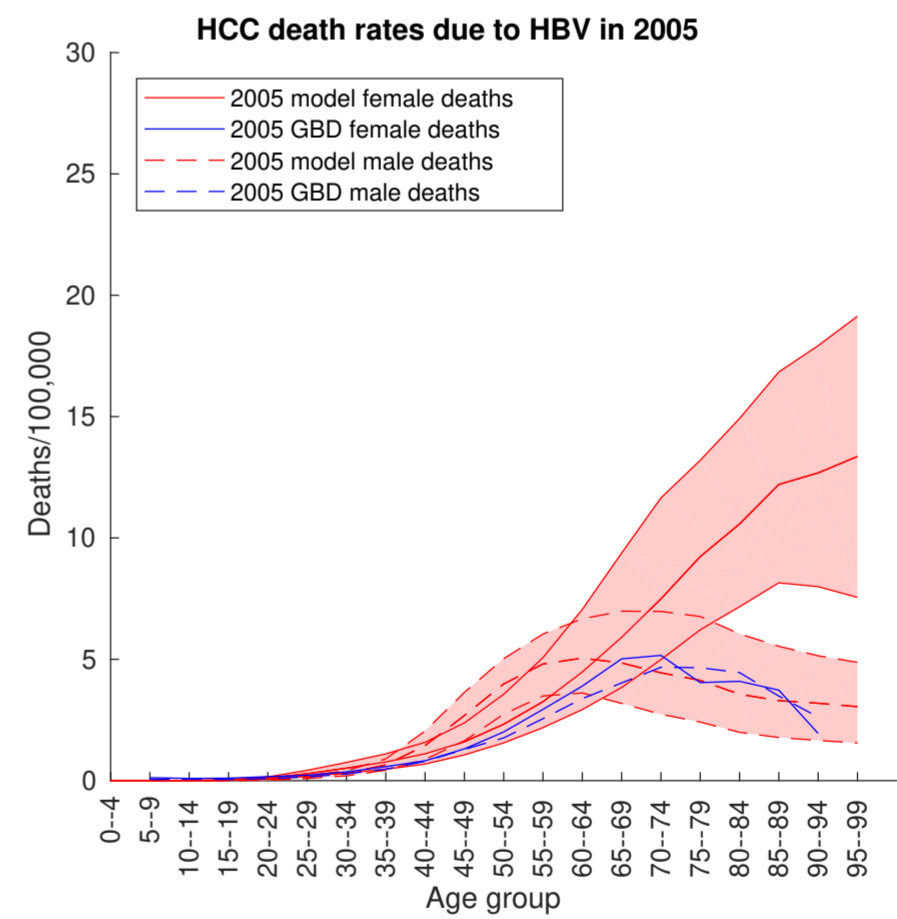

Supplement: S2 Fig — Confidence bands of the 2.5 and 97.5 percentiles are shown. CDA: Center for Disease Analysis; GBD: Global Burden of Disease; HBV: hepatitis B virus; HBeAg: hepatitis B e antigen; HBsAg: hepatitis B surface antigen. (PDF) [file pone.0237525.s003.pdf]

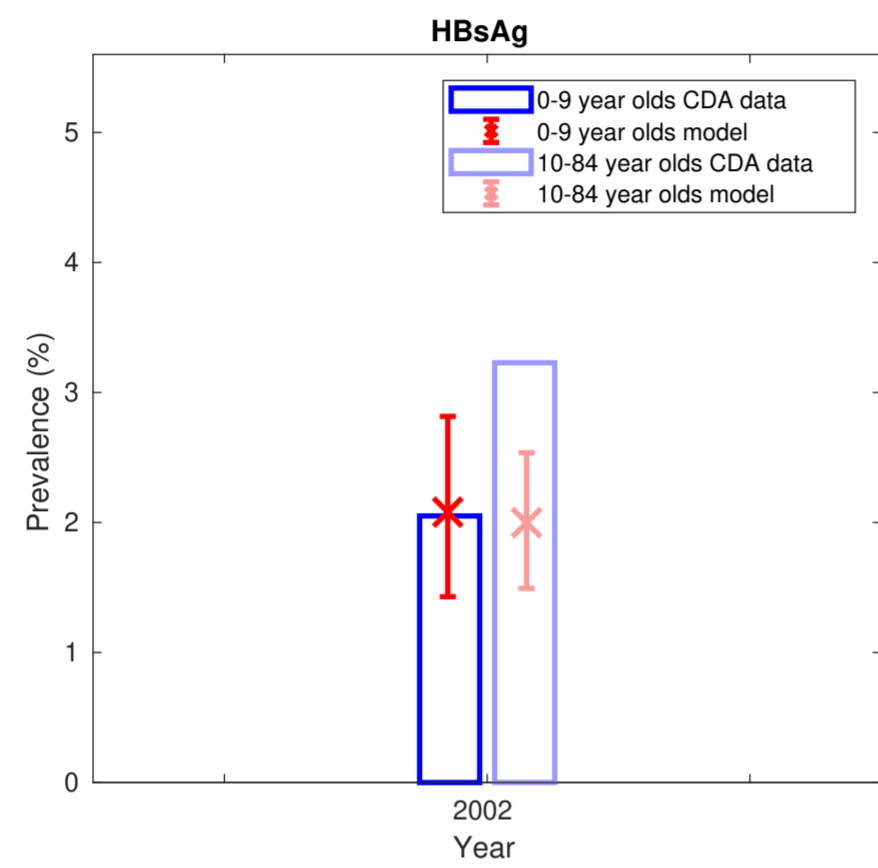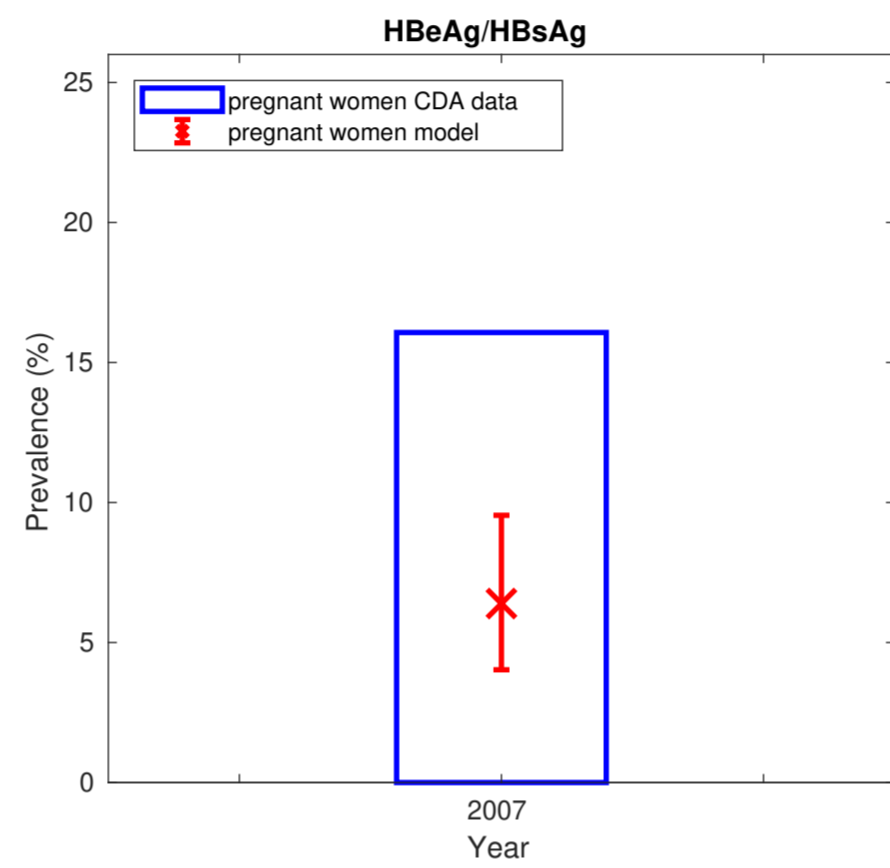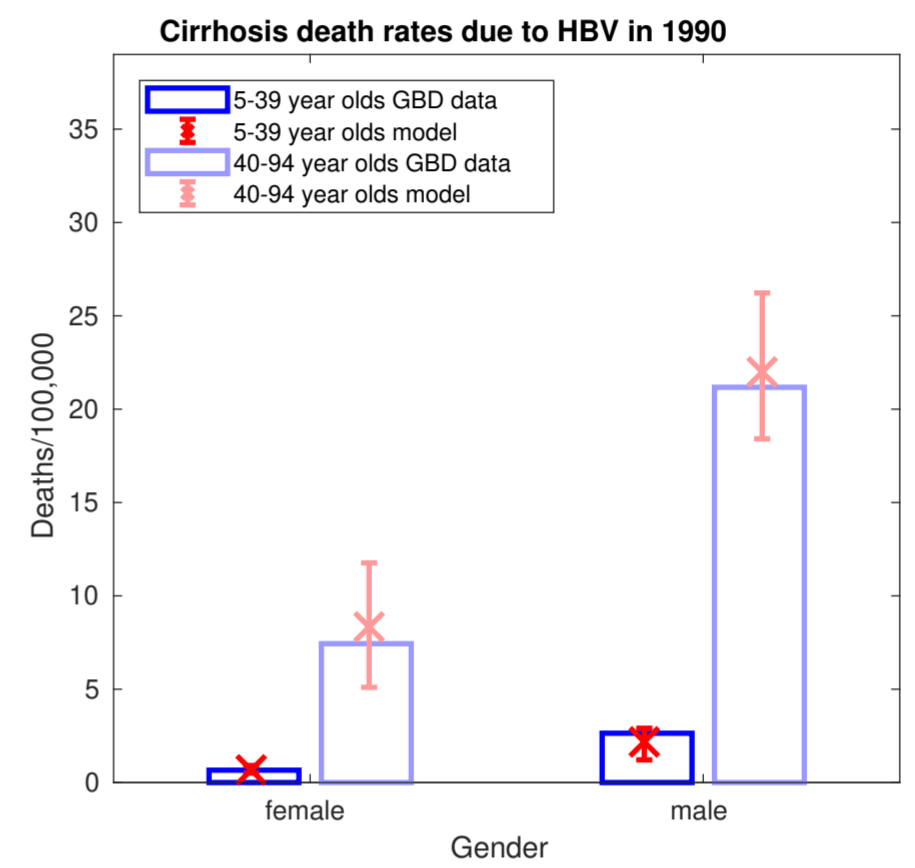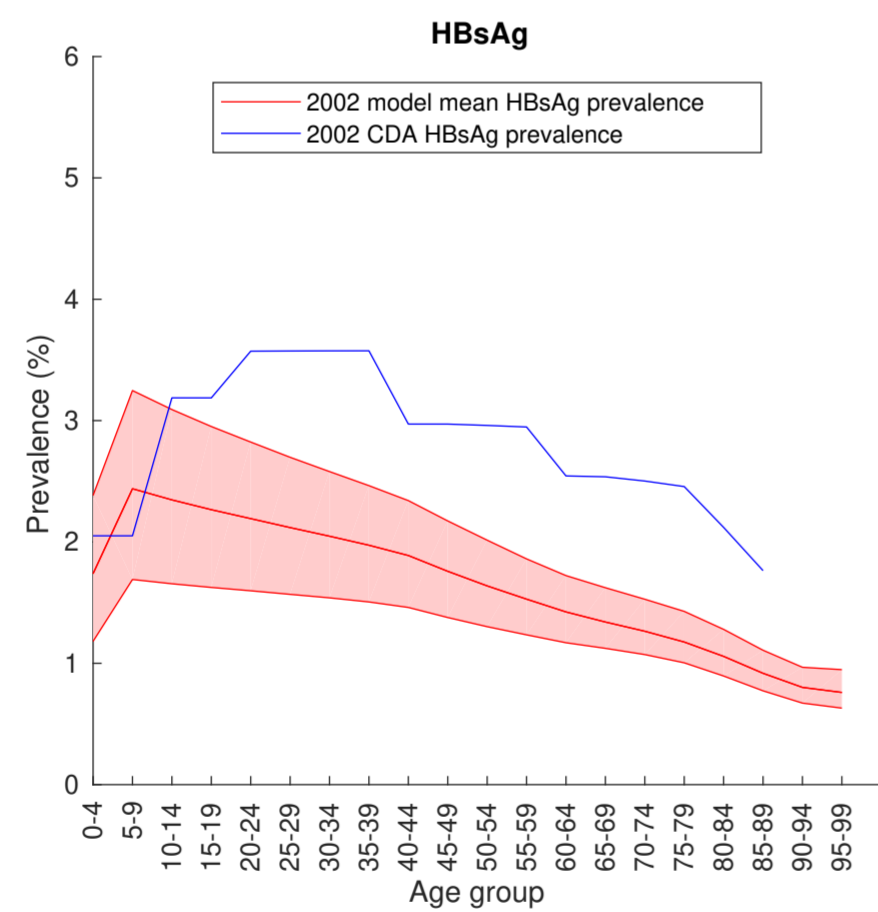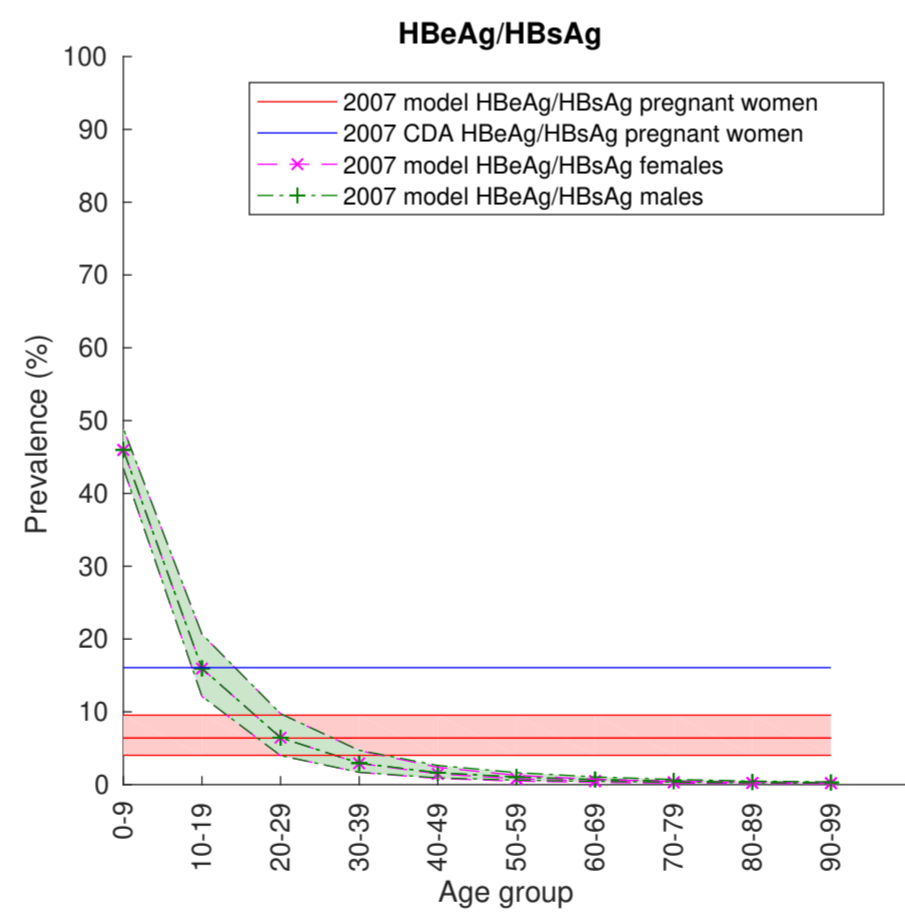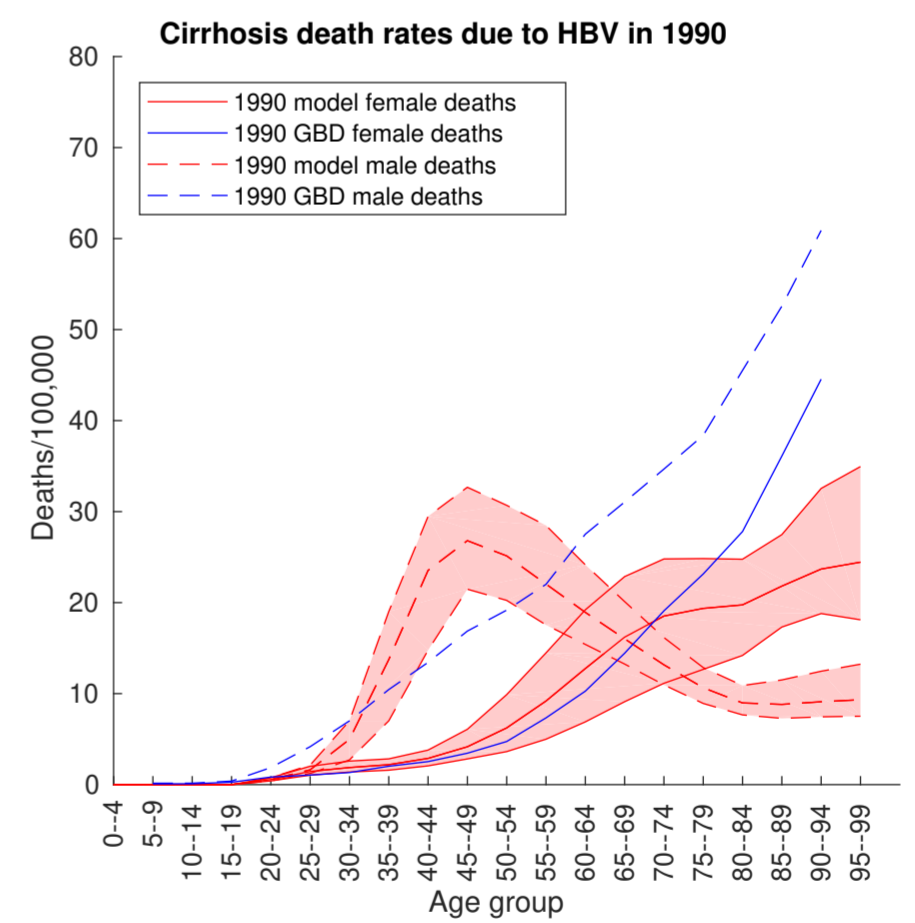

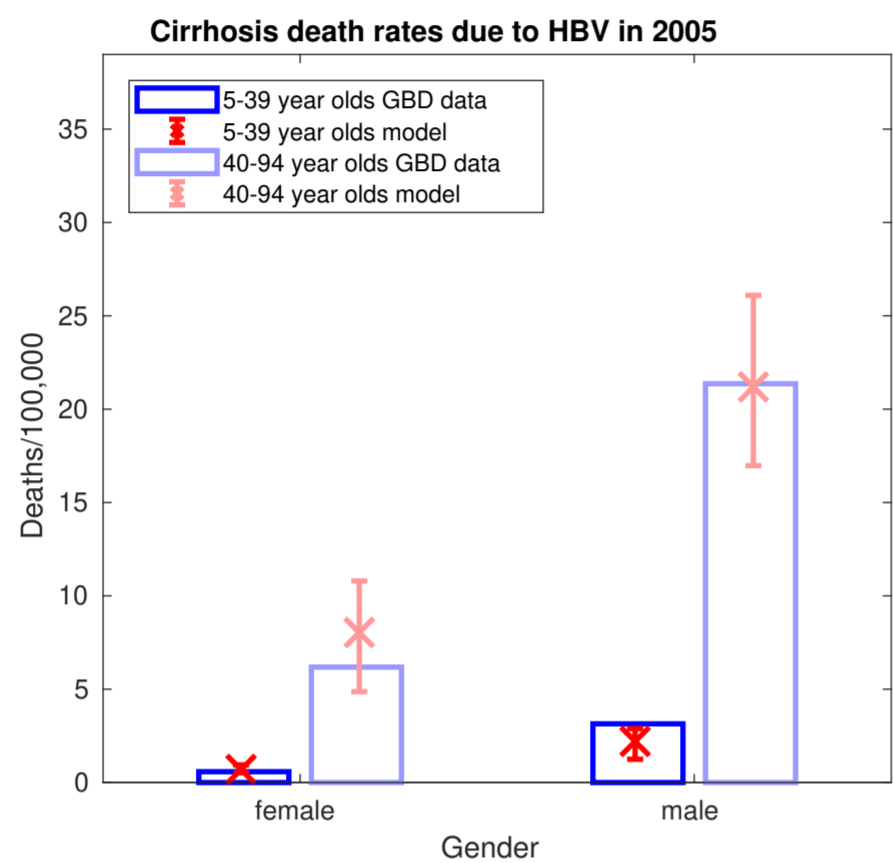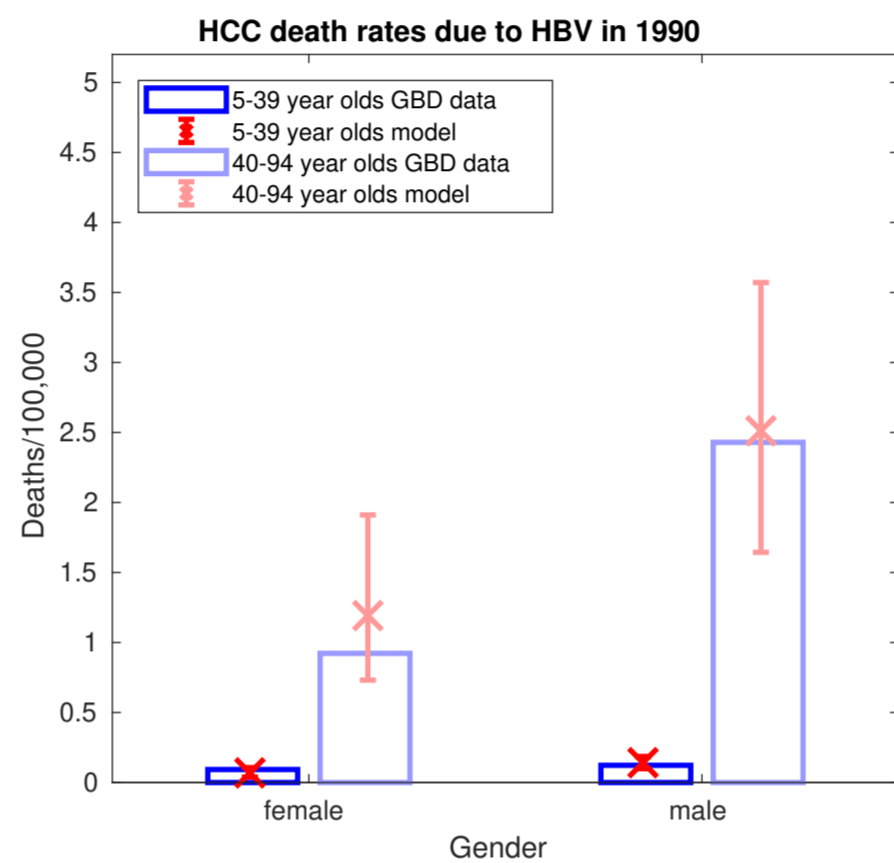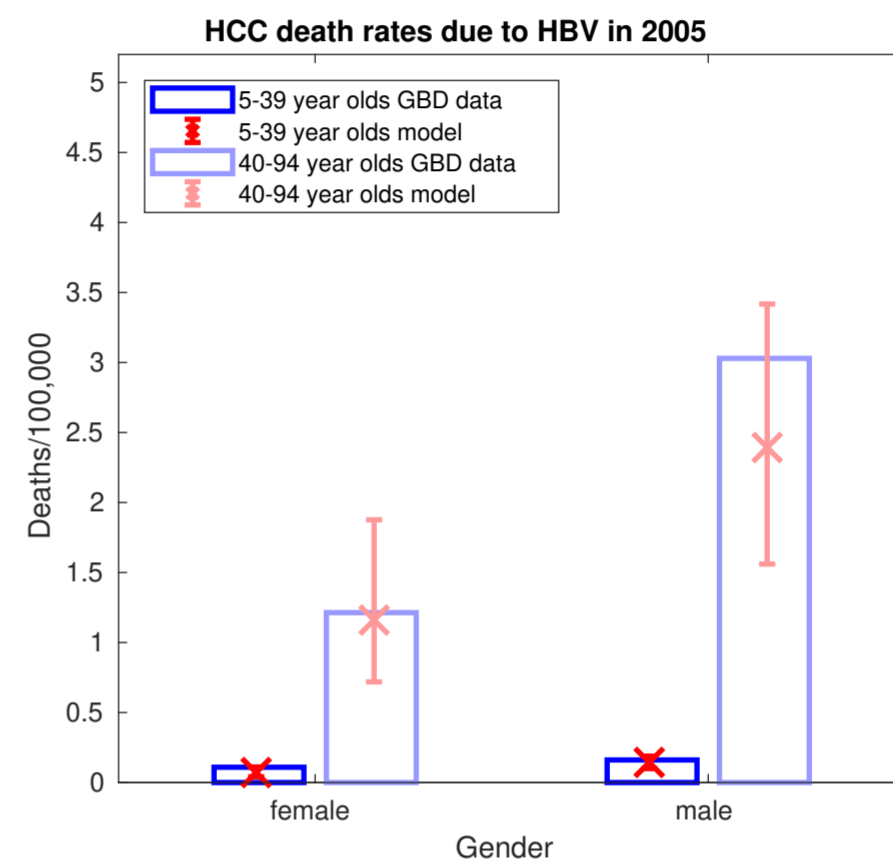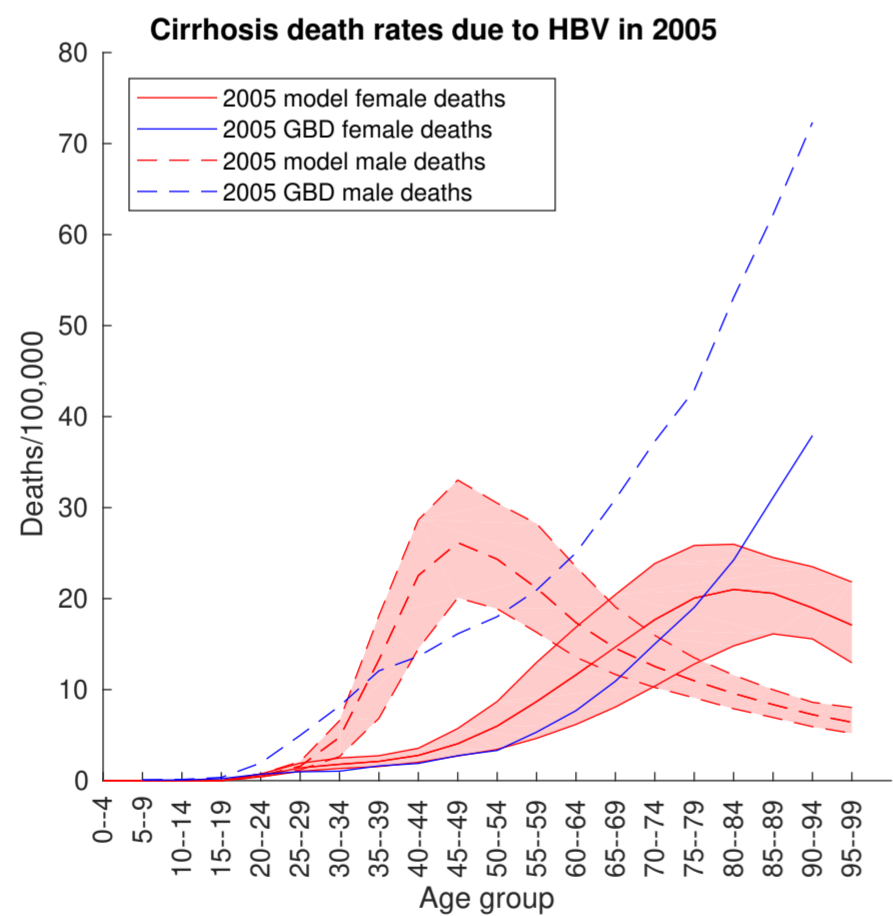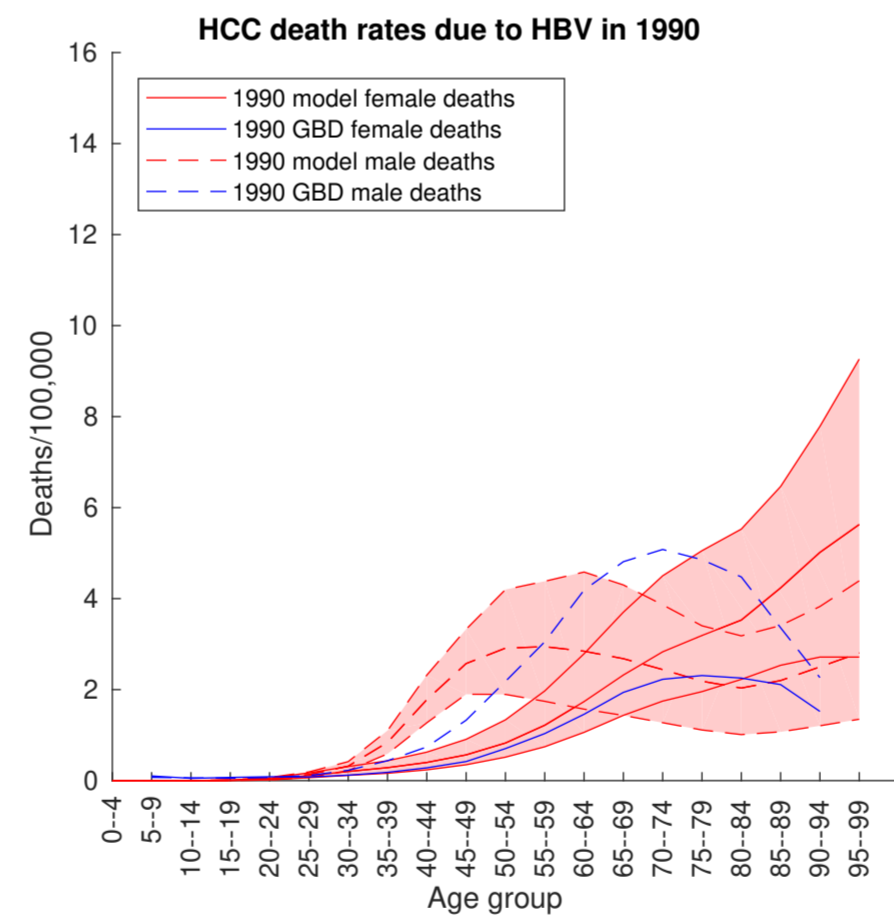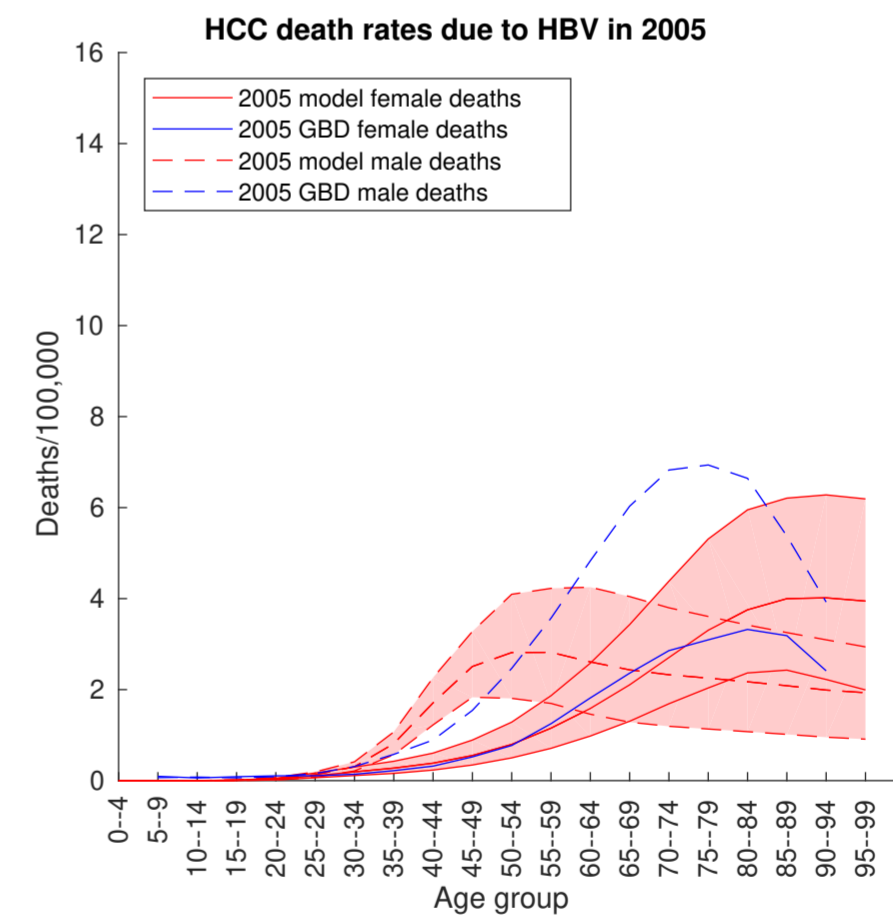

Supplement: S3 Fig — Confidence bands of the 2.5 and 97.5 percentiles are shown. CDA: Center for Disease Analysis; GBD: Global Burden of Disease; HBV: hepatitis B virus; HBeAg: hepatitis B e antigen; HBsAg: hepatitis B surface antigen. (PDF) [file pone.0237525.s004.pdf]

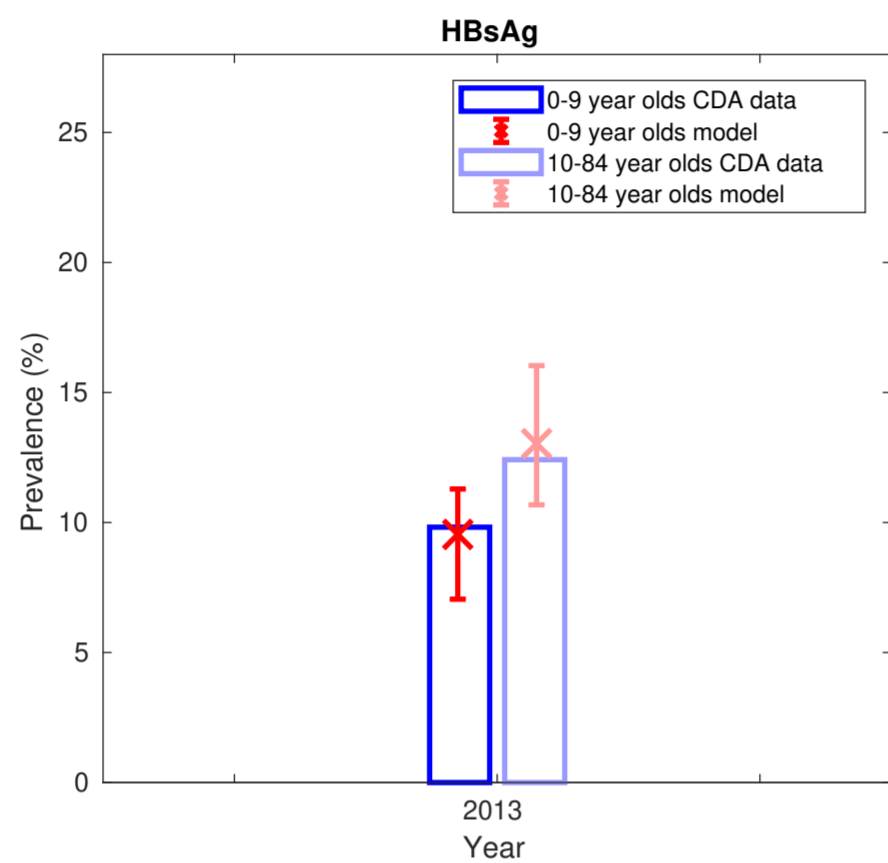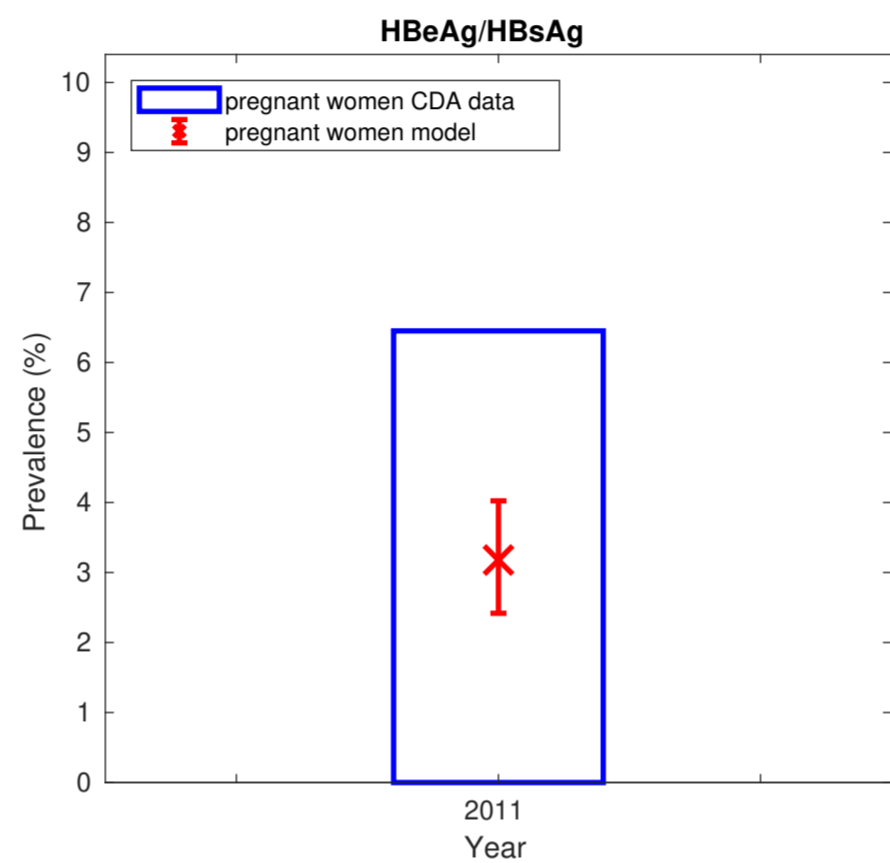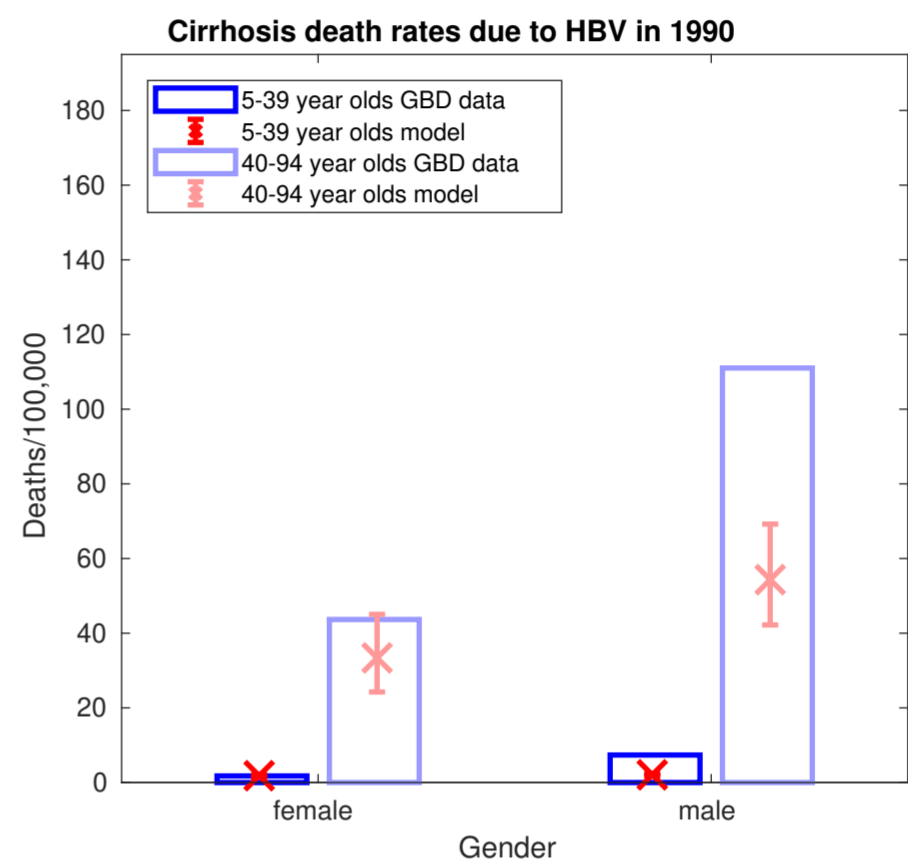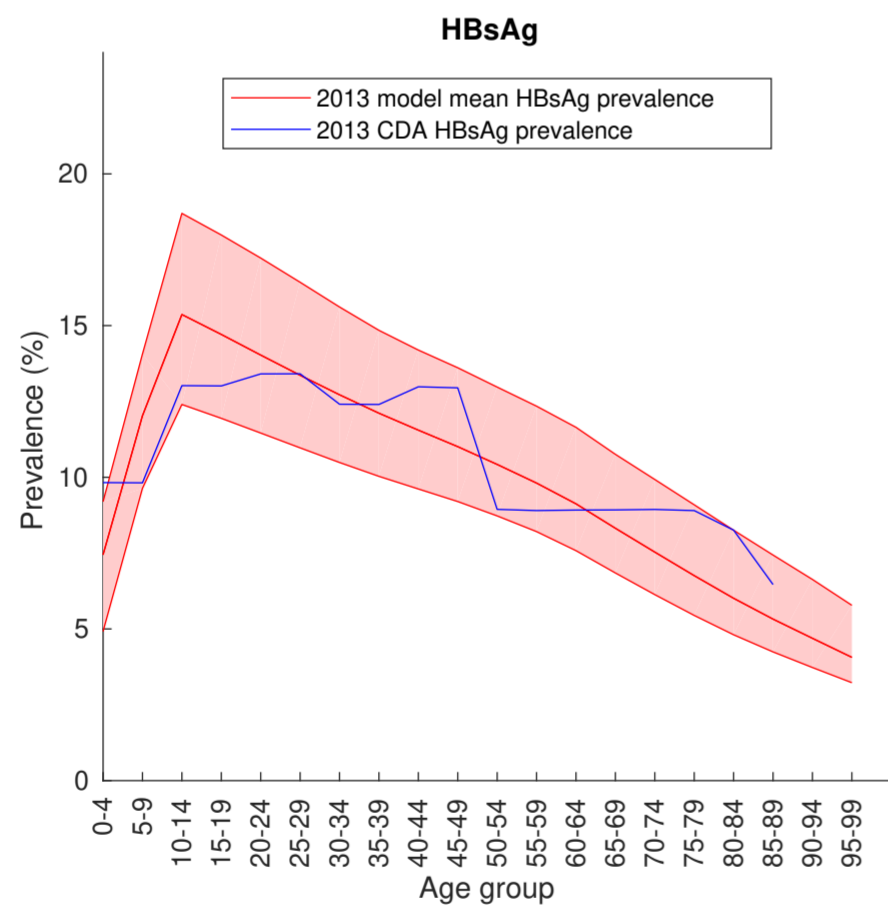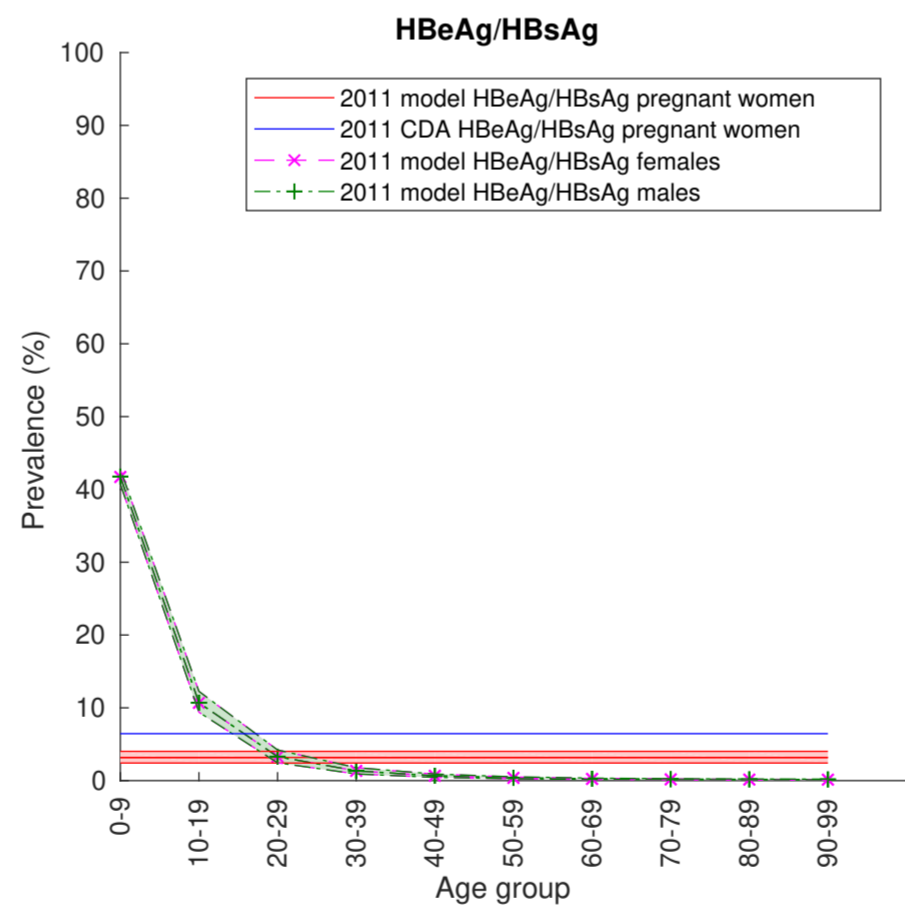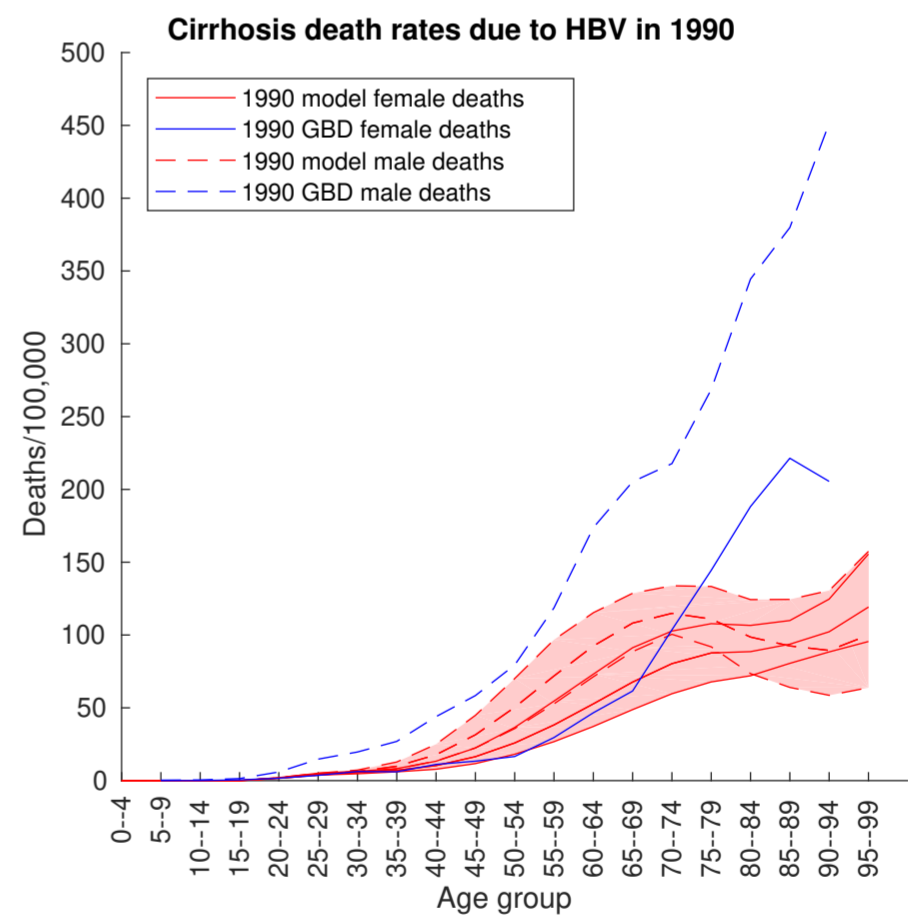

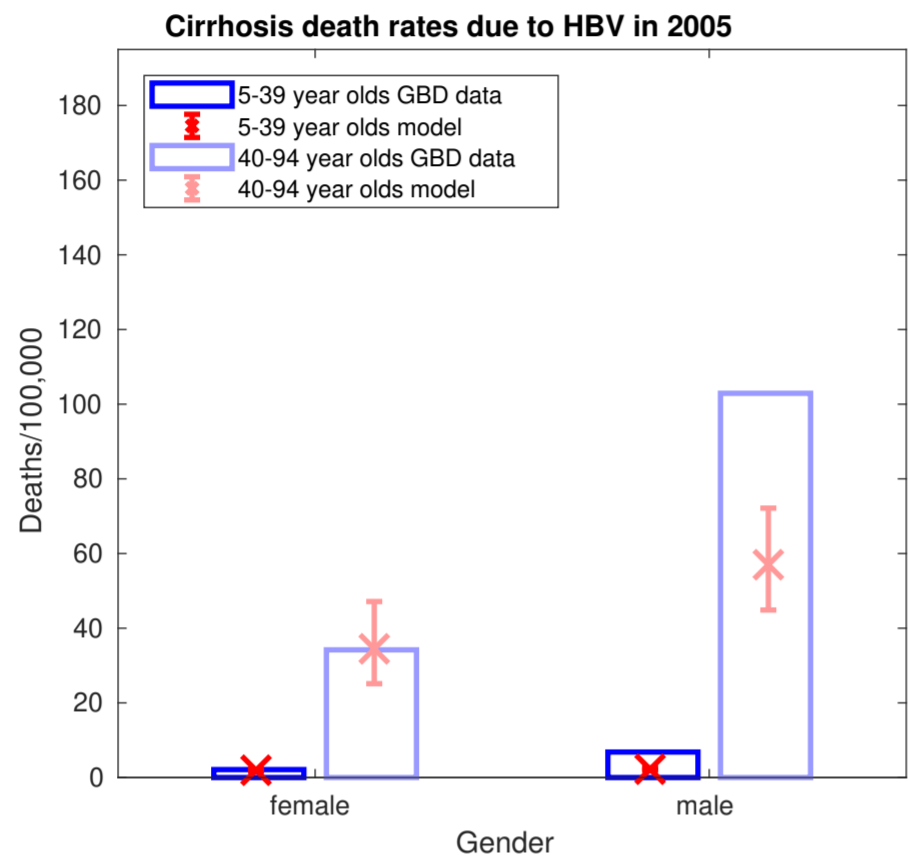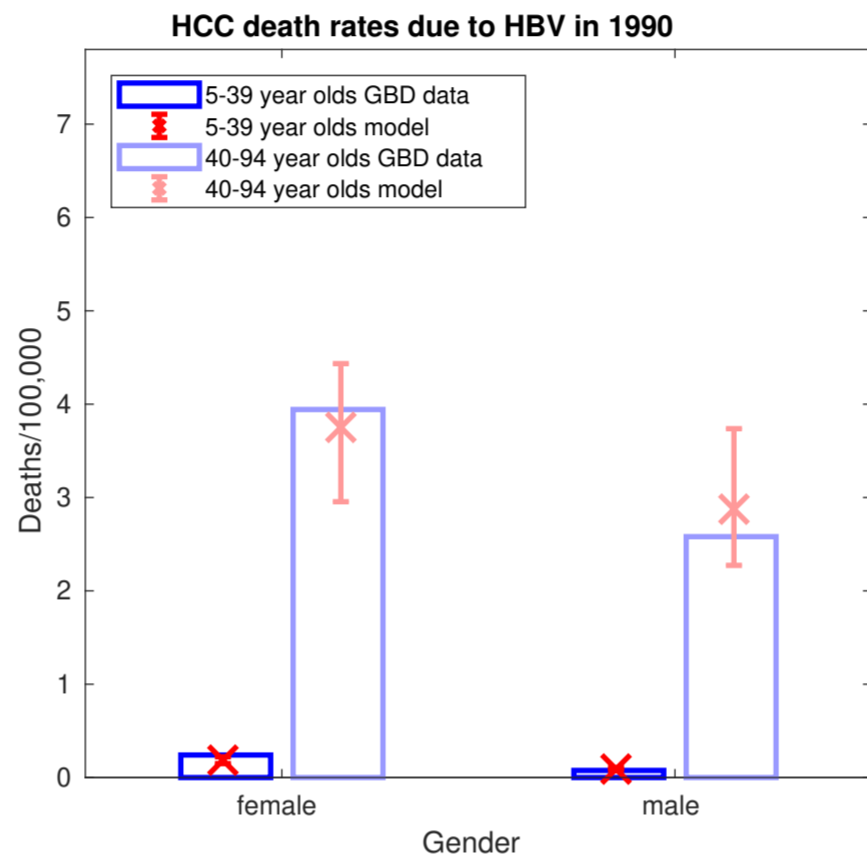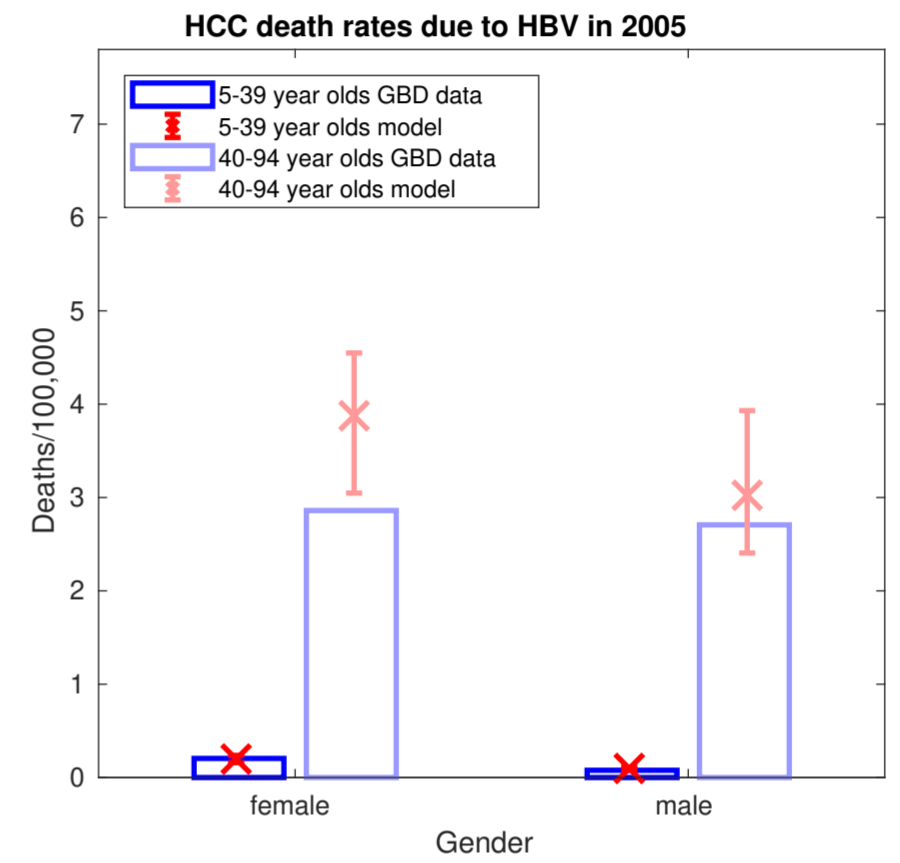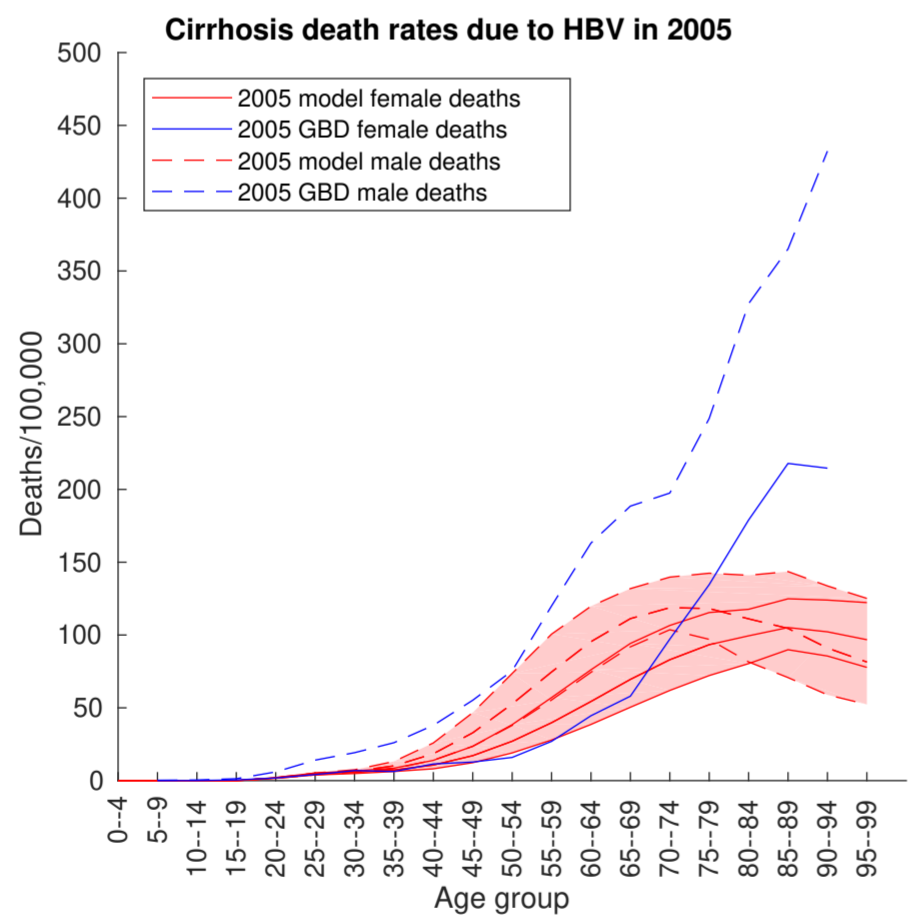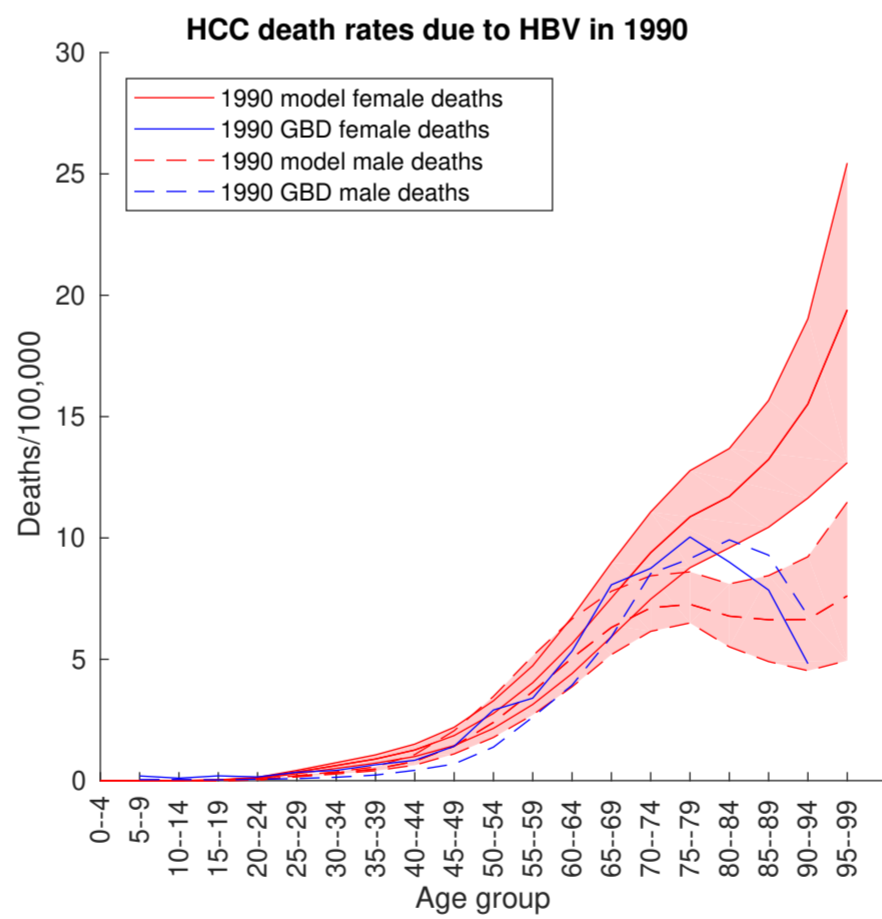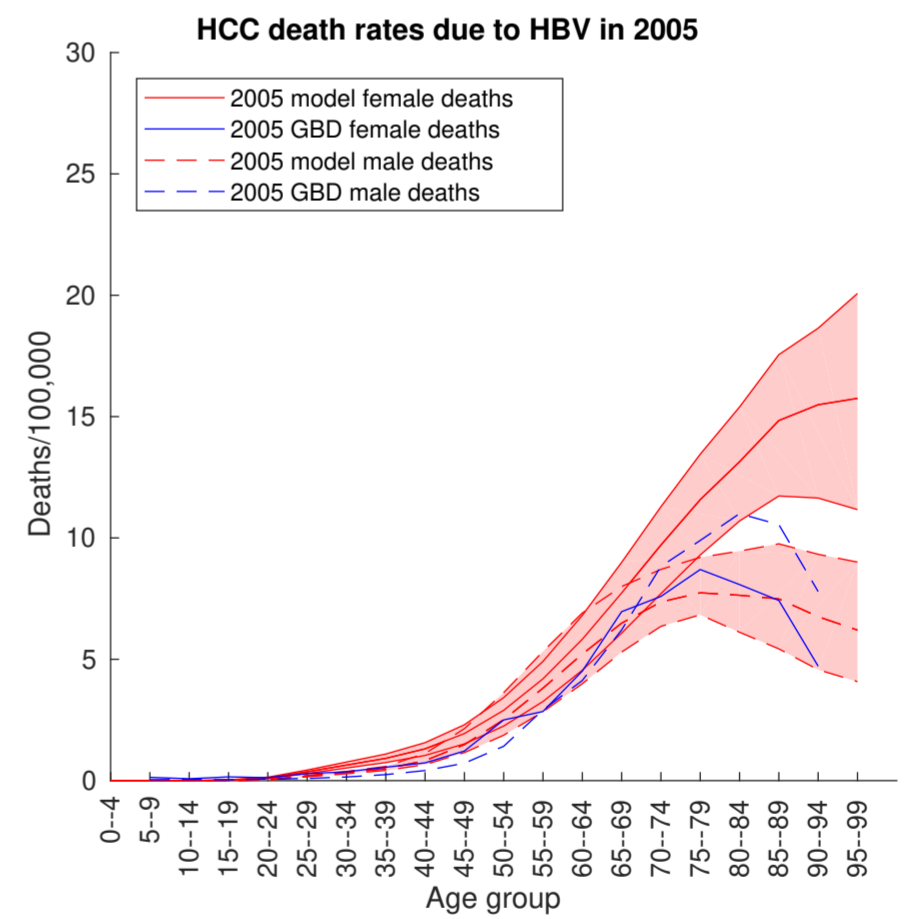

Supplement: S4 Fig — Confidence bands of the 2.5 and 97.5 percentiles are shown. CDA: Center for Disease Analysis; GBD: Global Burden of Disease; HBV: hepatitis B virus; HBeAg: hepatitis B e antigen; HBsAg: hepatitis B surface antigen. (PDF) [file pone.0237525.s005.pdf]

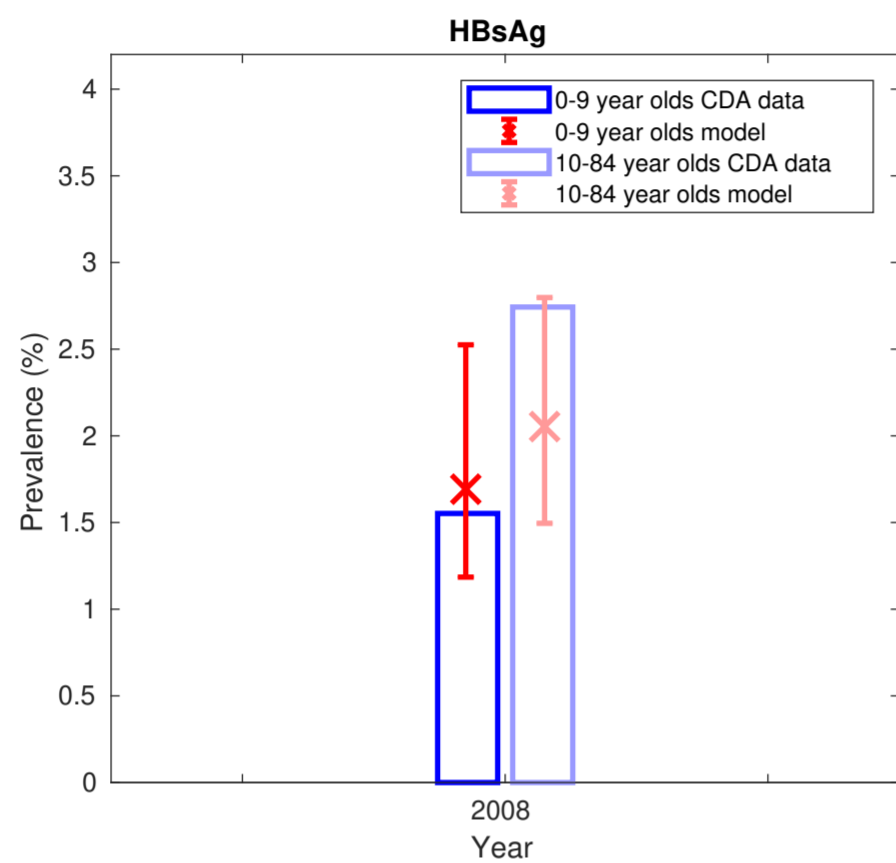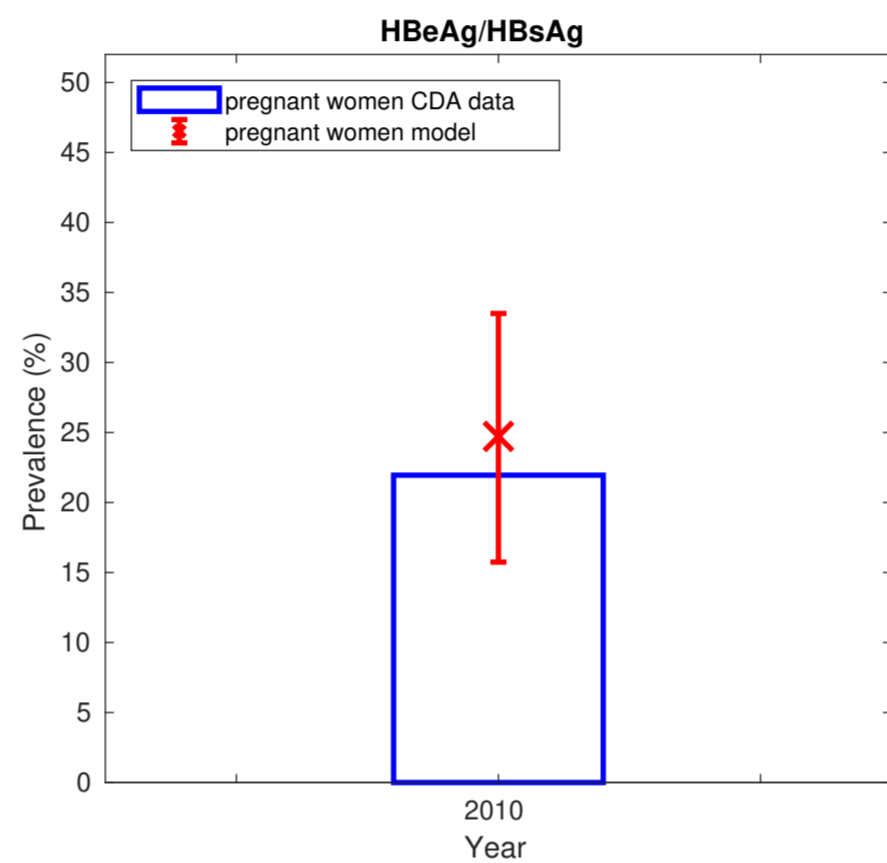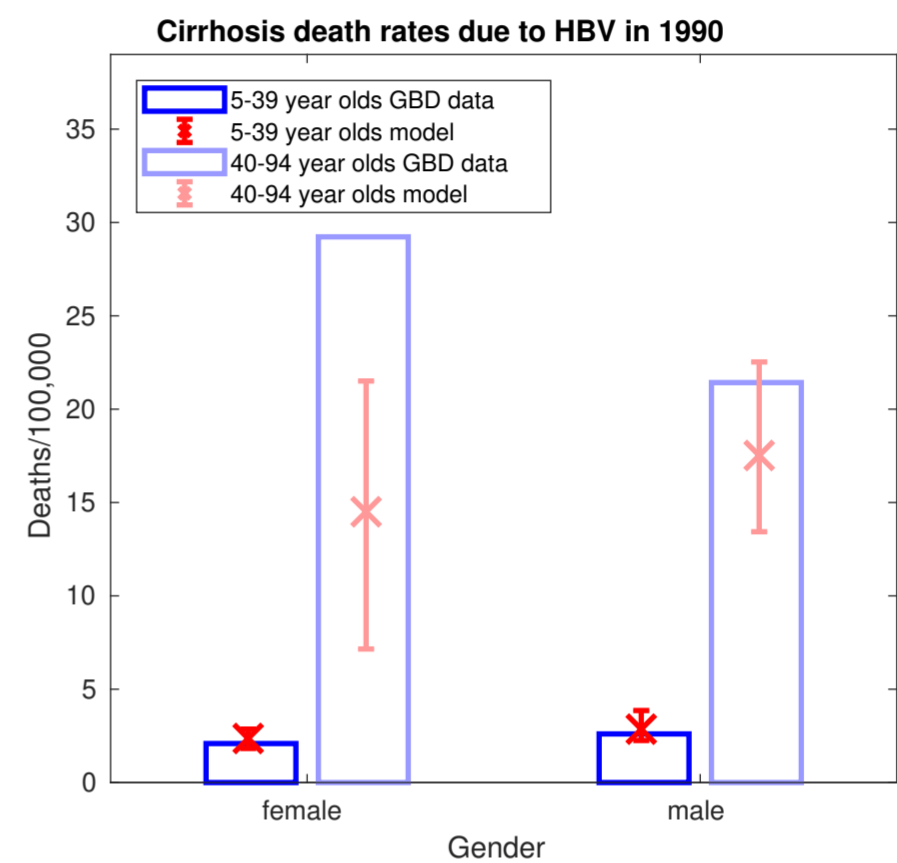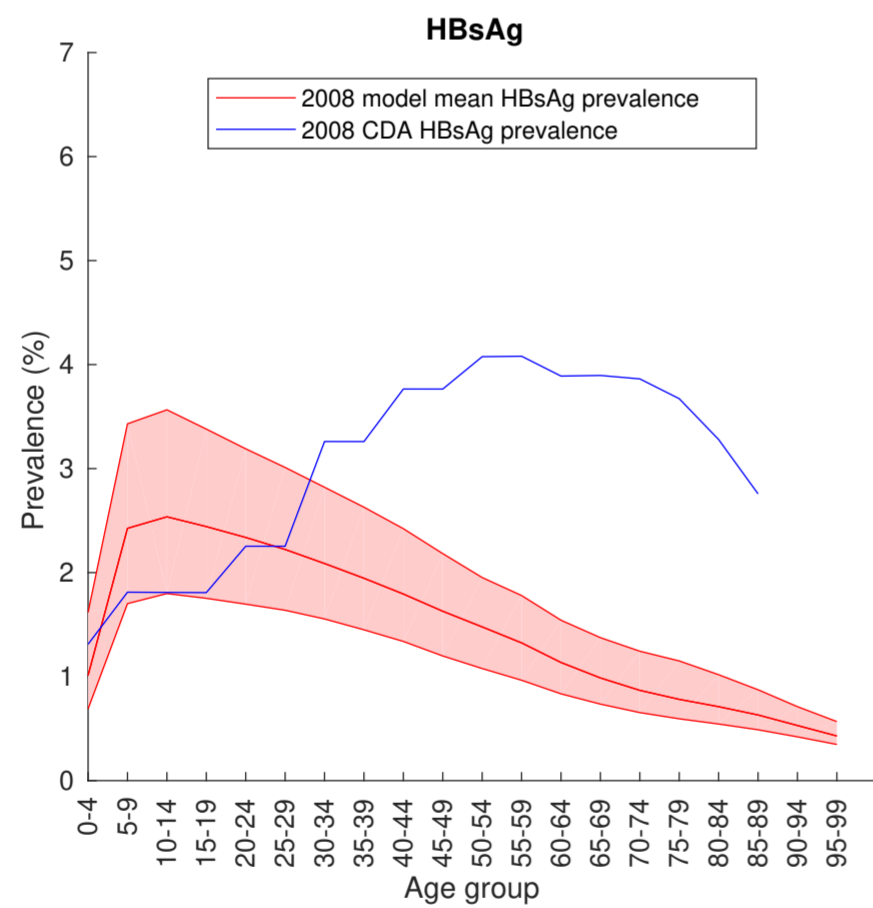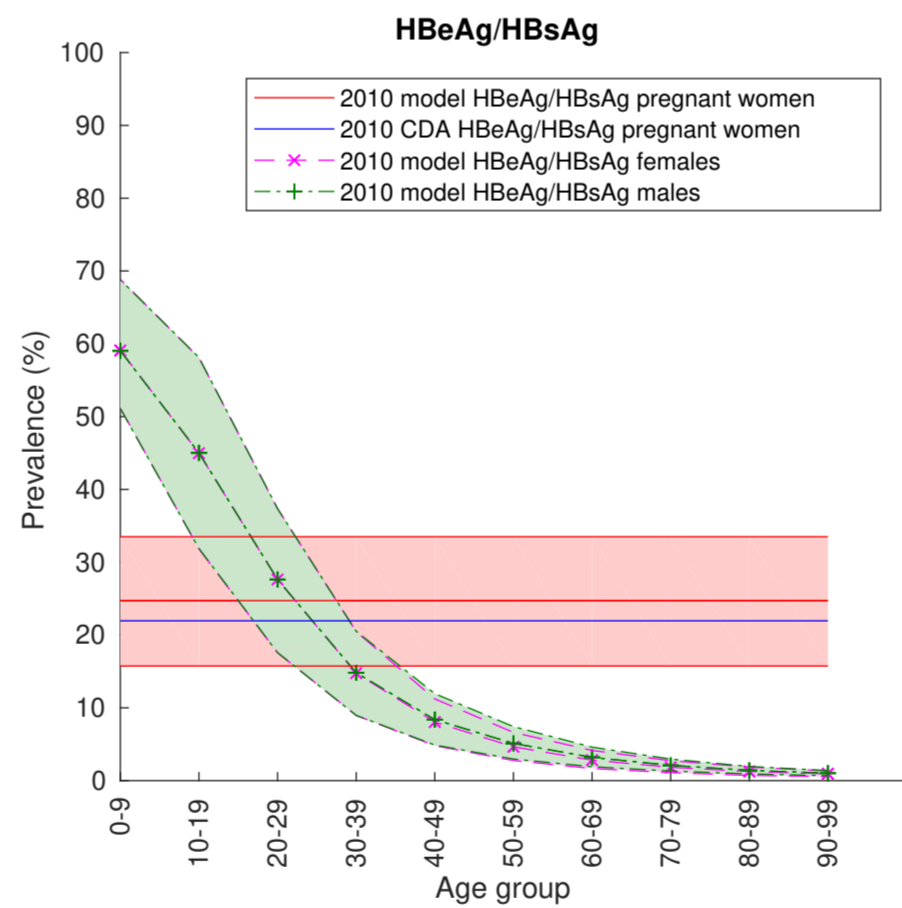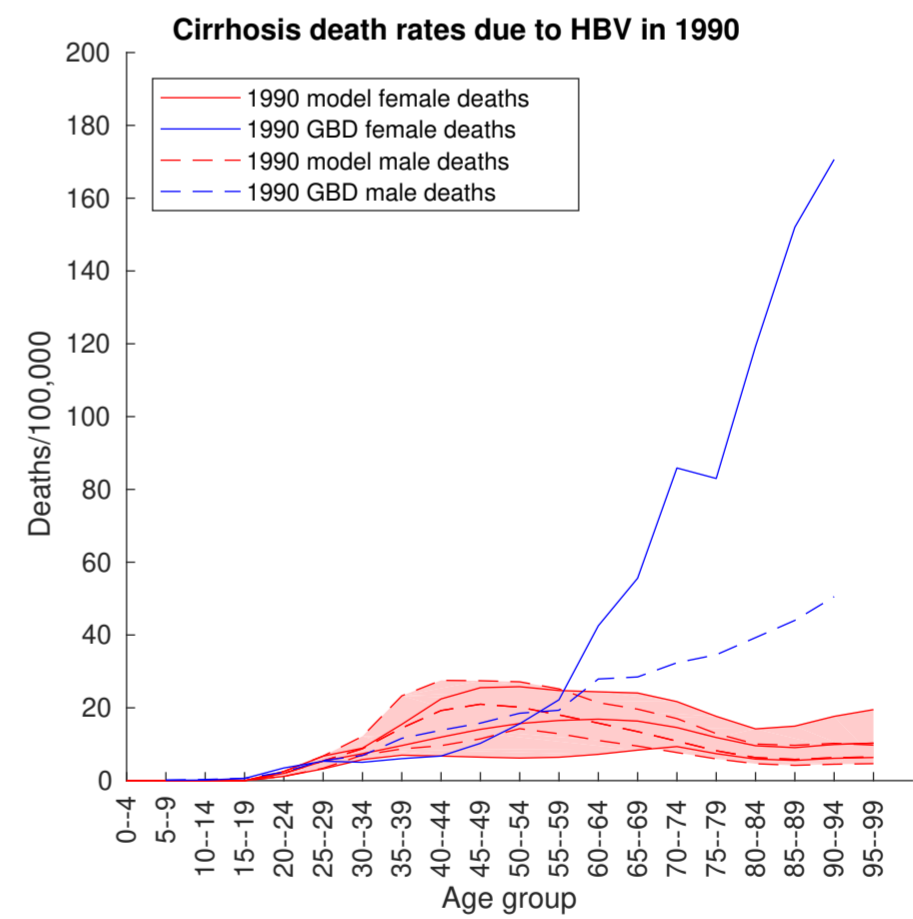

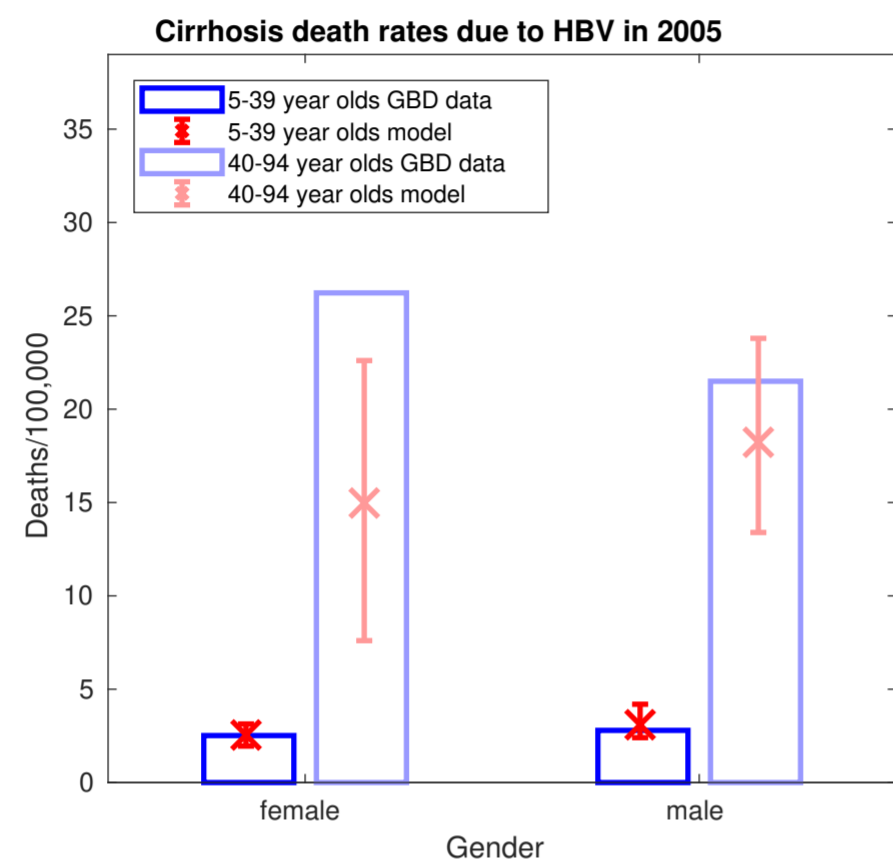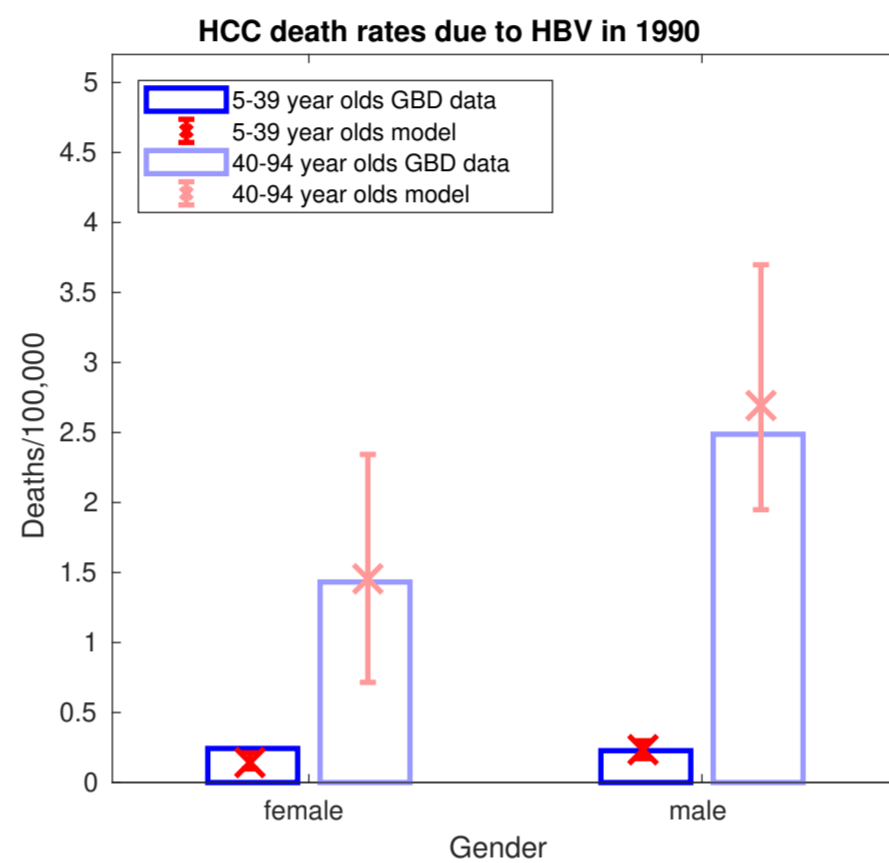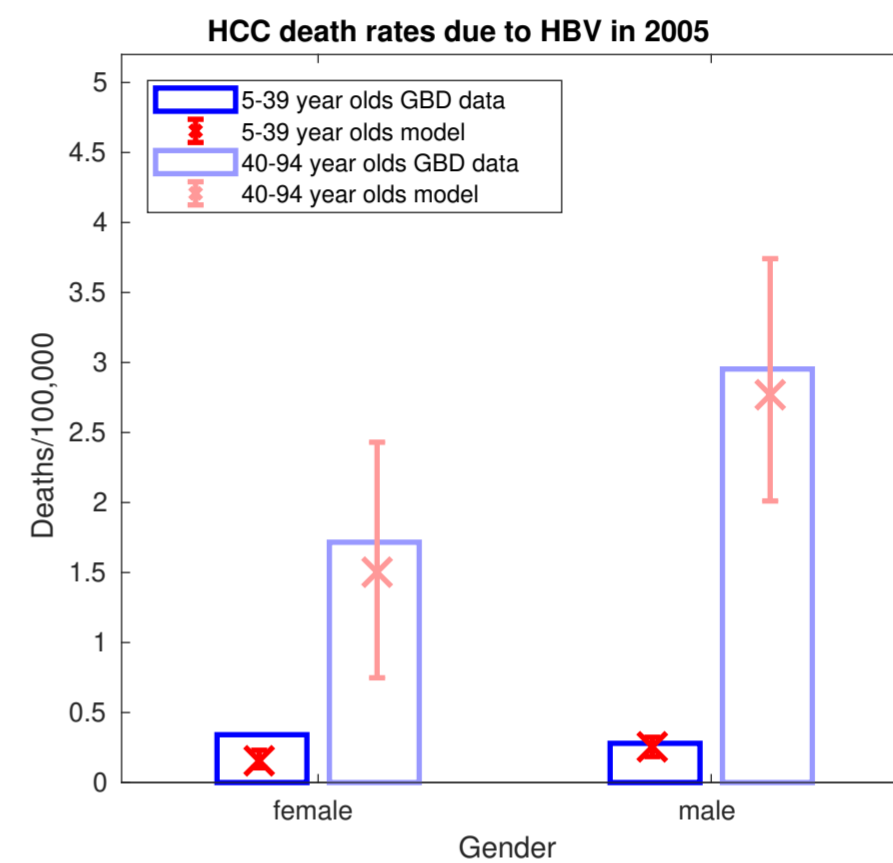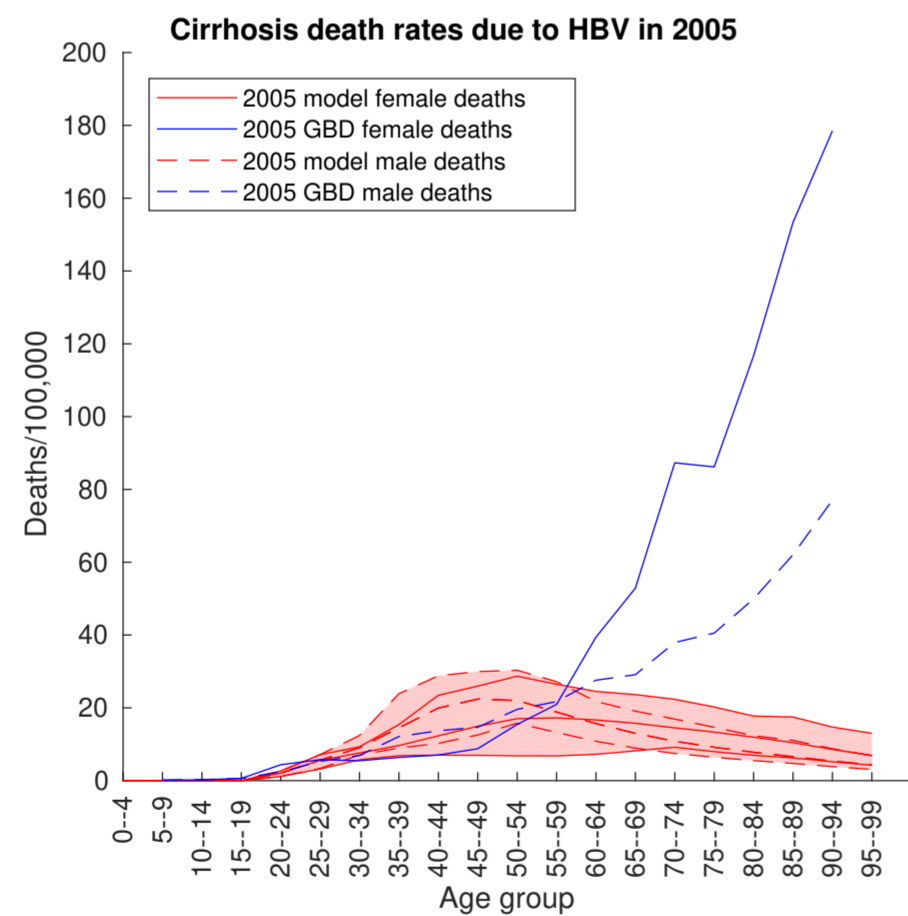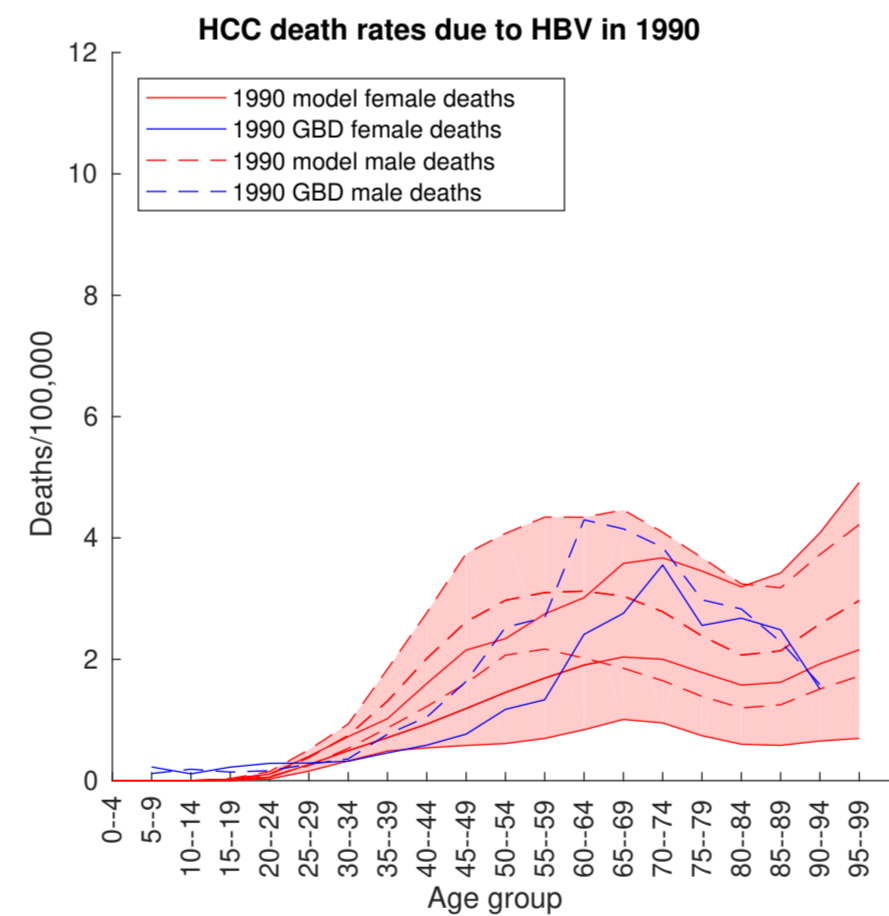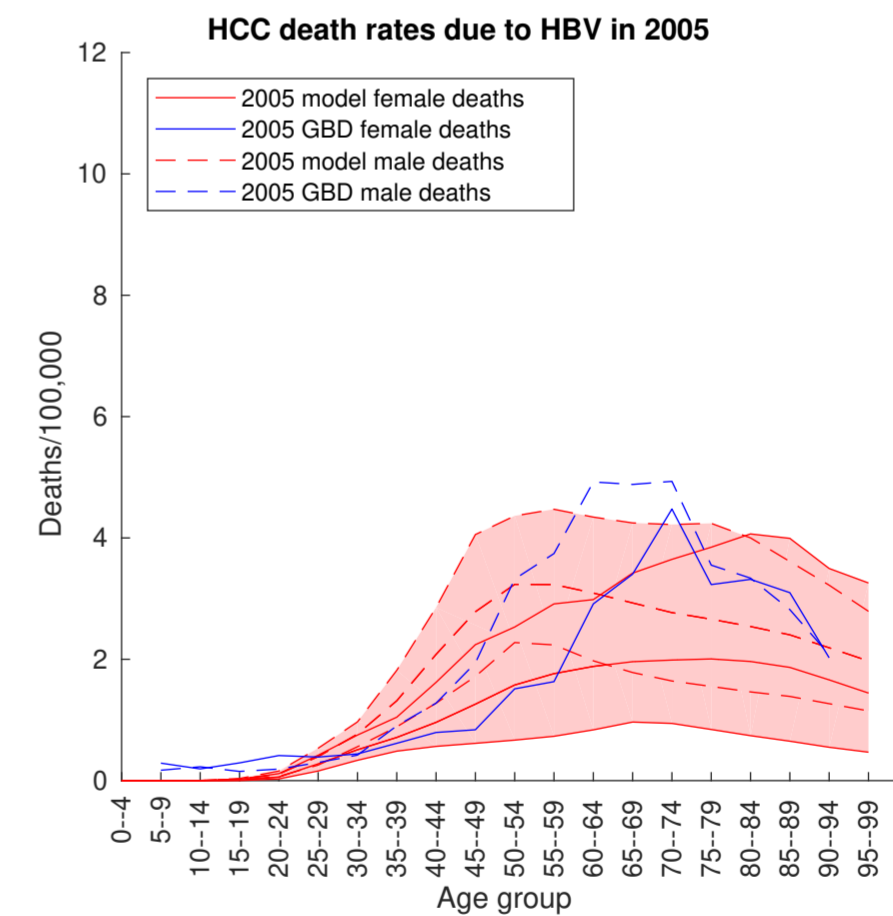

Supplement: S5 Fig — Confidence bands of the 2.5 and 97.5 percentiles are shown. CDA: Center for Disease Analysis; GBD: Global Burden of Disease; HBV: hepatitis B virus; HBeAg: hepatitis B e antigen; HBsAg: hepatitis B surface antigen. (PDF) [file pone.0237525.s006.pdf]

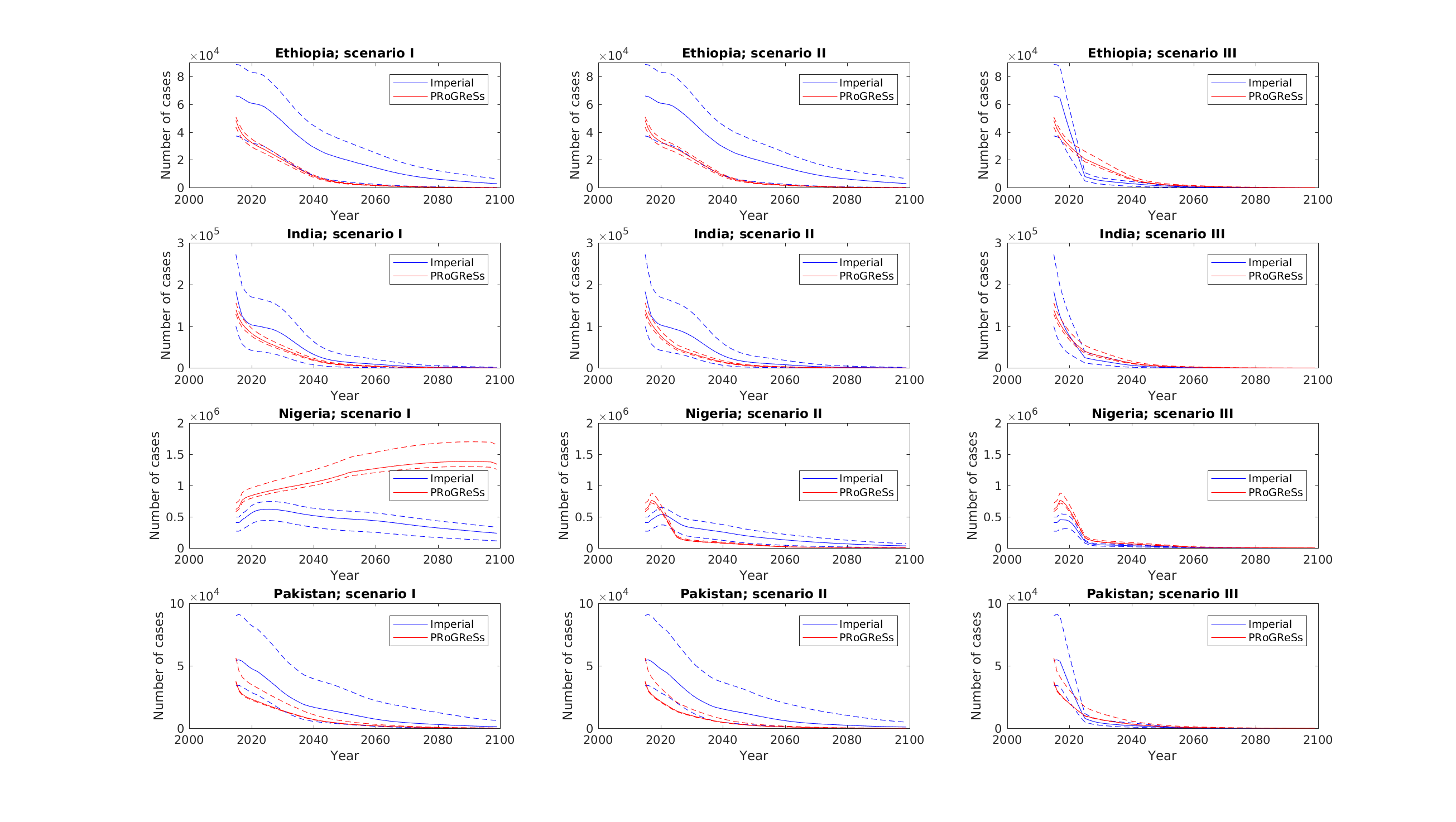

Supplement: S6 Fig — (TIF) [file pone.0237525.s007.tif]

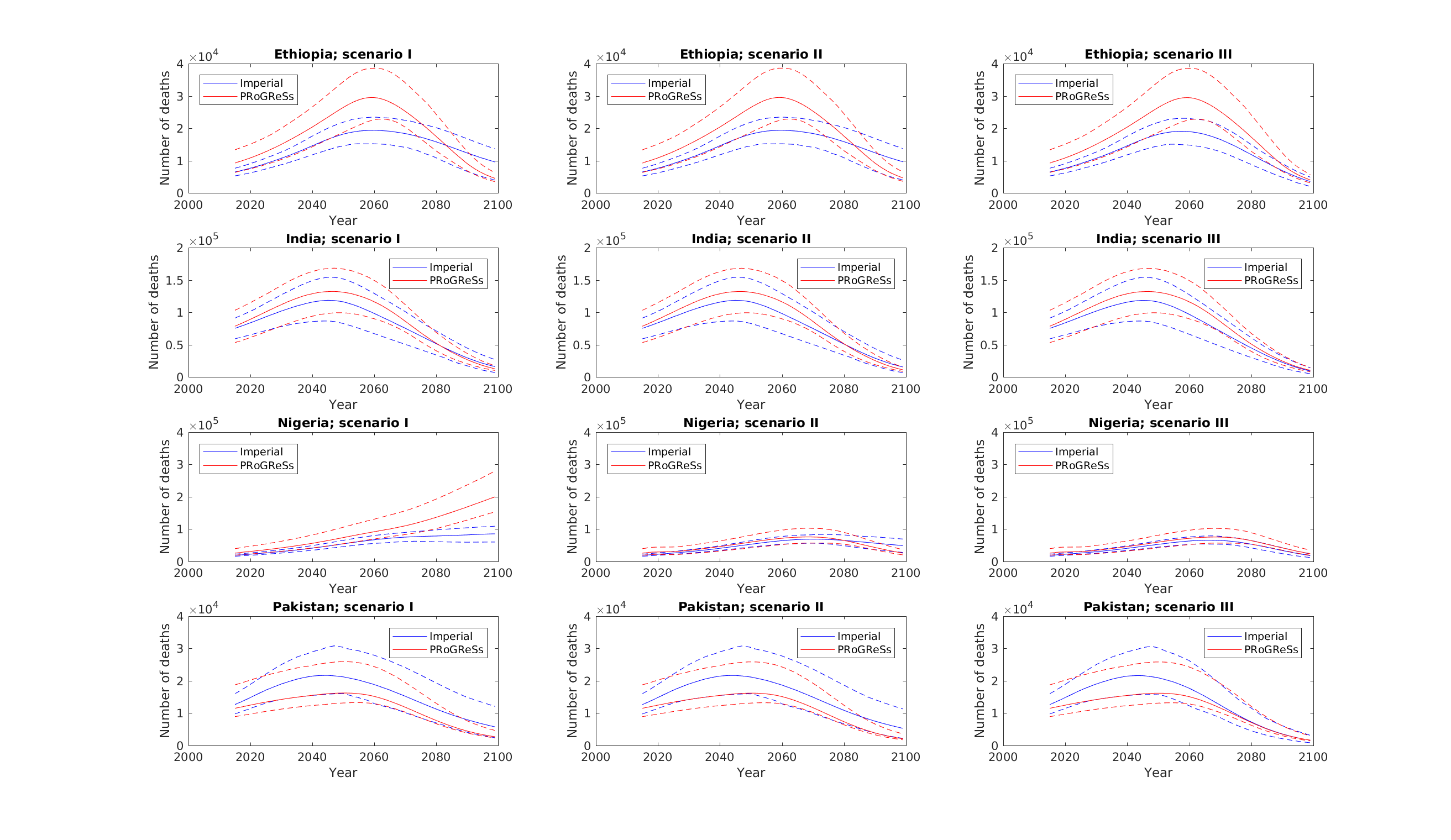

Supplement: S7 Fig — (TIF) [file pone.0237525.s008.tif]

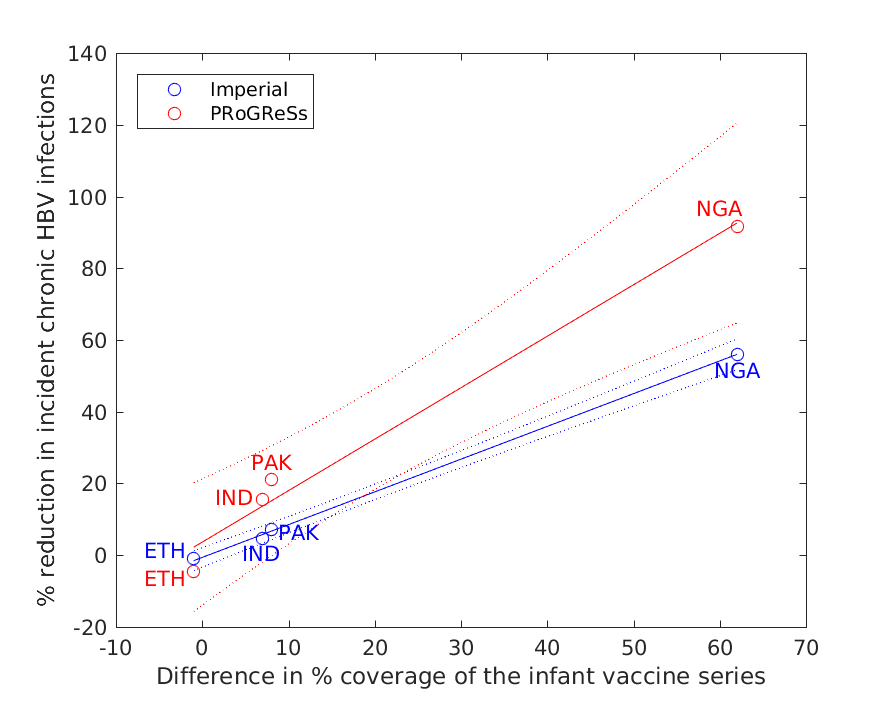

Supplement: S8 Fig — ETH: Ethiopia, IND: India, NGA: Nigeria, PAK: Pakistan. (TIF) [file pone.0237525.s009.tif]

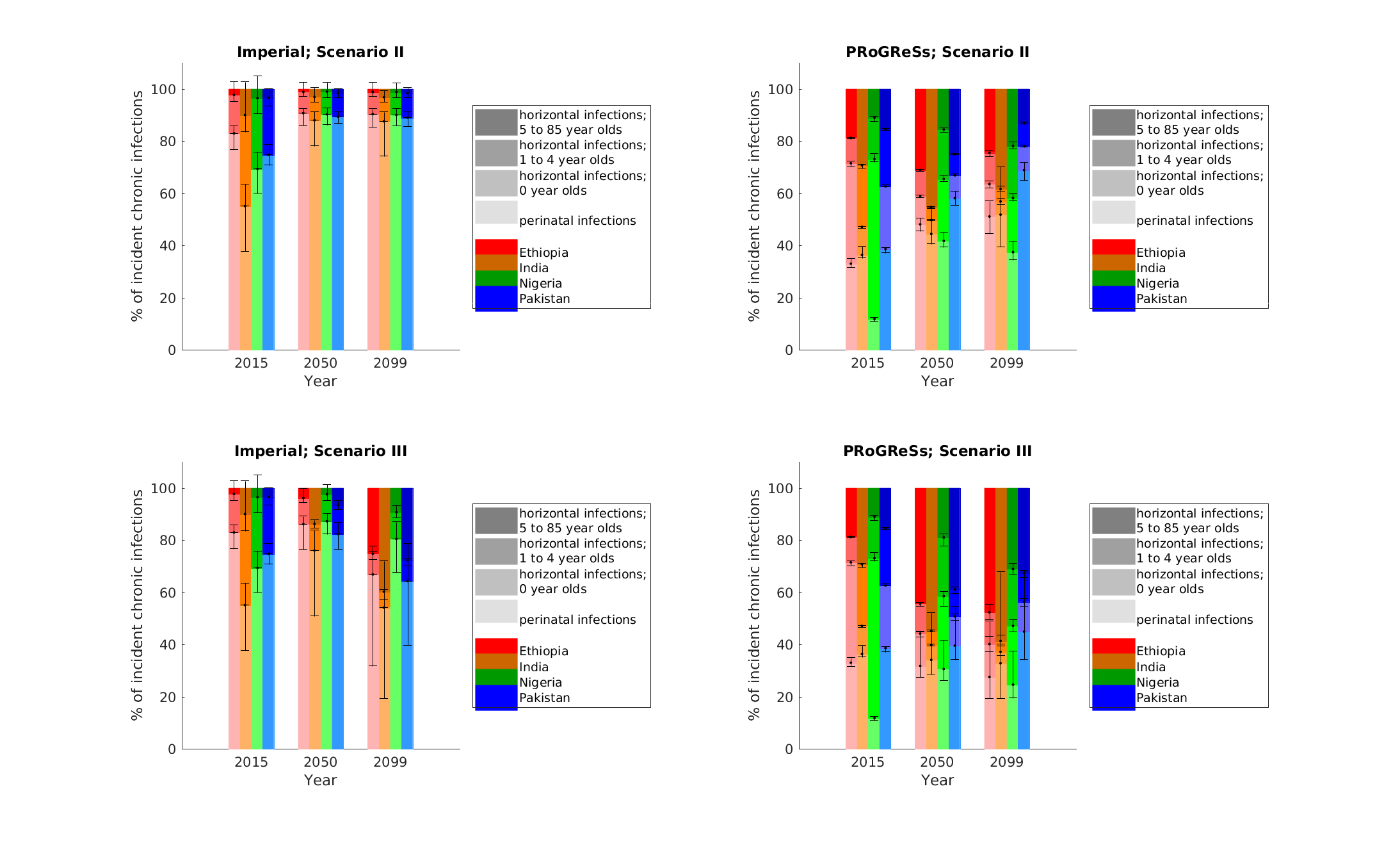

Supplement: S9 Fig — (TIF) [file pone.0237525.s010.tif]

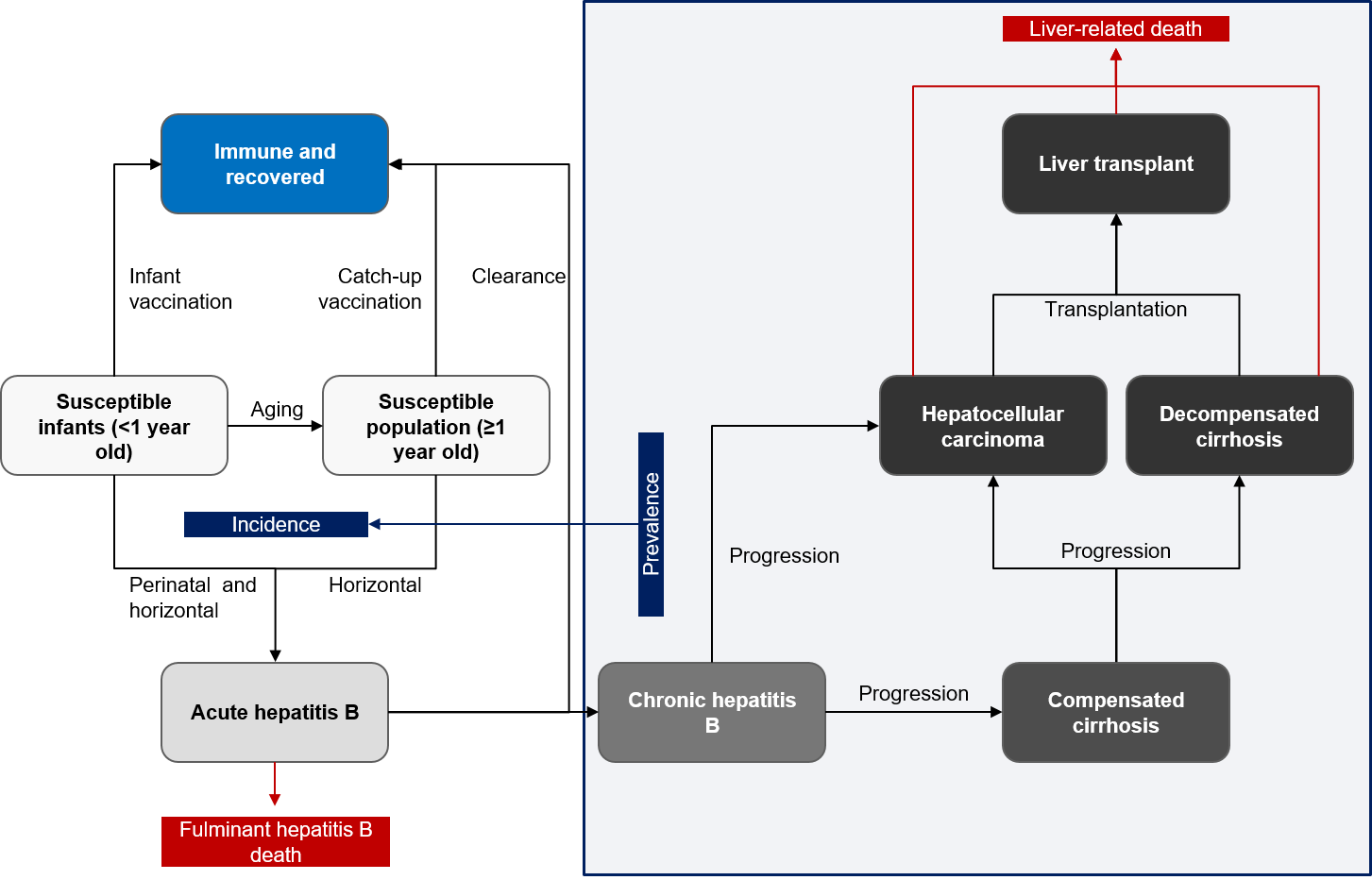

Supplement: S10 Fig — (TIF) [file pone.0237525.s011.tif]
